# Tetrahydroimidazo[1,2‐*a*]pyrazine Derivatives: Synthesis and Evaluation as Gα_q_‐Protein Ligands

**DOI:** 10.1002/chem.202001446

**Published:** 2020-09-07

**Authors:** Jim Küppers, Tobias Benkel, Suvi Annala, Kenichi Kimura, Lisa Reinelt, Bernd K. Fleischmann, Evi Kostenis, Michael Gütschow

**Affiliations:** ^1^ Pharmaceutical Institute Department of Pharmaceutical & Medicinal Chemistry University of Bonn An der Immenburg 4 53121 Bonn Germany; ^2^ Molecular, Cellular and Pharmacobiology Section Institute for Pharmaceutical Biology University of Bonn Nussallee 6 53115 Bonn Germany; ^3^ Research Training Group 1873 University of Bonn 53115 Bonn Germany; ^4^ Institute of Physiology I, Life and Brain Center, Medical Faculty University of Bonn Sigmund-Freud-Str. 25 53105 Bonn Germany

**Keywords:** Davidson cyclization, G proteins, lactamization, structure–activity relationships, toxicity

## Abstract

The 5,6,7,8‐tetrahydroimidazo[1,2‐*a*]pyrazine derivative BIM‐46174 and its dimeric form BIM‐46187 (**1**) are heterocyclized dipeptides that belong to the very few cell‐permeable compounds known to preferentially silence Gα_q_ proteins. To explore the chemical space of Gα_q_ inhibitors of the BIM chemotype, a combinatorial approach was conducted towards a library of BIM molecules. This library was evaluated in a second messenger‐based fluorescence assay to analyze the activity of Gα_q_ proteins through the determination of intracellular *myo*‐inositol 1‐phosphate. Structure–activity relationships were deduced and structural requirements for biological activity obtained, which were (i) a redox reactive thiol/disulfane substructure, (ii) an *N*‐terminal basic amino group, (iii) a cyclohexylalanine moiety, and (iv) a bicyclic skeleton. Active compounds exhibited cellular toxicity, which was investigated in detail for the prototypical inhibitor **1**. This compound affects the structural cytoskeletal dynamics in a Gα_q/11_‐independent manner.

## Graphical abstract

**G proteins**: Different points of diversity (see colors) were introduced into the full‐size BIM structure to explore the chemical space of G protein inhibitors of the BIM chemotype. Requirements for biological activity are a redox reactive thiol/disulfane substructure, an *N*‐terminal basic amino group, a cyclohexylalanine moiety, and a bicyclic skeleton.

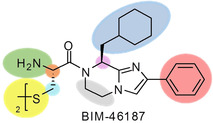

## Introduction

G protein‐coupled receptors (GPCRs), also known as seven‐transmembrane receptors, represent the largest family of cell‐surface receptors in eukaryotes.^1^ GPCR activation initiates a plethora of intracellular multistep signaling events.^2^ About one third of prescription drugs on the market target GPCRs either as agonists or antagonists.^3^ Upon agonist binding, GPCRs undergo conformational changes resulting in the activation of receptor‐associated heterotrimeric guanine nucleotide‐binding proteins (G proteins), which consist of three different subunits referred to as α, β and γ.^4^ The capability of acting as molecular switches, thus transducing extracellular signals via GPCRs into intracellular signal cascades, makes heterotrimeric G proteins vitally important.^5^ So far, GPCRs rather than their associated G proteins have been targeted in contemporary drug development. For pathologies, which are characterized by the dysregulation of one specific receptor, the manipulation of an individual GPCR is suitable. However, in case of complex disorders, such as cancer or pain, which involve multiple receptors and their associated pathways, the interference at the post‐receptor level appears to be particularly promising and the intracellular G proteins can thus be envisaged to serve as potential drug targets.^6^, ^7^ Furthermore, the direct influence on specific G proteins may constitute a useful pharmacological strategy to unravel their role under physiological and pathophysiological conditions. According to the amino acid sequence homology of the Gα subunits, heterotrimeric G proteins are subdivided into four families, Gα_s_, Gα_i/o_, Gα_q_ and Gα_12/13_. The Gα_q_ family comprises four members, that is, Gα_q_, Gα_11_, Gα_14_ and Gα_15/16_, among which the isoforms Gα_q_ and Gα_11_ are most crucial and ubiquitously expressed.^8^, ^9^ In their customary role, Gα_q_ family members activate their major downstream effector, the enzyme phospholipase C‐β (PLCβ), leading to hydrolysis of membrane‐bound phosphatidylinositol‐4,5‐bisphosphate (PIP2) into diacylglycerol (DAG), which in turn activates protein kinase C, and into *myo*‐inositol 1,4,5‐trisphosphate (IP3), which initiates the release of calcium ions from the endoplasmic or sarcoplasmic reticulum into the cytosol by opening IP3‐sensitive calcium channels.^7^, ^8^

**Scheme 1:**
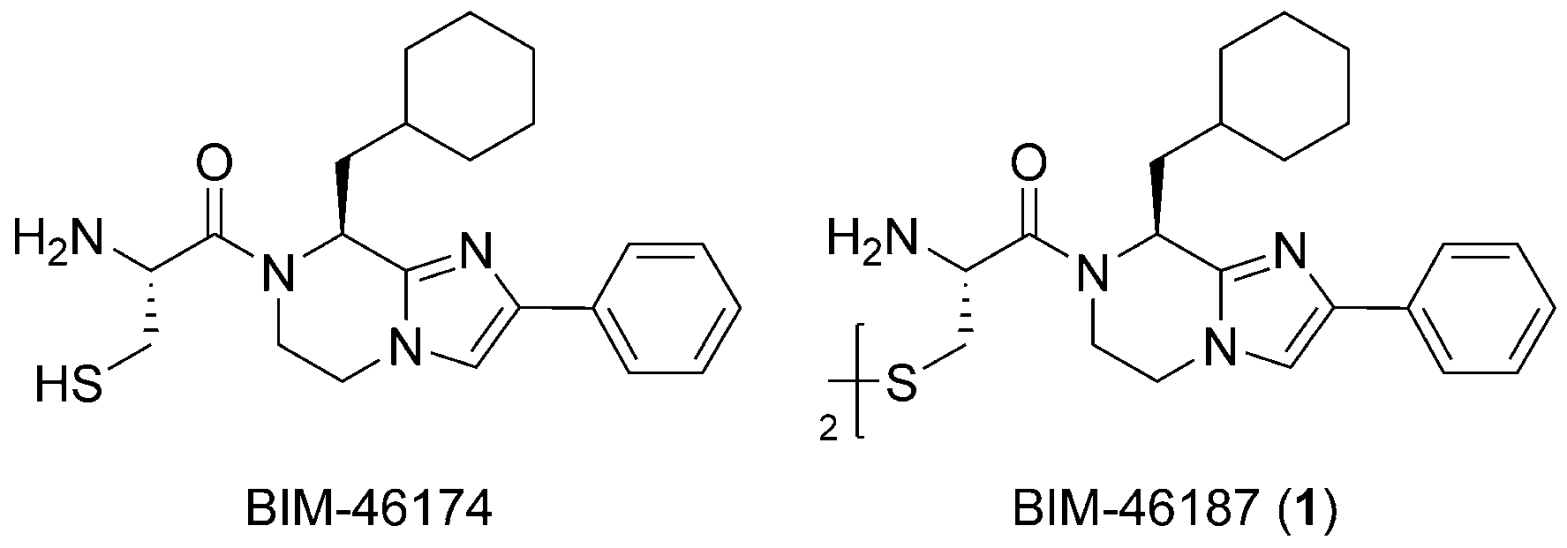
Structures of the monomeric and dimeric BIM molecules.

The modulation of the Gα_q_ protein subfamily is of particular relevance, since its members account for a wide variety of cellular responses,^7^, ^10^ leading to important physiological functions, such as platelet aggregation,^11^ insulin‐stimulated glucose transport,^12^ as well as pathophysiological consequences, such as heart failure,^13^ and cancer.^6^, ^9^, ^14^ Hence, selective and potent modulators are highly required as tool compounds to investigate G protein‐mediated signaling or as feasible drug candidates. Prominent modulators of Gα_q_ proteins include, on the one hand, isolated natural products, YM‐254890 and FR900359, and on the other hand, molecules gained from organic syntheses, the BIM molecules (BIM‐46174 and BIM‐46187) and 27‐mer(I860A), all of which represent selective inhibitors for Gα_q_.^7^ Among these, the cyclic depsipeptides YM‐254890 and FR900359, obtained from the fermentation broth of *Chromobacterium* sp. QS3666 or from the leaves of the plant *Ardisia crenata sims*, respectively, exhibit extraordinary selectivity and receive exceptional attention.^6^, ^7^, ^8^, ^15^, ^16^ The linear peptide 27‐mer(I860A), derived from the phospholipase C‐β isoform PLC‐β3, has also been identified as a selective Gα_q_ inhibitor, however its molecular mechanism of action still remains unclear.^7^, ^17^ The two BIM molecules (Scheme 1) have been previously reported as pan‐G protein inhibitors, corresponding to their ability to likewise inhibit all of the four G‐protein families.^15^, ^18^ This conclusion relied on the compounds’ inhibitory interference with two second messenger pathways, the cyclic adenosine‐3′,5′‐monophosphate (cAMP) and the *myo*‐inositol 1‐phosphate (IP1) pathway, as evaluated in human breast cancer MCF7 and melanoma A2058 cell lines, respectively.^15^, ^18^ Furthermore, both molecules potently inhibited critical functions in cancer progression, in particular cell proliferation and cell survival.^18^, ^19^ A detailed evaluation of BIM action on various GPCR/G protein pairs, co‐expressed in different cellular backgrounds such as Chinese hamster ovary (CHO), human embryonic kidney (HEK) 293, and CV‐1 in origin with SV40 genes (COS) 7 cells revealed preferential silencing of Gα_q_ signaling in a cellular context‐dependent manner.^19^ Along the activation pathway, BIM allows GDP release, but prevents GTP entry, thus trapping Gα_q_ in the nucleotide‐free, empty pocket conformation.^19^ The dimeric BIM‐46187 has often been experimentally employed,^20^ particularly in order to take advantage of its preferred Gα_q_ inhibition.^21^

Despite the apparent usability of BIM‐46187, systematic medicinal chemistry approaches are lacking. Just one recent study on BIM fragments demonstrated that distinct structural reductions were incompatible with a Gα_q_ inhibitory activity.^22^ Therefore, we introduced several points of diversity into the full‐size BIM structure and envisaged a combinatorial approach towards an extended series of derivatives of BIM‐46187 (**1**). We devised a variety of BIM dimer structural analogs, which are expected to exhibit similar redox behavior. Hence, their activity in a cellular environment should be comparable. Moreover, the thiol function of the BIM monomer was exchanged for other groups or converted to prodrug forms. This series of designed compounds, none of which bearing a free thiol group, was synthesized by means of combinatorial chemistry and subjected to biological investigations on Gα_q_ protein inhibition. We attempted to draw structure–activity relationships, define the BIM pharmacophore and explore the chemical space of Gα_q_ inhibitors of the BIM chemotype.

## Results and Discussion

### Chemistry

**Scheme 2:**
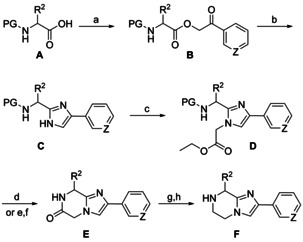
Synthesis of 5,6,7,8‐tetrahydroimidazo[1,2‐*a*]pyrazines **F**. Reagents and conditions: (a) 2‐bromoacetophenone (Z=CH) or 3‐(bromoacetyl)pyridine hydrobromide (Z=N), K_2_CO_3_, abs. DMF, rt, 4 h, 66–98 %; (b) NH_4_OAc, abs. toluene, reflux, 3 h, 69–91 %; (c) ethyl bromoacetate, Cs_2_CO_3_, abs. DMF, rt, 3.5 h, 60–96 %; (d) Pd/C, H_2_ (1 atm), abs. MeOH, rt, 24 h (PG=Cbz), 18–99 %; (e) TFA, abs. CH_2_Cl_2_, rt, 2 h; (f) Et_3_N_,_ CH_2_Cl_2_, rt, 2 h (PG=Boc); (g) BH_3_×THF, abs. THF, 90 °C, 48 h, Ar; (h) Pd/C, abs. MeOH, rt, 16 h, Ar; 53–93 %. PG=protecting group.

The structure of the BIM monomer can be rationalized as a heterocyclized dipeptide emerged from l‐cysteine and l‐cyclohexylalanine. The *C*‐terminal carboxyl group is replaced by a 4‐phenyl‐substituted imidazole in which the *N*‐1 nitrogen is bridged to the peptidic *N* atom via an ethylene linker, resulting in a 5,6,7,8‐tetrahydroimidazo[1,2‐*a*]pyrazine core. This scaffold enables a combinatorial approach by either modifying the heterobicyclic structure or introducing various substituents at different points of diversity. The synthesis (Scheme 2) started with the esterification of different d‐ or l‐configured, *N*‐protected amino acids **A** with either phenacyl bromide or 3‐(bromoacetyl)pyridine. The resulting α‐acyloxy ketones **B** were subjected to a Davidson‐type heterocondensation upon treatment with ammonia in refluxing toluene. 2,4‐Disubstituted imidazoles **C** were reacted with ethyl bromoacetate; the regioselectivity of this alkylation to occur at *N*‐1 has been recently confirmed by X‐ray crystallography.^22^ Cbz‐protected intermediates **D**, after hydrogenolytic deprotection, underwent an instantaneous entropy‐driven lactamization to **E**. The Boc protecting group of other representatives of type **D** was removed under acidic conditions and ring closure to **E** occurred at room temperature upon deprotonation of the ammonium group. The further conversion was accomplished by a borane‐promoted reduction, resulting in relatively stable amine‐borane adducts, which could not completely be decomplexed with methanol alone, but in the presence of palladium,^23^ leading to the desired set of 10 secondary amines **F**.

**Scheme 3:**
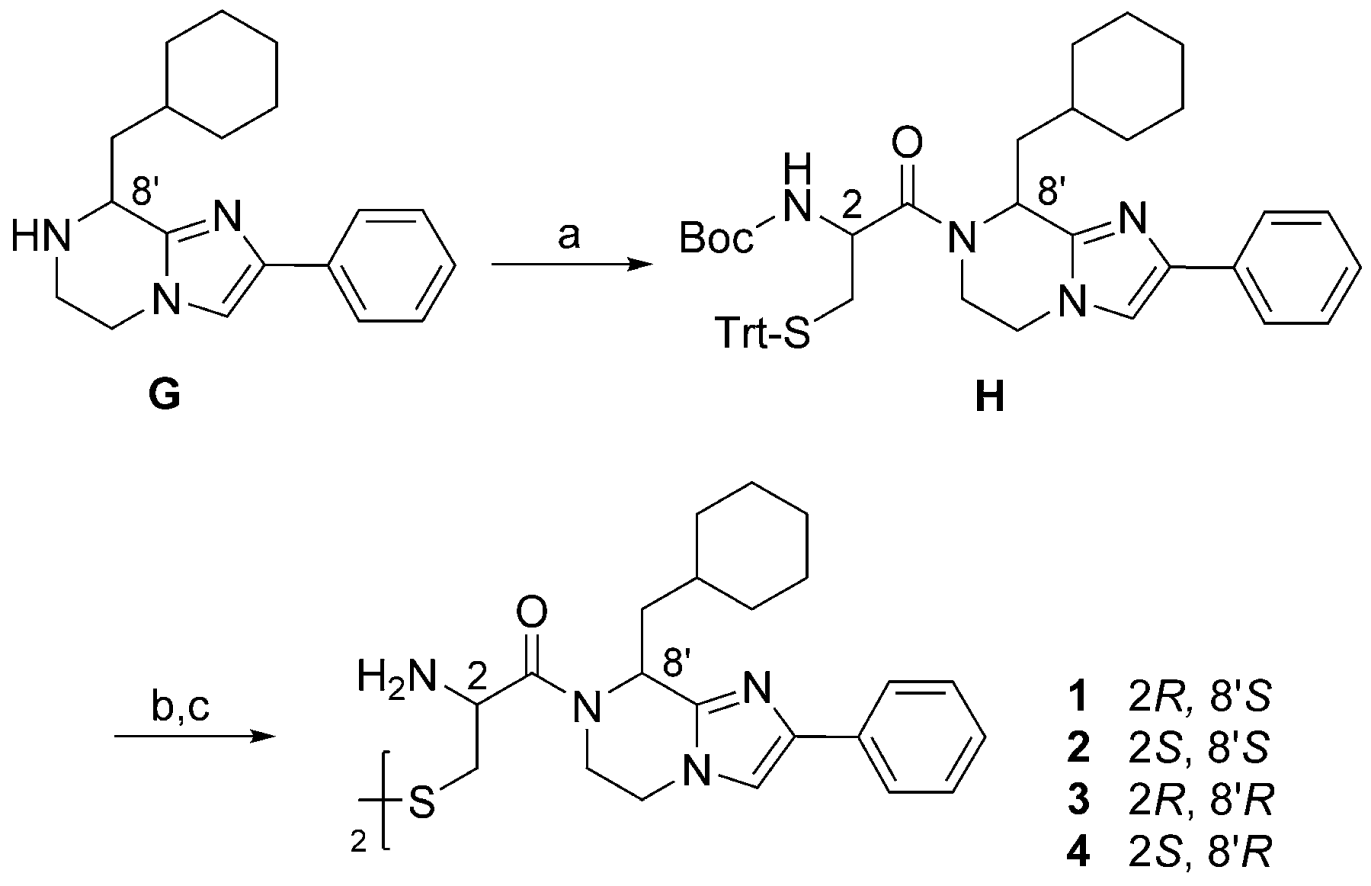
Synthesis of BIM‐46187 (**1**) and its stereoisomers (**2**–**4**). Reagents and conditions: (a) Boc‐l‐Cys(Trt)‐OH or Boc‐d‐Cys(Trt)‐OH, DCC, DIPEA, abs. CH_2_Cl_2_, rt, 48.5 h, Ar, 63–79 %; (b) TFA, (*i*Pr)_3_SiH, abs. CH_2_Cl_2_, rt, 18 h, Ar; (c) I_2_, MeOH, H_2_O, rt, 2 h, 44–72 %.

Two representatives of this set, that is, the (*S*)‐ and (*R*)‐configured cyclohexylalanine derivatives **G** (Scheme 3) were employed for the synthesis of BIM‐46187 (**1**),^19^ its enantiomer **4** and its diastereomers **2** and **3**. The carbodiimide‐mediated amide coupling introduced the cysteine unit in **H**. Both the *tert*‐butyloxycarbonyl and trityl group were removed contemporaneously under acidic conditions in the presence of excess triisopropylsilane, a hydride donor, acting as a scavenger of the trityl, and also the *tert*‐butyl cation.^24^ The intermediate thiols were oxidized with iodine, and stereoisomers **1**–**4** were purified by column chromatography, then dissolved in ethyl acetate and precipitated as hydrochlorides.

**Scheme 4:**
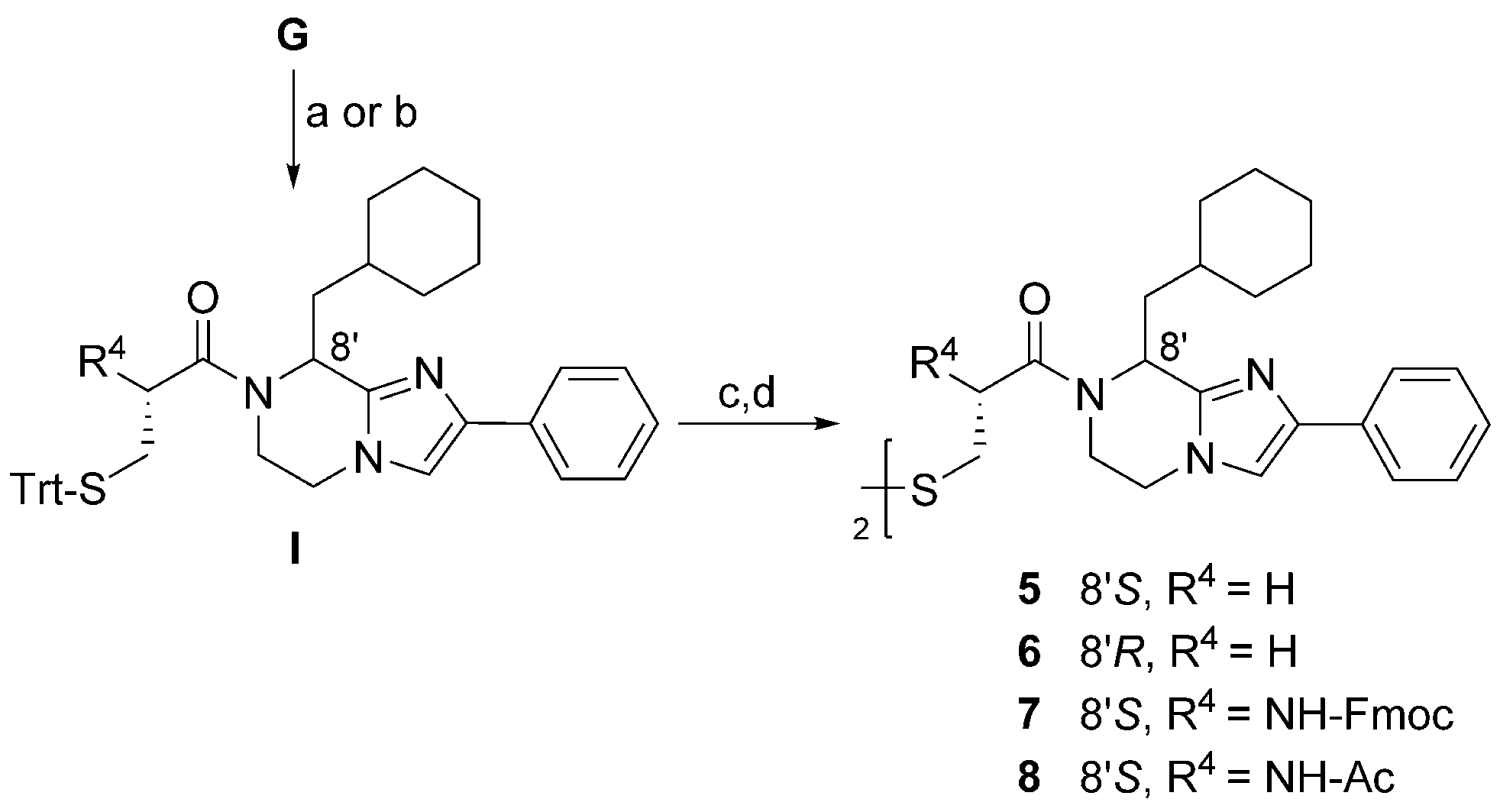
Synthesis of BIM analogs **5**–**8**. Reagents and conditions: (a) 3‐(tritylthio)propanoic acid or Fmoc‐l‐Cys(Trt)‐OH, DCC, DIPEA, abs. CH_2_Cl_2_, rt, 48.5 h, Ar (R^4^=H or NH‐Fmoc); (b) Ac‐l‐Cys(Trt)‐OH, HATU, DIPEA, abs. DMF, rt, 48.5 h, Ar (R^4^=NH‐Ac), 29–68 %; (c) TFA, (*i*Pr)_3_SiH, abs. CH_2_Cl_2_, rt, 18 h, Ar; (d) I_2_, MeOH, H_2_O, rt, 2 h, 8–13 %.

The subseries **5**–**8** was prepared in order to examine the effect of the basic primary amino group on the bioactivity of the analogs. The NH_2_ group was either removed or acetylated, or equipped with a bulky carbamoyl substituent. The first three intermediates **I** (Scheme 4) were synthesized by means of DCC, while *N*‐acetyl‐*S*‐trityl‐l‐cysteine was introduced with HATU. Prior to oxidation, the detritylation to **5** and **6** and the orthogonal deprotection to **7** and **8** were carried out as before.

**Scheme 5:**
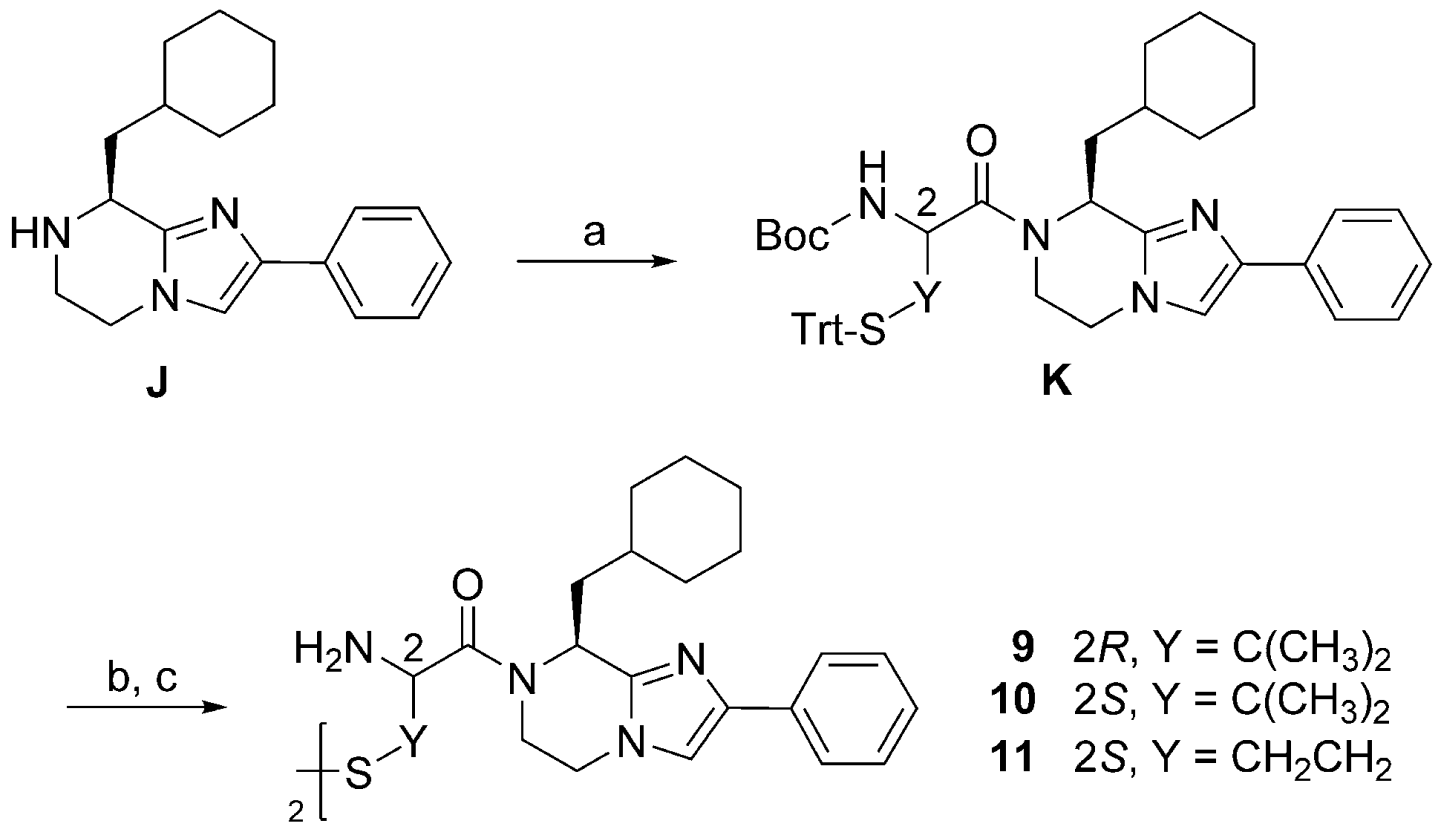
Synthesis of BIM analogs **9**–**11**. Reagents and conditions: (a) Boc‐l‐Pen(Trt)‐OH or Boc‐d‐Pen(Trt)‐OH or Boc‐l‐Hcy(Trt)‐OH, DCC, DIPEA, abs. CH_2_Cl_2_, rt, 48.5 h, Ar, 35–41 %; (b) TFA, (*i*Pr)_3_SiH, abs. CH_2_Cl_2_, rt, 18 h, Ar; (c) I_2_, MeOH, H_2_O, rt, 2 h, 5–38 %.

Moreover, products with a modified cystine methylene unit were devised whose syntheses are depicted in Scheme 5. The dimeric penicillamine (H‐Pen‐OH) derivatives **9** and **10** are dimethylated analogs of **1** and **2**, respectively. In compound **10**, the structure of the drug d‐penicillamine was embedded. We employed homocysteine (H‐Hcy‐OH) in its double‐protected form for the methylene‐ethylene replacement to provide the homocystine derivative **11**. Its absolute geometry did not change, but, owing to CIP priority rules, the configuration at the C‐2 carbon is (*S*).

**Scheme 6:**
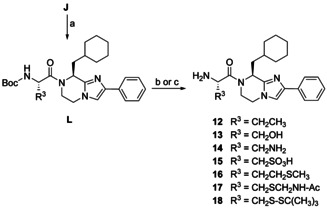
Synthesis of BIM analogs **12**–**18**. Reagents and conditions: (a) Boc‐l‐Abu‐OH or Boc‐l‐Ser(Trt)‐OH or Boc‐l‐Dap(Boc)‐OH or Boc‐l‐cysteic acid or Boc‐l‐Met‐OH or Boc‐l‐Cys(Acm)‐OH or Boc‐l‐Cys(S*t*Bu)‐OH, DCC, DIPEA, abs. CH_2_Cl_2_, rt, 48.5 h, Ar, 21–82 %; (b) TFA, abs. CH_2_Cl_2_, rt, 2 h (to **12**, **14**–**18**), 5–95 %; (c) TFA, (*i*Pr)_3_SiH, abs. CH_2_Cl_2_, rt, 18 h (to **13**), 66 %.

The starting compound **J** was also used to prepare intermediates **L** which were subsequently Boc‐deprotected to the final compounds **12**–**18** (Scheme 6). To obtain **13** from the Boc‐Trt‐protected precursor, we added triisopropylsilane to facilitate the liberation of the alcoholic group. In the other cases, the same acidic conditions were applied in the final step. The route to the 1,2‐diaminopropionate (H‐Dap‐OH) derivative **14** involved the β‐amino protection of Boc‐l‐Dap‐OH, followed by the coupling step and the simultaneous release of both amino groups. To furnish **15**, l‐cysteic acid was initially *N*‐Boc‐protected, then subjected to coupling conditions in which the free sulfonyl group remained unaffected, and finally deprotected. Products **12**–**16** can be considered as analogs of the BIM monomer, in which the thiol function is exchanged for a methyl, hydroxy, amino, sulfo, and methylthiomethyl group. Analogs **17** and **18** are BIM‐prodrugs; the acetamidomethyl (Acm) group of the former might undergo intracellular hydrolytic deacetylation and a subsequent decay to the BIM monomer; the latter is expected to undergo fragmentation in the reducing environment of the cell to release the corresponding thiol.

**Scheme 7:**
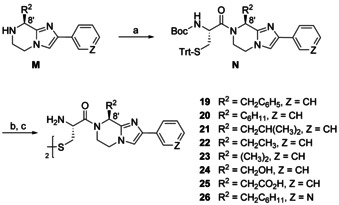
Synthesis of BIM analogs **19**–**26**. Reagents and conditions: (a) Boc‐l‐Cys(Trt)‐OH, DCC, DIPEA, abs. CH_2_Cl_2_, rt, 48.5 h, Ar, 40–80 %; (b) TFA, (*i*Pr)_3_SiH, abs. CH_2_Cl_2_, rt, 18 h, Ar; (c) I_2_, MeOH, H_2_O, rt, 2 h, 4–38 %.

The synthetic route in Scheme 7 followed the aforementioned coupling‐deprotection‐oxidation sequence. Compounds **19**–**26** differ with respect to two points of diversity, the amino acid‐derived residue R^2^ and the aromatic substituent, phenyl or 3‐pyridyl, at the imidazole. The structural variability was already inherent in the substitution pattern of 8 secondary amines **M**. The achiral precursor for **23** bears two methyl groups at position 8′, the other intermediates **M** were obtained from (*S*)‐configured amino acids, whereupon (*S*)‐serine led to the (*R*)‐configured final product **24**.

**Scheme 8:**
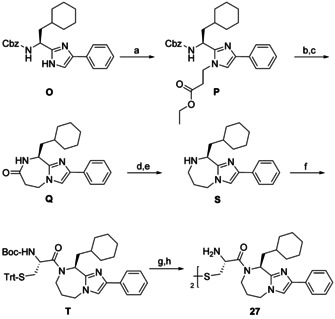
Synthesis of the imidazo[1,2‐*a*][1,4]diazepine derivative **27**. Reagents and conditions: (a) ethyl 3‐bromopropionate, Cs_2_CO_3_, abs. DMF, rt, 3.5 h, 88 %; (b) Pd/C, H_2_ (1 atm), abs. MeOH, rt, 24 h; (c) abs. EtOAc, TsOH, reflux, 16 h, 55 %; (d) BH_3_×THF, abs. THF, 90 °C, 48 h, Ar; (e) Pd/C, abs. MeOH, rt, 16 h, Ar, 51 %; (f) Boc‐l‐Cys(Trt)‐OH, DCC, DIPEA, abs. CH_2_Cl_2_, rt, 48.5 h, Ar, 79 %; (g) TFA, (*i*Pr)_3_SiH, abs. CH_2_Cl_2_, rt, 18 h, Ar; (h) I_2_, MeOH, H_2_O, rt, 2 h, 12 %.

It was intended to expand the fused 6‐membered ring in **1** to a 1,4‐diazepane substructure in **27** (Scheme 8). Somewhat different reaction conditions were needed to obtain compound **Q**, since the increased ring strain of the 7‐membered ring and the reduced electrophilicity of the ester carbonyl in **P**, when compared with **D** (Scheme 2), exacerbated the lactamization. The propylene linker was formed by the borane‐promoted reduction of **Q**. Subsequent coupling, deprotection and oxidation yielded the envisaged product **27**, in which the relative orientation of the phenyl group and the half‐cystinyl residue are slightly shifted.

**Scheme 9:**
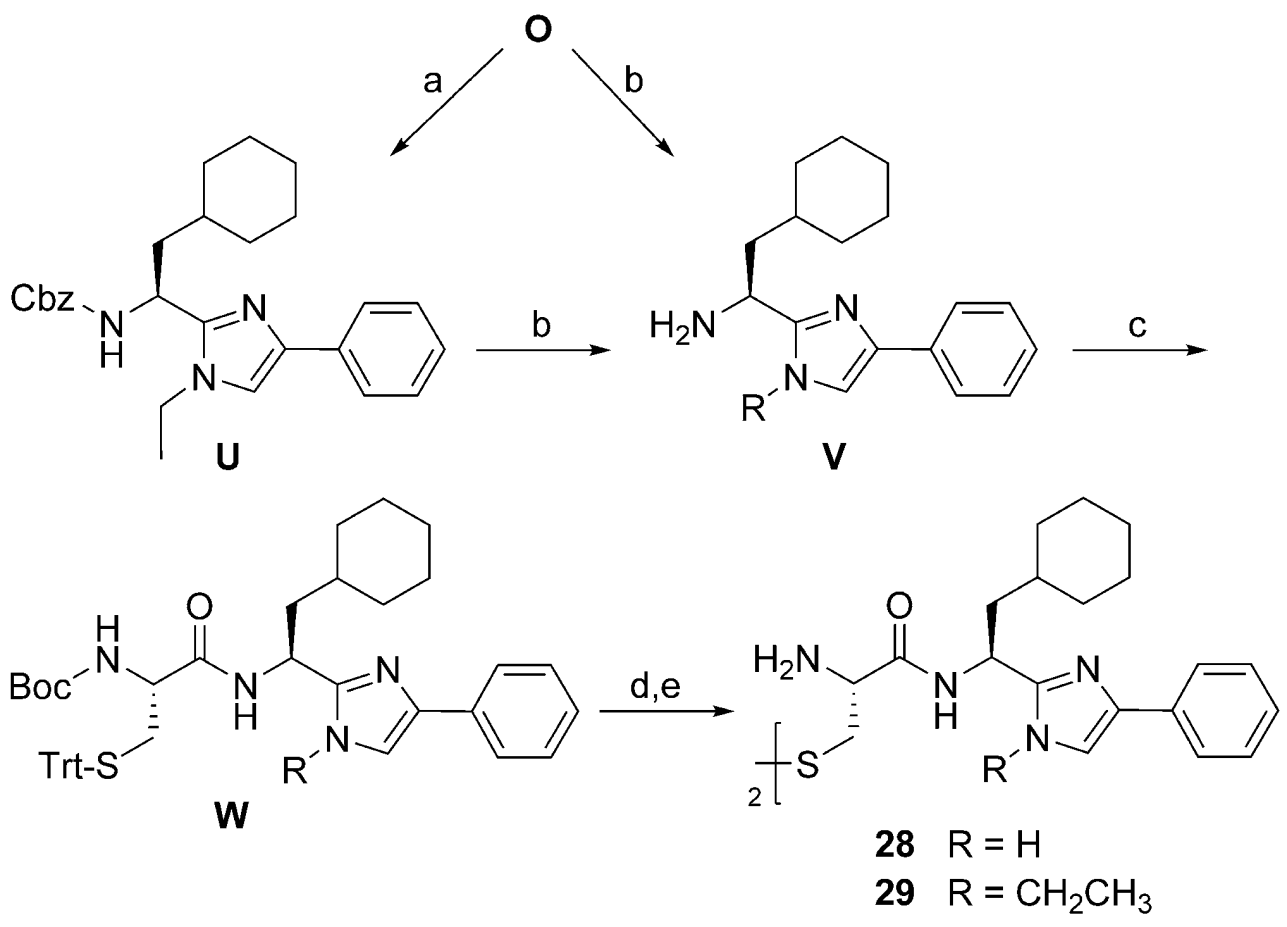
Synthesis of monocyclic BIM analogs **28** and **29**. Reagents and conditions: (a) bromoethane, Cs_2_CO_3_, abs. DMF, rt, 3.5 h, 88 %; (b) Pd/C, H_2_ (1 atm), abs. MeOH, rt, 24 h, 94–95 %; (c) Boc‐l‐Cys(Trt)‐OH, DCC, DIPEA, abs. CH_2_Cl_2_, rt, 48.5 h, Ar, 80–94 %; (d) TFA, (*i*Pr)_3_SiH, abs. CH_2_Cl_2_, rt, 18 h, Ar; (e) I_2_, MeOH, H_2_O, rt, 2 h, 12–32 %.

Analogs with a higher degree of conformational heterogeneity were prepared as shown in Scheme 9. We either removed the ethylene linker of **1** in the target compound **28** or cut one N−C bond in **29**. These two monocyclic imidazole derivatives possess a higher flexibility owing to the free rotation about the *C*α‐*C* and *C*α‐*N* cyclohexylalanine backbone bonds. As in Scheme 8, the synthesis started with compound **O** which was ethylated at position *N*‐1 to intermediate **U**. Both **O** and **U** were subjected to hydrogenolytic *N*‐deprotection and the resulting two primary amines **V** were converted via intermediates **W** to products **28** and **29**.

### G protein inhibition

**Figure 1:**
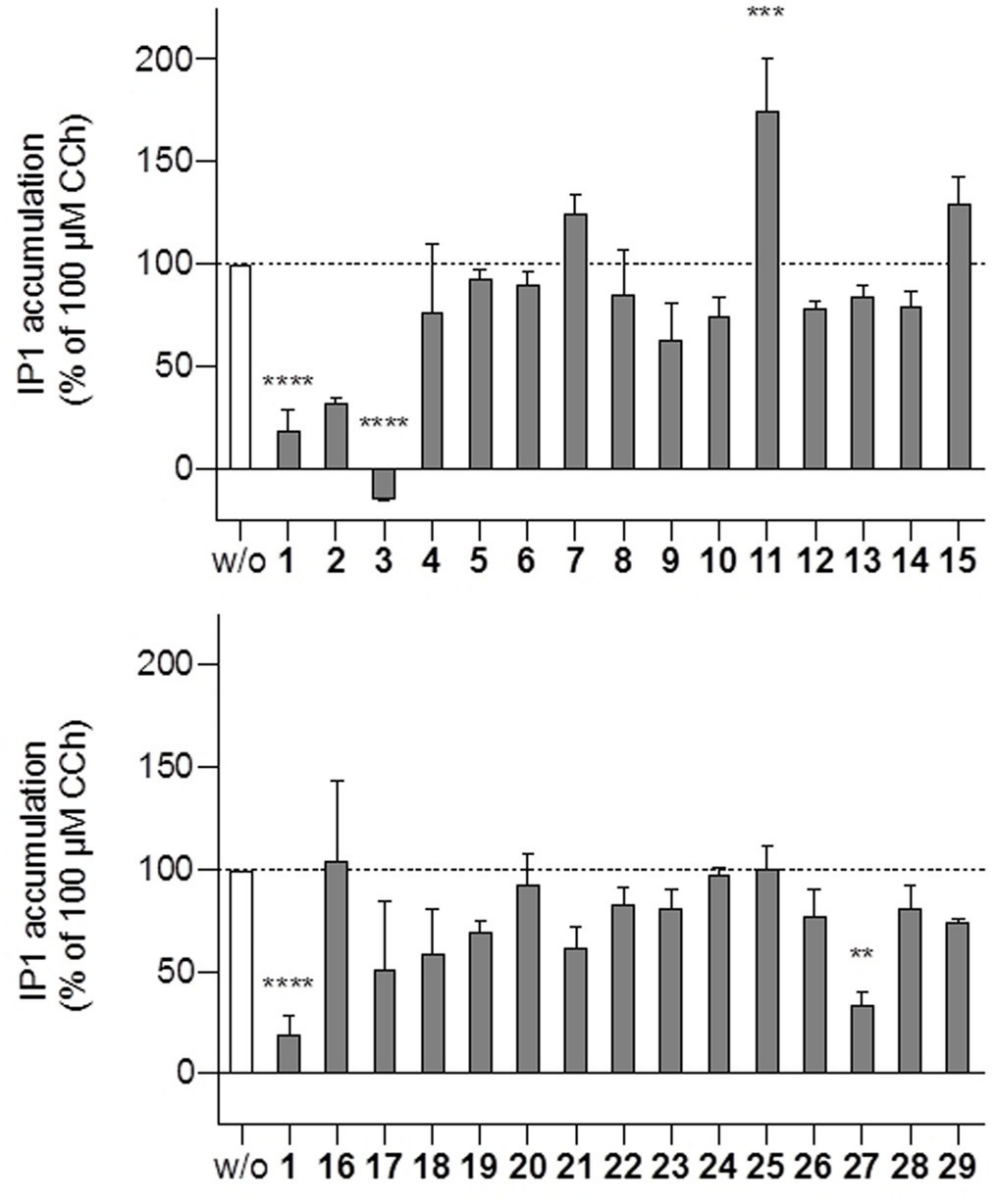
Gα_q_‐dependent IP1 formation in CCh‐stimulated HEK293 cells pretreated with 100 μm of BIM‐46187 (**1**) and its analogs **2**–**15** (top) and **16**–**29** (bottom). Data are means ± s.e.m. of two independent experiments. **=P<0.01, ***=P<0.001, ****=P<0.0001 compared to w/o using Dunnett's multiple comparisons after one‐way ANOVA.

We used carbachol (CCh), an agonist of muscarinic acetylcholine receptors (mAChRs) to induce G protein signaling in HEK293 cells. The M3 mAChR is expressed in HEK293 wild‐type (wt) cells and couples to Gα_q_ and Gα_12_ proteins thereby stimulating phospholipases (PLs) C and D in a pertussis toxin‐insensitive manner. In particular, the molecular interaction of Gα_q_ and PLC‐β effectuates the cleavage of a membrane phospholipid, that is, PIP2 into DAG and IP3, both acting as important second messengers. IP3 is further hydrolyzed through sequential dephosphorylation to its downstream metabolites *myo*‐inositol 1,4‐bisphosphate (IP2) and IP1. The assay which we have employed takes advantages of the inhibition of IP1 degradation by lithium chloride, allowing IP1 to accumulate in the cell, where it can be quantified as a substitute for IP3 and a measure of Gα_q_ inhibition. IP1 was determined in a competitive immunoassay after 2 hours incubation of test compounds (for structures, see Table 1) in a concentration of 100 μm followed by 35 min CCh stimulation. The results are shown in Figure 1.

**Table 1:** BIM analogs prepared.

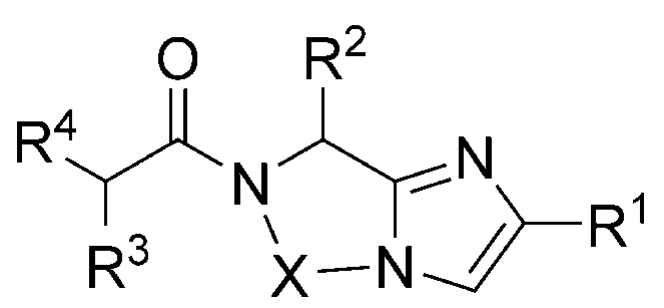					
Compound	R^1^	R^2^	R^3^	R^4^	X
**1**	C_6_H_5_	(*S*)‐CH_2_C_6_H_11_	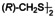	NH_2_	CH_2_CH_2_
**2**	C_6_H_5_	(*S*)‐CH_2_C_6_H_11_	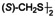	NH_2_	CH_2_CH_2_
**3**	C_6_H_5_	(*R*)‐CH_2_C_6_H_11_	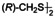	NH_2_	CH_2_CH_2_
**4**	C_6_H_5_	(*R*)‐CH_2_C_6_H_11_	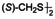	NH_2_	CH_2_CH_2_
**5**	C_6_H_5_	(*S*)‐CH_2_C_6_H_11_	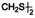	H	CH_2_CH_2_
**6**	C_6_H_5_	(*R*)‐CH_2_C_6_H_11_	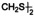	H	CH_2_CH_2_
**7**	C_6_H_5_	(*S*)‐CH_2_C_6_H_11_	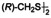	NH‐Fmoc	CH_2_CH_2_
**8**	C_6_H_5_	(*S*)‐CH_2_C_6_H_11_	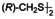	NH‐Ac	CH_2_CH_2_
**9**	C_6_H_5_	(*S*)‐CH_2_C_6_H_11_	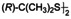	NH_2_	CH_2_CH_2_
**10**	C_6_H_5_	(*S*)‐CH_2_C_6_H_11_	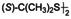	NH_2_	CH_2_CH_2_
**11**	C_6_H_5_	(*S*)‐CH_2_C_6_H_11_	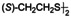	NH_2_	CH_2_CH_2_
**12**	C_6_H_5_	(*S*)‐CH_2_C_6_H_11_	(*S*)‐CH_2_CH_3_	NH_2_	CH_2_CH_2_
**13**	C_6_H_5_	(*S*)‐CH_2_C_6_H_11_	(*S*)‐CH_2_OH	NH_2_	CH_2_CH_2_
**14**	C_6_H_5_	(*S*)‐CH_2_C_6_H_11_	(*S*)‐CH_2_NH_2_	NH_2_	CH_2_CH_2_
**15**	C_6_H_5_	(*S*)‐CH_2_C_6_H_11_	(*R*)‐CH_2_SO_3_H	NH_2_	CH_2_CH_2_
**16**	C_6_H_5_	(*S*)‐CH_2_C_6_H_11_	(*S*)‐CH_2_CH_2_SCH_3_	NH_2_	CH_2_CH_2_
**17**	C_6_H_5_	(*S*)‐CH_2_C_6_H_11_	(*R*)‐CH_2_SCH_2_NH‐Ac	NH_2_	CH_2_CH_2_
**18**	C_6_H_5_	(*S*)‐CH_2_C_6_H_11_	(*R*)‐CH_2_S‐SC(CH_3_)_3_	NH_2_	CH_2_CH_2_
**19**	C_6_H_5_	(*S*)‐CH_2_C_6_H_5_	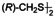	NH_2_	CH_2_CH_2_
**20**	C_6_H_5_	(*S*)‐C_6_H_11_	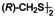	NH_2_	CH_2_CH_2_
**21**	C_6_H_5_	(*S*)‐CH_2_CH(CH_3_)_2_	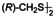	NH_2_	CH_2_CH_2_
**22**	C_6_H_5_	(*S*)‐CH_2_CH_3_	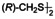	NH_2_	CH_2_CH_2_
**23**	C_6_H_5_	(CH_3_)_2_	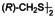	NH_2_	CH_2_CH_2_
**24**	C_6_H_5_	(*R*)‐CH_2_OH	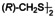	NH_2_	CH_2_CH_2_
**25**	C_6_H_5_	(*S*)‐CH_2_CO_2_H	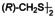	NH_2_	CH_2_CH_2_
**26**	3‐pyridyl	(*S*)‐CH_2_C_6_H_11_	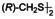	NH_2_	CH_2_CH_2_
**27**	C_6_H_5_	(*S*)‐CH_2_C_6_H_11_	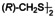	NH_2_	CH_2_CH_2_CH_2_
**28**	C_6_H_5_	(*S*)‐CH_2_C_6_H_11_	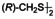	NH_2_	H, H
**29**	C_6_H_5_	(*S*)‐CH_2_C_6_H_11_	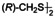	NH_2_	H, CH_2_CH_3_

In accordance with previous investigations,^19^ the BIM dimer (**1**) showed a strong inhibition of the Gα_q_‐dependent IP1 formation and exhibited an IC_50_ value of 31.9 μm (see the Supporting Information, Figure S1). The results of the present study revealed, for the first time, the effect of the stereochemistry of isomers on the biological activity. The (*R*)‐configuration of the cystine substructure was required for Gα_q_ inhibition, but inversion of the cyclohexylalanine configuration was tolerated. Hence, out of the four stereoisomers, only **1** and **3** (IC_50_=56.3 μm; see the Supporting Information, Figure S1) were potent inhibitors. We assume that **1**–**4** with highly similar physicochemical properties share the cellular uptake capability and specific intracellular drug‐protein interactions account for different IP1 accumulation. The positively charged terminal ammonium group was essential for Gα_q_ inhibitory activity, since neither compounds **5** and **6**, the desamino derivatives of **1** and **3**, nor compounds **7** and **8**, the *N*‐protected analogs of **1**, were active. Subtle changes in the cystine side chain of **1** caused inactivity. This was the case, when it was equipped with two methyl groups (in **9**). In contrast, elongation of the cystine side chain of **1** by one methylene group (in **11**) led to an unforeseen enhancement of IP1, both in CCh‐stimulated cells (Figure 1) and in HEK293 cells without CCh pretreatment (see the Supporting Information, Figure S2). The increased level of cellular inositol phosphates caused by **11** in a CCh‐independent manner potentially indicates a direct activation of Gα_q_ or its downstream signaling pathway.

Compound **10**, with the substructure of the drug d‐penicillamine incorporated, was also inactive. IP1 accumulation was maintained after treatment with compounds **12**–**16**. In contrast to **1** and **3**, compounds **12**–**16** are incapable of exhibiting redox reactivity. Under the assumption that BIM dimers undergo an intracellular reductive cleavage, the thus formed thiol group might be requisite for further transformations or direct Gα_q_ inhibition. Such a mode of action is not possible in case of **12**–**16**. Compounds **17** and **18** did not serve as successful prodrugs for the BIM monomer. Since **18** represents a disulfane, albeit of unsymmetrical structure, we expected a bioactivation resulting in an efficacy comparable to that of **1**. However, Gα_q_ inhibition was not observed.

We next examined whether the desired bioactivity could be achieved in the course of structural modifications at the cyclohexylmethyl substructure (in compounds **19**–**25**). For example, we exchanged the cyclohexane chair for the flat benzene ring (in **19**), removed a methylene (in **20**) or propylene (in **21**) or pentylene unit (in **22**), or introduced polar groups (in **24** and **25**). Our data indicated that the presence of the cyclohexylmethyl moiety (**1** versus **19**–**25**) as well as the phenyl group (**1** versus **26**) was beneficial. A further active inhibitor of the Gα_q_ protein was achieved when we implemented the ring expansion of **1** as realized in the propylene‐containing compound **27** (IC_50_=50.2 μm; see the Supporting Information, Figure S1). An increase of conformational flexibility in case of the monocyclic analogs **28** and **29** turned out to be disadvantageous. Taken together, the following main structural requirements for active BIM analogs were clarified (i) the redox reactive cystine/cysteine substructure, (ii) the *N*‐terminal basic amino group, (iii) the cyclohexylalanine moiety, (iv) a bicyclic skeleton. These considerations provide a rather limited scope for the future design of related Gα_q_ inhibitors.

The extraordinary potency of YM‐254890 and FR900359 (IC_50_ values of 1.06 μm and 0.77 μm, respectively, see the Supporting Information, Figure S1) was not attained with the BIM analogs of this study. Furthermore, their mode of action is also distinct. While BIM‐46187 (**1**) and its monomer inhibit Gα_q_ signaling by precluding GTP entry while permitting GDP exit,^19^ YM‐254890 and FR900359 function as inhibitors of GDP dissociation, that is, stabilize Gα_q_ in its inactive GDP‐bound state.^5^, ^6^ Therefore, YM‐254890 and FR900359, but not BIM molecules, specifically disrupt high‐affinity agonist binding of Gα_q_‐selective GPCRs, as they impair formation of the nucleotide‐free ‚empty pocket state’, the G protein species required to stabilize this high‐affinity interaction.

### Cellular toxicity

**Figure 2:**
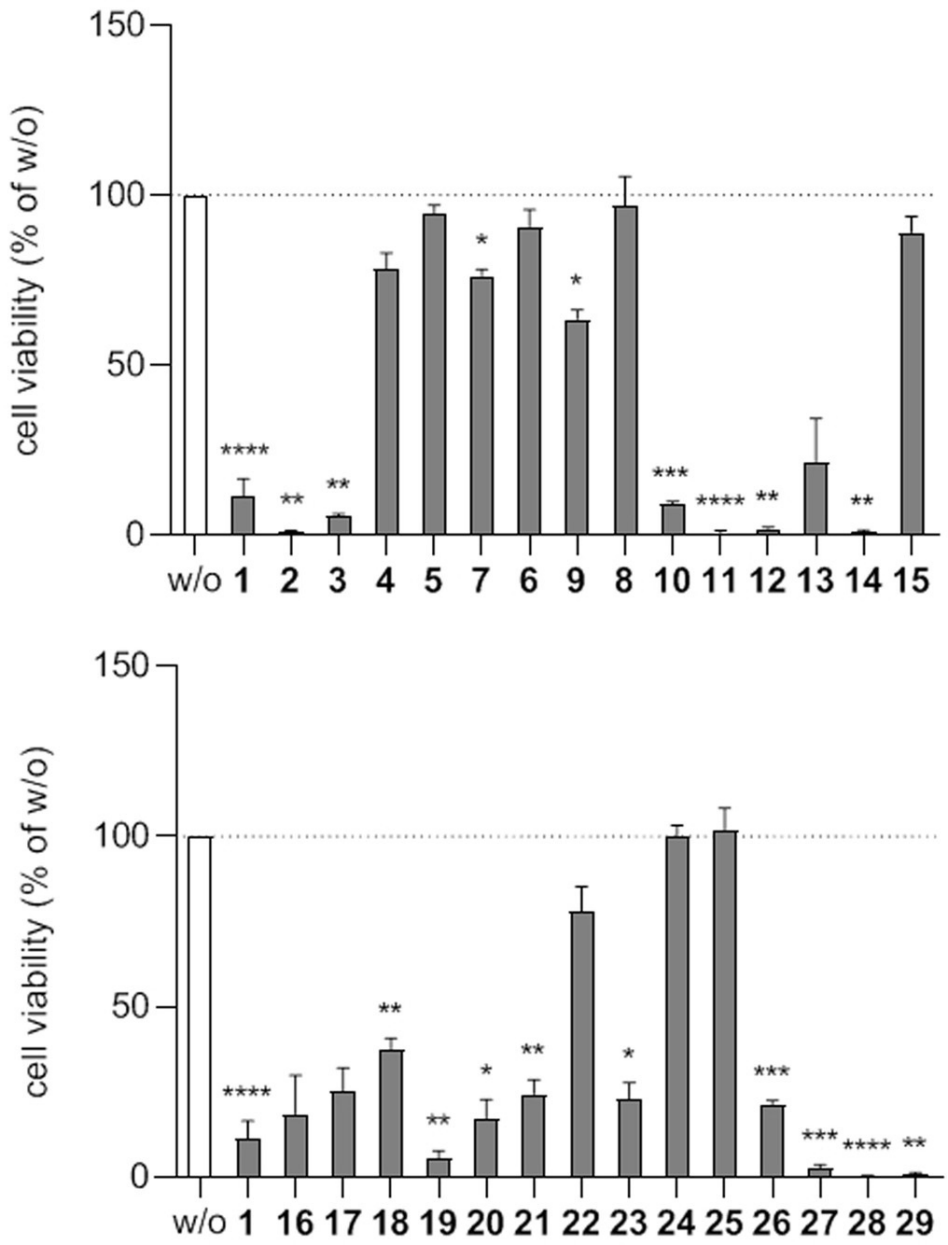
Viability of HEK293 cells treated with 100 μm BIM‐46187 (**1**) and its analogs **2**–**15** (top) and **16**–**29** (bottom). Data are presented as means ±s.e.m. of two independent experiments. *=P<0.05, **=P<0.01, ***=P<0.001, ****=P<0.0001 compared to w/o using Dunnett's multiple comparisons after one‐way ANOVA.

The toxicity of all compounds was determined by applying a CellTiter‐Blue viability assay (Figure 2). It is well‐established that Gα_q_ inhibition *per se* is not linked to cellular toxicity.^6^ This finding offered the opportunity to separate efficient Gα_q_ inhibition from adverse toxic effects. On the one hand, an indication for the possible independency of both properties is apparent from the distinct cellular toxicity of several BIM analogs which did not inhibit Gα_q_ (e.g. **10**–**12**, **14**, **19**, **28** and **29**; Figure 1 and Figure 2). On the other hand, significant inhibition was only observed for toxic BIM compounds (**1**, **3** and **27**; Figure 1 and Figure 2). Among the derivatives without a free *N*‐terminal amino group, some were not toxic (**5**, **6** and **8**, Figure 2). Moreover, representatives bearing polar groups as part of R^2^ (**24** and **25**) or the acidic sulfonate moiety in R^3^ (**15**) did not affect cell viability either. Hence, a zwitterionic structure (**15**, **25**) might prevent cellular toxicity, an assumption to be considered in future attempts towards molecular separation of desired (Gα_q_ inhibition) and unwanted features (cellular toxicity) of BIM analogs.

To investigate whether BIM type inhibitors require Gα_q_ to exert their cell cytotoxic effects, we took advantage of HEK293 cells, depleted by CRISPR/Cas9 of Gα_q_ and Gα_11_ subunits of heterotrimeric G proteins. Cells treated with the BIM dimer (**1**) were subjected to non‐invasive live cell imaging. We studied the response of wt and Gα_q/11_ KO HEK293 cells to a 30 min long application of **1** (30 μm) versus DMSO (1:3000) as control. Within 30 min, the cell shape changed, as cells of both cell lines strongly rounded up (Supporting Information, Figure S3A). From these results we concluded that BIM‐induced cell rounding does not require heterotrimeric Gα_q_ family proteins. Since the cytoskeleton is an essential cellular component responsible for the maintenance of the morphology and several functions of the cell, we reasoned that cytoskeletal changes might be the origin for the observed toxicity. To elucidate the potential mechanisms underlying the compound‐induced changes of cell shape, the cytoskeleton was analyzed. For this purpose, we stained against microtubules and intermediate filaments with a direct fluorescent antibody against β‐tubulin, and through indirect immunofluorescence against vimentin, respectively. To visualize microfilaments, we used rhodamine phalloidin as a fluorescent probe for F‐actin. The microtubular network of wt and Gα_q/11_ KO HEK293 cells was not affected by **1** (Supporting Information, Figure S3B), whereas both wt and Gα_q/11_ KO cells showed a preferential perinuclear accumulation of vimentin and intermediate filaments in response to treatment with **1** (Supporting Information, Figure S3C). The most prominent changes could be observed in phalloidin staining, as both DMSO‐treated cell lines displayed prominent F‐actin stress fibers with small protrusions. In clear contrast, compound **1** induced strong rounding up of the cells (Supporting Information, Figure S3D) and this was accompanied by predominant cortical actin re‐localization, whereas almost neither stress fibers nor actin surrounding the nucleus of the cells occurred. These data demonstrate that the BIM dimer (**1**) has prominent effects on cell shape and that these are due to its action on the cytoskeletal components. Cytoskeletal changes in transformed cells are involved in cell proliferating and metastatic capabilities.^25^ Noteworthy, our data on the Gα_q/11_‐independent influence of the BIM dimer (**1**) on components of the cytoskeleton illustrates an additional mechanism by which BIM molecules act as agents against tumor cells.

## Conclusions

These investigations were carried out to explore the chemical space of Gα_q_ inhibitors with a heterocyclized dipeptide structure. Although the structure of BIM offers numerous opportunities for combinatorial modifications and several have been realized in this study, only a narrow frame for tolerable structural alterations was recognized. Hence, we defined substructures required for Gα_q_ inhibition and identified two novel bioactive compounds which possess high similarity with the BIM dimer. Redox reactivity was shown to be requisite for cellular activity indicating the involvement of intracellular thiols, in particular glutathione, for bioactivation of BIM‐type disulfanes, probably catalyzed by the thioredoxin‐glutaredoxin system.^26^ Two cysteine residues, Cys330 or Cys144, are conserved in all Gα_q_ proteins and might constitute potential binding sites for **1**, consistent with the intradomain movement within the target protein.^19^ Future studies are needed to ascertain whether a covalent interaction of Gα_q_ with BIM‐type homodimers, their reduced monomers or BIM‐glutathione heterodimers takes place. Herein we discovered that the prototypical Gα_q_ inhibitor **1** affects the structural cytoskeletal dynamics of treated cells. Moreover, our finding provides evidence that BIM‐type inhibitors display their cellular toxicity in a Gα_q_‐independent manner. The usability of compounds targeting cytoskeletal dynamics has been considered to be limited because they affect a variety of processes in both cancer and normal cells. However, there are microtubule‐binding compounds that became valuable drugs for cancer treatment, in particular vinca domain‐binding agents, for example, vincristine, and taxol domain‐binding agents, for example, paclitaxel.^25^ Compound **1** does not exhibit typical molecular features of cytoskeleton‐targeting compounds. Accordingly, future studies might be focused on structure–activity relationships with respect to the induction of cytoskeletal changes by BIM‐type Gα_q_ inhibitors.

## Experimental Section

Detailed descriptions of synthetic procedures and cellular experiments as well as analytical properties of all prepared compounds and biological data are given in the Supporting Information.

## Conflict of interest

The authors declare no conflict of interest.

## Supporting information

As a service to our authors and readers, this journal provides supporting information supplied by the authors. Such materials are peer reviewed and may be re‐organized for online delivery, but are not copy‐edited or typeset. Technical support issues arising from supporting information (other than missing files) should be addressed to the authors.

Supplementary
