# Supplementary material for: Tetrahydroimidazo[1,2‐a]pyrazine Derivatives: Synthesis and Evaluation as Gαq‐Protein Ligands
Source: Chemistry. 2020 Sep 7;26(55):12615–23. doi: 10.1002/chem.202001446 (PMC7590114; doi:10.1002/chem.202001446)
Supplement: Supplementary file 1 — Supplementary [file CHEM-26-12615-s001.pdf]

Chemistry–A European Journal

Supporting Information

Tetrahydroimidazo[1,2-*a*]pyrazine Derivatives: Synthesis and Evaluation as Gα_q-Protein Ligands

Jim Küppers+,[a] Tobias Benkel+,[b, c] Suvi Annala,[b] Kenichi Kimura,[d] Lisa Reinelt,[a] Bernd K. Fleischmann,[d] Evi Kostenis,*[b] and Michael Gütschow*[a]

Supporting Information

Tetrahydroimidazo[1,2-*a*]pyrazine Derivatives: Synthesis and Evaluation As Gα_q-Protein Ligands

Jim Küppers, Tobias Benkel, Suvi Annala, Kenichi Kimura, Lisa Reinelt, Bernd K. Fleischmann, Evi Kostenis, and Michael Gütschow

Content

1. Cellular Biology S2

1.1. Experimental Procedures S2

1.2. Figures S4

Figure S1. G protein inhibition profiles of selected Gα_q inhibitory ligands S4

Figure S2. Effect of test compounds on CCh-independent IP1 formation S5

Figure S3. Live cell imaging data S6

2. Chemistry S8

2. 1. General Methods & Instruments S8

2. 2. Preparation of Compounds & Spectral Data S10

2.3. Chiral HPLC Analysis of Compounds 1, 2, 3, and 4 S57

3. Abbreviations S63

4. Author Contributions S63

5. References S64

1. Cellular Biology

1.1. Experimental Procedures

Inositol monophosphate (IP1) accumulation assay. HEK293 wt cells were obtained from the American Type Culture Collection (ATCC). HEK293 wt cells were cultured in DMEM (Invitrogen) containing 10% fetal bovine serum (PAN biotech, Germany), 100 U mL^{-1} penicillin and 100 mg mL^{-1} streptomycin (Invitrogen) in a humidified CO_2 incubator at 37 °C and 5% CO_2. The amount of intracellular IP1 was measured in a 384-well format using the homogenous time-resolved fluorescence (HTRF) -IP1 kit (Cisbio) as per manufacturer's instructions. The assay is based on the interaction of a fluorescent donor, terbium cryptate, and a second fluorescent label, *d*2, serving as acceptor. A cell suspension was dispensed with a density of 60,000 cells/well into 384-well plates (7 µL/well). After 15 min incubation at 37 °C, 3.5 µL stimulation buffer containing BIM-46174 (**1**) or a BIM-analog (**2**-**29**) was added and incubated for 2 h at 37 °C. Hereafter, 3.5 µL stimulation buffer containing 100 µM carbachol (CCh) was added. After further incubation at 37 °C for 35 min, IP1-*d*2 conjugate (3 µL) followed by terbium cryptate-labeled anti-IP1 antibody (3 µL) was added. Time-resolved fluorescence at 620 and 665 nm was measured with the Mithras LB 940 multimode reader after incubation at room temperature for 60 min, and the ratios of the signals were calculated as described previously.[1]

Colorimetric cell viability determination. Viability of HEK293 wt cells was assessed using a fluorimetric detection of resorufin (CellTiter-BlueTM cell viability assay, Promega). Specifically, cells were seeded (80 µL) at a density of 25,000 cells per well into black 96-well poly-D-lysine-coated plates with clear bottom and cultivated overnight. On the following day, 20 µL of BIM-46174 (**1**) or a BIM-analog (**2**-**29**) were added to the cells and incubated for 22 h, followed by the addition of 20 µL Cell-Titer-BlueTM reagent per well. After shaking for 10 s, the plates were further incubated for 2 h. Fluorescence (excitation 560 nm, emission 590 nm) was measured using a FlexStation 3 Benchtop Multimode Plate Reader. Data were expressed as percentage of cell viability relative to buffer treatment.

*Immunofluorescence stainings of cells treated with BIM-46187 (**1**).* HEK293 wt cells were cultured in DMEM supplemented with 10% fetal bovine serum and penicillin/streptomycin. Cells were grown on gelatine (0.1%) treated 24-well plates. Testing of different concentrations of **1** and different treatment times on HEK293 wt cells revealed that application of 30 µM of **1** for 30 min did induce shape changes, but the cells remained

attached. Therefore, either 30 µM of compound **1** or DMSO (1:3000) as control was applied for 30 min to wt and $G\alpha_{q/11}$ KO HEK293 cells. The corresponding cells were fixed with 4% paraformaldehyde in phosphate-buffered saline (PBS) and stained with primary antibody for vimentin (1:200, Merck) in 0.2% Triton X in PBS, supplemented with 5% donkey serum for 1.5 h at room temperature. The primary antibody was visualized by a secondary antibody conjugated to Cy3 (1:400, Jackson ImmunoResearch) diluted in 1 µg/ml Hoechst 33342 (staining of nuclei) at room temperature for 1 h. Staining for polymeric F-actin was performed by incubation with phalloidin tetramethylrhodamine-B-isothiocyanate (50 µg/ml, Sigma-Aldrich) for 40 min at room temperature. Cells for staining against β-tubulin were fixed with cold MeOH and stained with Cy3 conjugated β-tubulin antibody (1:100, Sigma-Aldrich) for 1 h at room temperature. Images were acquired using an inverted fluorescence microscope (Axiovert 200) equipped with a slider module (ApoTome; Carl Zeiss MicroImaging) and a confocal microscope (ECLIPSE Ti; Nikon). Quantifications of volume of nuclei were analyzed with the NIS element software (Nikon).

1.2. Figures

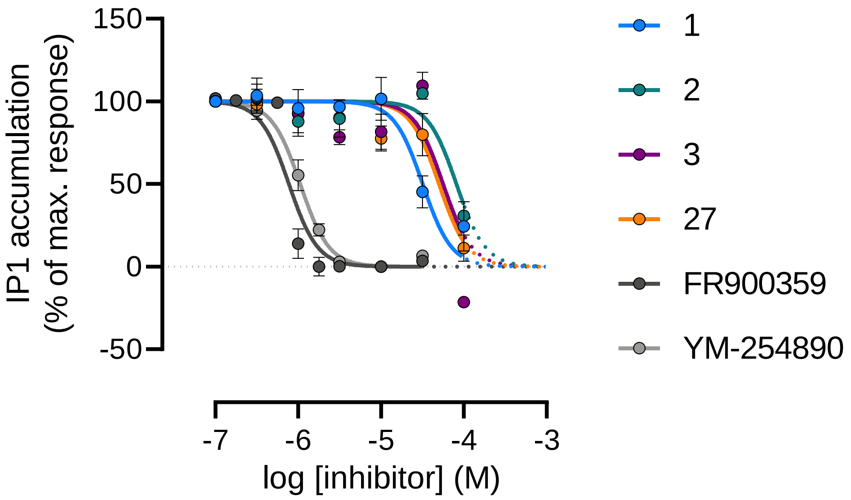

Figure S1. Quantitative G protein inhibition profiles of selected Gα_q inhibitory ligands. The ability of increasing concentrations of BIM-46187 (**1**) as well as its analogs **2**, **3**, and **27** to block agonism induced by CCh at the EC_{100} was assessed in IP1 accumulation assays. Data for the G$\alpha_{q/11}$ family-specific inhibitors FR900359 and YM-254890 are taken from Reher *et al.*[2] and were included for reference purpose. Concentration-inhibition curves were fitted using four parameter logistic regression with top (no inhibition) and bottom (full inhibition) constrained to 100 and 0, respectively, and a constant slope of -2.5 to match the steepness observed for FR900359, YM-254890, and BIM-46187 (**1**). To achieve complete concentration-inhibition curves for **1-3**, and **27**, the fits were extended beyond the highest applicable concentration (100 µM), which is indicated by dashed lines. Data are mean ± s.e.m. of at least 2 independent experiments. The following pIC_{50} values (± s.e.m.) were obtained, 4.496 ± 0.061 (**1**), 4.089 ± 0.064 (**2**), 4.250 ± 0.121 (**3**), 4.300 ± 0.070 (**27**), 6.112 ± 0.027 (FR900359), 5.975 ± 0.025 (YM-254890).

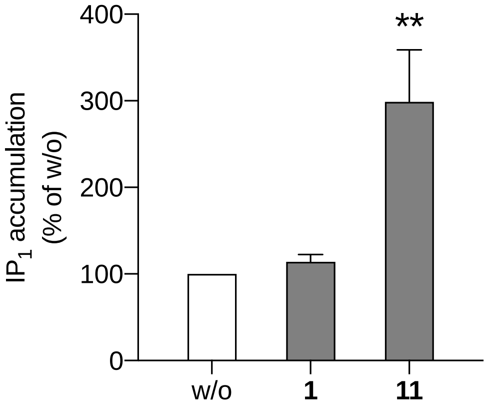

Figure S2. Effect of test compounds on CCh-independent IP1 formation. Compound **11** but not BIM-46187 (**1**) each applied at 100 µM for 2 h elevates cellular IP1 production in HEK293 cells. Data are means ± s.e.m. of four independent experiments. ** P < 0.01 compared to w/o using Dunnett´s multiple comparisons after one-way ANOVA.

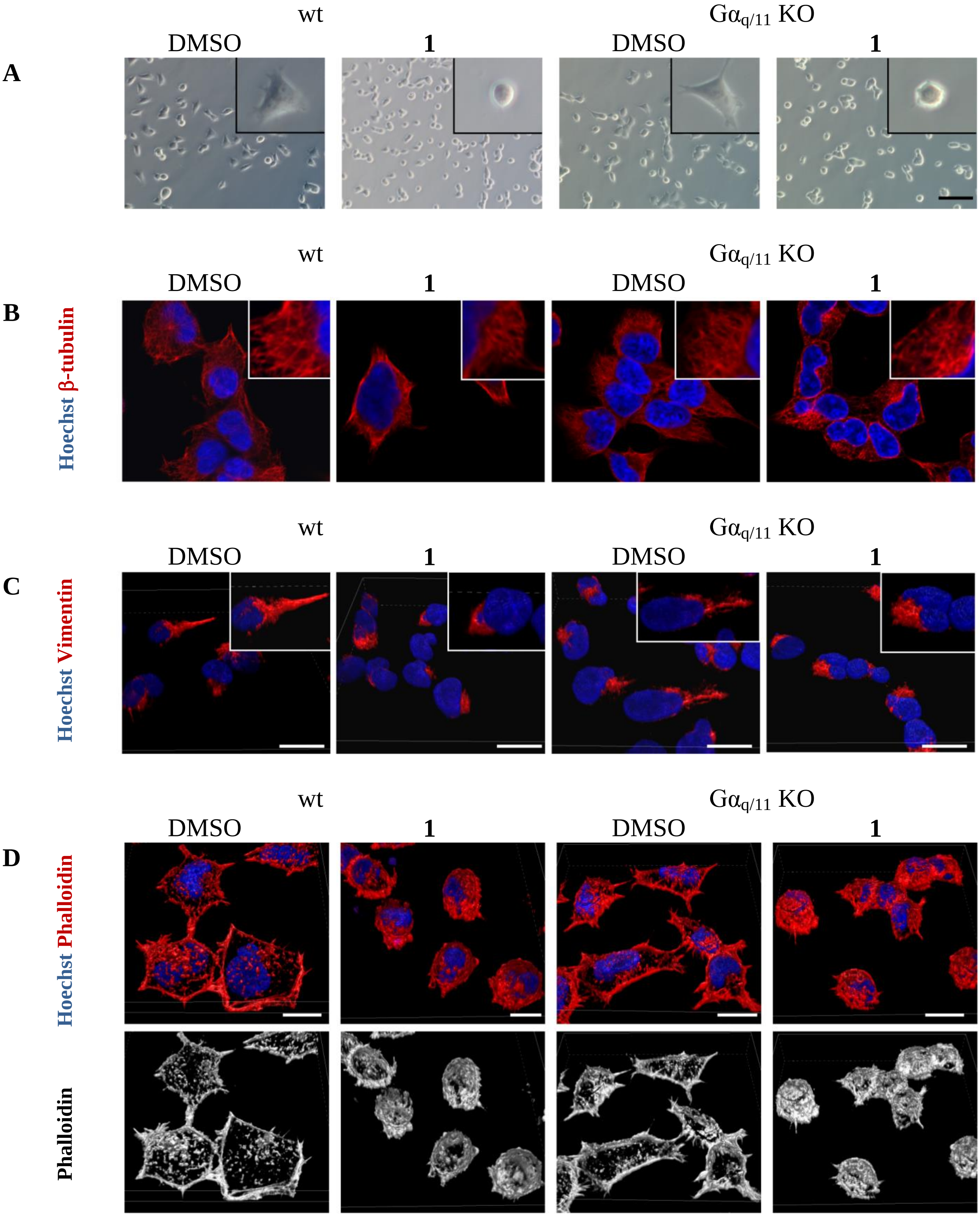
wt
Gαq/11 KO
DMSO
1
DMSO
1
A
B
Hoechst β-tubulin
C
Hoechst Vimentin
D
Hoechst Phalloidin
Phalloidin

Figure S3. Live cell imaging data. (**A**) Prominent morphological changes of wt and $G\alpha_{q/11}$ KO HEK293 cells upon 30 min exposure to the BIM dimer (**1**, 30 µM) compared to controls in presence of DMSO. Scale bar: 100 µm. (**B**) β-Tubulin staining revealed that the microtubular network was unperturbed by compound treatment. Scale bar: 20 µm. (**C**) Vimentin staining showed that **1** induced in wt and $G\alpha_{q/11}$ KO HEK293 cells shortening and perinuclear localization of intermediate filaments. Scale bar: 20 µm. (**D**) Phalloidin staining revealed strong changes of cell shape and loss of stress fibers upon BIM exposure. Scale bar: 20 µm. (**B**-**D**) Hoechst staining was used to demarcate cell nuclei.

2. Chemistry

2. 1. General Methods & Instruments

Thin-layer chromatography was carried out on Merck (Darmstadt, Germany) aluminum sheets, silica gel 60 F254. Detection was performed with UV light at 254 nm. Preparative column chromatography (CC) was performed on Merck silica gel (0.063-0.200 mm, 60 Å). Semi-preparative HPLC purifications were carried out on a system of the SMARTLINE series (Knauer, Berlin, Germany) equipped with two S-1800 pumps with 100 mL pump heads including a two-canal online degasser as well as a static mixing chamber SmartMix 350, an assistant 6000 comprising an S-100 feed pump as well as an electrical injection valve with 2000 µL sample loop, an S-2550 UV detector and an electrical 16-port-1-canal switch valve for sample collection. Data were acquired and processed with ChromGate Software 3.3.2 Agilent Technologies. As the stationary phase, the Eurospher II 100-5 C18 (5 µm, column size 250 × 16 mm, Knauer) connected to a precolumn (30 × 16 mm), was applied. The preparation of the sample solution (mg/mL) and the composition of the mobile phase throughout the purification including the duration time (min) are as indicated for each compound. Each run was performed at room temperature after injection of a definite volume (µL) of the unfiltrated sample solution at a specific flow rate (mL/min), and detection was carried out at a particular wavelength (nm). When a gradient elution was applied, reconditioning of at least 5 min was implemented between the single runs. Melting points were determined on a Büchi (Essen, Germany) 510 oil bath apparatus.

^{1}H NMR and ^{13}C NMR spectra were recorded in DMSO-d_6. ^{1}H NMR (500 MHz) and ^{13}C NMR (125 MHz) spectra were obtained on a Bruker Avance DRX 500 and ^{1}H NMR (600 MHz) and ^{13}C NMR (150 MHz) spectra on a Bruker Avance III 600 NMR spectrometer. Chemical shifts δ are given in ppm referring to the signal center using the solvent peaks for reference: DMSO-d_6 2.49/39.7 ppm. Spectra, in which signal(s) are obscured by the solvent peak, are indicated with diamond(s). ^{13}C NMR signals with asterisk(s) represent resonances of two (three) chemically non-equivalent carbons.

LC-MS analyses were carried out on an API2000 (Applied Biosystems, Darmstadt, Germany) mass spectrometer coupled to an Agilent (Santa Clara, CA, USA) 1100 LC system using a EC50/2 Nucleodur C18 Gravity 3 µm column (Macherey-Nagel, Düren, Germany). Retention times (RT) of all compounds are noted. Purity of the compounds was determined using the diode array detector of the LC-MS instrument between 220 and 400 nm. Samples

were dissolved (1 mg/mL) in MeOH or MeCN, respectively, containing 2 mM ammoniumacetate, and 8 µL of this solution was injected into the column at 25 °C. The mobile phase was a mixture of H_2O containing 2 mM ammoniumacetate (A) and MeOH or MeCN, respectively, containing 2 mM ammoniumacetate (B). Elution was performed with a gradient of either A/B (90:10) to (0:100) [method 1] or A/B (60:40) to (0:100) [method 2] in 10 min, followed by an isocratic flush with A/B (0:100) for a further 10 min, all at a flow rate of 0.3 mL/min. Exclusively positive total ion scans were observed. HRMS were recorded on a microTOF-Q (Bruker, Köln, Germany) mass spectrometer connected to a Dionex (Thermo Scientific, Braunschweig, Germany) Ultimate 3000 LC via an ESI interface using a EC50/2 Nucleodur C18 Gravity 3 µm column (Macherey-Nagel, Düren, Germany). Samples were dissolved (0.6 mg/mL) in MeCN and 1 µL of this solution was injected into the column at 25 °C. The mobile phase was a mixture of H_2O containing 2 mM ammoniumacetate (A) and MeCN (B). Elution was consistently performed by starting isocratic with A/B (90:10) for 1 min, followed by a gradient of A/B (90:10) to (0:100) in 9 min, and terminated with an isocratic flush A/B (0:100) for a further 5 min, all at a flow rate of 0.3 mL/min. Positive as well as negative full scan MS were observed. Protected amino acids were obtained as single enantiomers from Carbolution Chemicals (St. Ingbert, Germany), Bachem (Bubendorf, Switzerland), IRIS Biotech (Marktredwitz, Germany) and Fluorochem (Hadfield, United Kingdom).

2. 2. Preparation of Compounds & Spectral Data

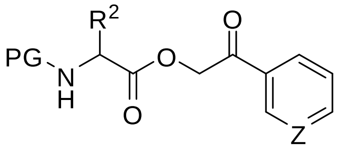

B (**30-37**)

*(S)-2-Oxo-2-phenylethyl 2-(tert-Butyloxycarbonylamino)-3-cyclohexylpropanoate (**B**, R^2 = (S)-$CH_2C_6H_{11}$, PG = Boc, Z = CH; **30**), Typical Procedure to Esters **B***. Boc-L-Cha-OH (8.14 g, 30.0 mmol) was dissolved in anhyd DMF (75 mL) and K_2CO_3 (4.98 g, 36.0 mmol) was added. After 10 min of stirring at room temperature, 2-bromoacetophenone (6.57 g, 33.0 mmol) was added and the reaction mixture was stirred at room temperature for a further 4 h. The mixture was then diluted with H_2O (180 mL) and extracted with EtOAc (360 mL). The organic layer was dried over Na_2SO_4, filtered, and evaporated to dryness. The residue was purified by CC on silica gel using a gradient of petroleum ether/EtOAc (20:1) to (9:1) yielding **30** (11.3 g, 97%) as a white solid; mp 83–85 °C. ^{1}H NMR (500 MHz) δ 0.79 – 0.89 (m, 1H), 0.89 – 0.99 (m, 1H), 1.09 – 1.26 (m, 3H), 1.38 (s, 9H), 1.55 – 1.71 (m, 7H), 1.71 – 1.78 (m, 1H), 4.17 (ddd, *J* = 10.2, 8.2, 4.9 Hz, 1H), 5.42 (d, *J* = 16.9 Hz, 1H), 5.56 (d, *J* = 16.8 Hz, 1H), 7.25 (d, *J* = 8.1 Hz, 1H), 7.52 – 7.57 (m, 2H), 7.66 – 7.70 (m, 1H), 7.95 (dd, *J* = 7.9, 1.4 Hz, 2H). ^{13}C NMR (125 MHz) δ 25.7, 25.9, 26.2, 28.3, 31.6, 33.3, 33.6, 38.3, 51.2, 66.7, 78.3, 127.9, 129.0, 134.0,* 155.7, 172.9, 192.7. LC-MS (ESI) (MeOH; method 1), RT 11.88 min, 91% purity, *m/z* = 390.4 ($[M+H]^+$), 407.4 ($[M+NH_4]^+$).

*(S)-2-Oxo-2-phenylethyl 2-(Benzyloxycarbonylamino)-3-phenylpropanoate (**B**, R^2 = (S)-$CH_2C_6H_5$, PG = Cbz, Z = CH; **31**)*. From Cbz-L-Phe-OH (8.98 g) and 2-bromoacetophenone. Recrystallization from EtOAc/Et_2O. Yield 8.52 g, 68%. Colorless needles; mp 102–104 °C (lit.[3] mp 100 °C). ^{1}H NMR (500 MHz) δ 2.96 (dd, *J* = 14.0, 11.0 Hz, 1H), 3.25 (dd, *J* = 13.9, 3.9 Hz, 1H), 4.44 (ddd, *J* = 10.9, 8.5, 4.0 Hz, 1H), 4.97 (d, *J* = 12.8 Hz, 1H), 4.99 (d, *J* = 13.1 Hz, 1H), 5.53 (d, *J* = 16.7 Hz, 1H), 5.62 (d, *J* = 16.9 Hz, 1H), 7.21 – 7.35 (m, 10H), 7.54 – 7.59 (m, 2H), 7.67 – 7.72 (m, 1H), 7.88 (d, *J* = 8.5 Hz, 1H), 7.96 – 8.00 (m, 2H). ^{13}C NMR (125 MHz) δ 36.6, 55.5, 65.5, 67.1, 126.6, 127.6, 127.9, 128.0, 128.4, 128.4, 129.1, 129.2, 134.0, 134.1, 137.0, 137.7, 156.1, 171.6, 192.6. LC-MS (ESI) (MeOH; method 1), RT 10.97 min, 97% purity, *m/z* = 418.4 ($[M+H]^+$), 435.5 ($[M+NH_4]^+$).

*(S)-2-Oxo-2-phenylethyl 2-(Benzyloxycarbonylamino)-2-cyclohexylethanoate (**B**, R^2 = (S)-C_6H_{11}, PG = Cbz, Z = CH; **32**)*. From Cbz-L-Chg-OH (8.74 g) and 2-bromoacetophenone. CC using a gradient of petroleum ether/EtOAc (10:1) to (7:3). Yield 11.9 g, 97%. Colorless oil. ^{1}H NMR (500 MHz) δ 1.06 – 1.26 (m, 5H), 1.57 – 1.62 (m, 1H), 1.67 – 1.75 (m, 4H),

1.79 – 1.84 (m, 1H), 4.12 (dd, *J* = 8.5, 6.4 Hz, 1H), 5.04 (s, 2H), 5.47 (d, *J* = 16.9 Hz, 1H), 5.55 (d, *J* = 16.9 Hz, 1H), 7.28 – 7.39 (m, 5H), 7.52 – 7.57 (m, 2H), 7.64 – 7.71 (m, 2H), 7.96 (d, *J* = 7.7 Hz, 2H). ^{13}C NMR (125 MHz)♦ δ 25.7,** 27.8, 29.0, 59.1, 65.7, 66.9, 127.9, 127.9,* 128.5, 129.0, 134.0, 137.1, 156.4, 171.3, 192.8. LC-MS (ESI) (MeOH; method 1), RT 11.64 min, 98% purity, *m/z* = 410.4 ($[M+H]^+$), 427.4 ($[M+NH_4]^+$).

*(S)-2-Oxo-2-phenylethyl 2-(Benzyloxycarbonylamino)-4-methylpentanoate (**B**, R^2 = (S)-$CH_2CH(CH_3)_2$, PG = Cbz, Z = CH; **33**).* From Cbz-L-Leu-OH (7.96 g) and 2-bromoacetophenone. CC using a gradient of petroleum ether/EtOAc (20:1) to (10:1). Yield 9.66 g, 84%. Yellowish oil.[3] ^{1}H NMR (600 MHz) δ 0.88 (d, *J* = 6.5 Hz, 3H), 0.91 (d, *J* = 6.6 Hz, 3H), 1.61 – 1.70 (m, 2H), 1.71 – 1.78 (m, 1H), 4.22 (ddd, *J* = 9.5, 8.2, 5.7 Hz, 1H), 5.04 (s, 2H), 5.46 (d, *J* = 16.8 Hz, 1H), 5.58 (d, *J* = 16.9 Hz, 1H), 7.28 – 7.38 (m, 5H), 7.55 (t, *J* = 7.8 Hz, 2H), 7.66 – 7.71 (m, 1H), 7.79 (d, *J* = 8.1 Hz, 1H), 7.95 (dd, *J* = 8.1 Hz, 1.5 Hz, 2H). ^{13}C NMR (150 MHz)♦ δ 21.3, 23.0, 24.4, 52.3, 65.7, 66.9, 127.9, 127.9, 128.0, 128.5, 129.1, 134.0, 134.1, 137.1, 156.3, 172.6, 192.7. LC-MS (ESI) (MeOH; method 1), RT 11.28 min, 98% purity, *m/z* = 384.2 ($[M+H]^+$), 401.2 ($[M+NH_4]^+$).

*(S)-2-Oxo-2-phenylethyl 2-(tert-Butoxycarbonylamino)butanoate (**B**, R^2 = (S)-CH_2CH_3, PG = Boc, Z = CH; **34**).* From Boc-L-Abu-OH (6.10 g) and 2-bromoacetophenone. Yield 6.75 g, 70%. Recrystallization from EtOA/Et_2O. Colorless solid; mp 133–135 °C. ^{1}H NMR (500 MHz) δ 0.96 (t, *J* = 7.4 Hz, 3H), 1.38 (s, 9H), 1.69 (ddd, *J* = 13.7, 8.6, 6.9 Hz, 1H), 1.80 – 1.88 (m, 1H), 4.02 (td, *J* = 8.1, 5.0 Hz, 1H), 5.44 (d, *J* = 16.9 Hz, 1H), 5.57 (d, *J* = 16.8 Hz, 1H), 7.25 (d, *J* = 7.8 Hz, 1H), 7.51 – 7.59 (m, 2H), 7.65 – 7.71 (m, 1H), 7.93 – 8.00 (m, 2H). ^{13}C NMR (125 MHz) δ 10.6, 24.5, 28.3, 55.1, 66.7, 78.3, 127.9, 129.0, 134.0,* 155.7, 172.3, 192.7. LC-MS (ESI) (MeOH; method 1), RT 10.74 min, 97% purity, *m/z* = 322.1 ($[M+H]^+$), 339.1 ($[M+NH_4]^+$).

*2-Oxo-2-phenylethyl 2-(Benzyloxycarbonylamino)-2-methylpropanoate (**B**, R^2 = $(CH_3)_2$, PG = Cbz, Z = CH; **35**).* From Cbz-Aib-OH (7.12 g) and 2-bromoacetophenone. Yield 7.04 g, 66%. Recrystallization from EtOAc/Et_2O. Colorless crystals; mp 102–104 °C. ^{1}H NMR (500 MHz) δ 1.46 (s, 6H), 5.01 (s, 2H), 5.44 (s, 2H), 7.28 – 7.39 (m, 5H), 7.52 – 7.58 (m, 2H), 7.66 – 7.70 (m, 1H), 7.79 (s, 1H), 7.96 (d, *J* = 7.7 Hz, 2H). ^{13}C NMR (125 MHz) δ 25.4, 55.8, 65.3, 66.7, 127.8, 127.9,* 128.5, 129.0, 134.0, 134.1, 137.1, 155.2, 173.8, 193.0. LC-MS (ESI) (MeOH; method 1), RT 10.63 min, 100% purity, *m/z* = 356.1 ($[M+H]^+$), 373.2 ($[M+NH_4]^+$).

*(S)-2-Oxo-2-phenylethyl 2-(Benzyloxycarbonylamino)-3-tert-butoxypropanoate (**B**, R^2 = (S)-CH_2OtBu, PG = Cbz, Z = CH; **36**).* From Cbz-L-Ser(tBu)-OH (8.86 g) and 2-

bromoacetophenone. CC using a gradient of petroleum ether/EtOAc (10:1) to (7:3). Yield 10.5 g, 85%. White solid; mp 88–90 °C. ^{1}H NMR (600 MHz) δ 1.13 (s, 9H), 3.64 (dd, *J* = 9.4, 6.8 Hz, 1H), 3.69 (dd, *J* = 9.5, 4.2 Hz, 1H), 4.36 (ddd, *J* = 8.4, 6.7, 4.2 Hz, 1H), 5.05 (s, 2H), 5.50 (d, *J* = 16.7 Hz, 1H), 5.55 (d, *J* = 16.8 Hz, 1H), 7.28 – 7.38 (m, 5H), 7.55 (t, *J* = 7.8 Hz, 2H), 7.61 (d, *J* = 8.4 Hz, 1H), 7.66 – 7.71 (m, 1H), 7.94 – 7.99 (m, 2H). ^{13}C NMR (150 MHz) δ 27.3, 55.2, 61.4, 65.7, 67.1, 73.2, 127.9, 128.0,* 128.5, 129.0, 134.0, 134.1, 137.1, 156.2, 170.2, 192.5. LC-MS (ESI) (MeOH; method 1), RT 11.28 min, 99% purity, *m/z* = 414.0 ($[M+H]^+$), 431.1 ($[M+NH_4]^+$).

*(S)-4-tert-Butyl 1-(2-Oxo-2-phenylethyl) 2-(benzyloxycarbonylamino)succinate (**B**, R^2 = (S)-CH_2CO_2tBu, PG = Cbz, Z= CH; **37**).* From Cbz-L-Asp-OH (9.70 g) and 2-bromoacetophenone. CC using a gradient of petroleum ether/EtOAc (20:1) to (10:1). Yield 13.0 g, 98%. Yellowish oil. ^{1}H NMR (500 MHz) δ 1.38 (s, 9H), 2.60 – 2.68 (m, 1H), 2.83 – 2.90 (m, 1H), 4.58 – 4.64 (m, 1H), 5.03 (d, *J* = 10.5 Hz, 1H), 5.07 (d, *J* = 10.3 Hz, 1H), 5.49 (d, *J* = 14.0 Hz, 1H), 5.58 (d, *J* = 16.9 Hz, 1H), 7.28 – 7.38 (m, 5H), 7.55 (t, *J* = 5.9 Hz, 2H), 7.68 (t, *J* = 6.3 Hz, 1H), 7.87 (d, *J* = 7.0 Hz, 1H), 7.94 – 7.97 (m, 2H). ^{13}C NMR (125 MHz) δ 27.7, 35.9, 50.6, 65.7, 67.2, 80.7, 127.8, 127.9,* 128.5, 129.0, 134.0, 134.1, 137.0, 155.9, 162.4, 168.9, 170.4. LC-MS (ESI) (MeOH; method 1), RT 11.47 min, 84% purity, *m/z* = 442.3 ($[M+H]^+$), 459.4 ($[M+NH_4]^+$).

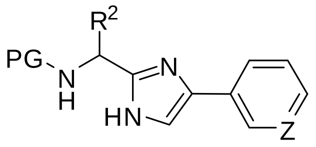

C (**38-45**)

*(S)-tert-Butyl 2-cyclohexyl-1-(4-phenyl-1H-imidazol-2-yl)ethylcarbamate (**C**, R^2 = (S)-$CH_2C_6H_{11}$, PG = Boc, Z = CH; **38**). Typical Procedure to Imidazoles **C**.* Compound **30** (9.74 g, 25.0 mmol) was dissolved in anhyd toluene (120 mL) and ammonium acetate (28.9 g, 375 mmol) was added to the solution. The reaction mixture was heated to reflux for 3 h with a drying tube attached to the condenser. The solvent was evaporated and the residue was suspended in EtOAc (400 mL). The organic layer was washed with H_2O (2 × 200 mL) and brine (200 mL), and then dried over Na_2SO_4, filtered, and concentrated. The crude residue was purified by preparative CC using petroleum ether/EtOAc (10:1) to obtain **38** as yellow solid (6.37 g, 69%); mp 198–200 °C. ^{1}H NMR (600 MHz) δ 0.81 – 0.97 (m, 2H), 1.04 – 1.28 (m, 3H), 1.38 (s, 9H), 1.53 – 1.71 (m, 7H), 1.72 – 1.79 (m, 1H), 4.68 – 4.77 (m, 1H), 6.99 (d, *J* = 8.8 Hz, 1H), 7.15 (t, *J* = 7.4 Hz, 1H), 7.31 (t, *J* = 7.6 Hz, 2H), 7.40 (s, 1H), 7.70 (d, *J* = 7.6

Hz, 2H), 11.78 (s, 1H). ^{13}C NMR (150 MHz) δ 26.2, 26.4, 26.6, 28.7, 32.6, 33.5, 34.2, 42.4, 46.9, 78.4, 112.9, 124.6, 126.4, 128.9, 135.1, 139.6, 150.3, 155.6. LC-MS (ESI) (MeOH; method 1), RT 12.00 min, 100% purity, *m/z* = 370.2 ([M+H]$^+$).

*(S)-Benzyl 2-Phenyl-1-(4-phenyl-1H-imidazol-2-yl)ethylcarbamate (**C**, R^2 = (S)-$CH_2C_6H_5$, PG = Cbz, Z = CH; **39**).* From **31** (10.4 g). CC using a gradient of petroleum ether/EtOAc (9:1) to (7:3). Yield 8.84 g, 89%. Yellow semi-solid. ^{1}H NMR (500 MHz) δ 3.05 (dd, *J* = 13.6, 9.2 Hz, 1H), 4.87 – 4.93 (m, 1H), 4.95 (d, *J* = 13.0 Hz, 1H), 5.00 (d, *J* = 12.8 Hz), 7.14 – 7.35 (m, 13H), 7.48 (s, 1H), 7.72 – 7.78 (m, 3H), 11.91 (s, 1H). One proton signal is obscured by the water peak. ^{13}C NMR (125 MHz)♦ δ 51.1, 65.3, 112.6, 124.4, 126.0, 126.3, 127.5, 127.7, 128.2, 128.4, 128.5, 129.4, 135.1, 137.3, 138.4, 139.7, 148.6, 155.8. LC-MS (ESI) (MeOH; method 2), RT 9.49 min, 99% purity, *m/z* = 398.0 ([M+H]$^+$).

*(S)-Benzyl Cyclohexyl(4-phenyl-1H-imidazol-2-yl)methylcarbamate (**C**, R^2 = (S)-C_6H_{11}, PG = Cbz, Z = CH; **40**).* From **32** (10.2 g). CC using a gradient of petroleum ether/EtOAc (9:1) to (7:3). Yield 7.11 g, 73%. Yellowish solid; mp 184–187 °C. ^{1}H NMR (500 MHz) δ 0.86 – 1.05 (m, 2H), 1.06 – 1.20 (m, 3H), 1.30 – 1.37 (m, 1H), 1.53 – 1.85 (m, 5H), 4.47 (t, *J* = 8.6 Hz, 1H), 5.00 (d, *J* = 12.7 Hz, 1H), 5.04 (d, *J* = 12.7 Hz, 1H), 7.15 (t, *J* = 7.4 Hz, 1H), 7.19 – 7.41 (m, 7H), 7.43 – 7.49 (m, 2H), 7.73 (d, *J* = 7.6 Hz, 2H), 11.80 (s, 1H). ^{13}C NMR (125 MHz) δ 25.5, 25.6, 26.0, 29.0, 29.5, 41.6, 54.6, 65.5, 112.1, 124.4, 125.9, 127.8, 127.9, 128.4,* 135.1, 137.2, 139.7, 148.5, 156.1. LC-MS (ESI) (MeOH; method 1), RT 11.72 min, 98% purity, *m/z* = 390.3 ([M+H]$^+$).

*(S)-Benzyl 3-Methyl-1-(4-phenyl-1H-imidazol-2-yl)butylcarbamate (**C**, R^2 = (S)-$CH_2CH(CH_3)_2$, PG = Cbz, Z = CH; **41**).* From **33** (9.59 g). CC using a gradient of petroleum ether/EtOAc (10:1) to (7:3). Yield 7.45 g, 82%. Yellow oil. ^{1}H NMR (500 MHz) δ 0.88 (d, *J* = 6.7 Hz, 3H), 0.90 (d, *J* = 6.7 Hz, 3H), 1.53 – 1.63 (m, 1H), 1.64 – 1.76 (m, 2H), 4.73 (app q, *J* = 7.8 Hz, 1H), 5.01 (d, *J* = 13.2 Hz, 1H), 5.05 (d, *J* = 12.5 Hz, 1H), 7.15 (t, *J* = 7.4 Hz, 1H), 7.26 – 7.40 (m, 7H), 7.43 – 7.47 (m, 1H), 7.58 (d, *J* = 8.6 Hz, 1H), 7.73 (d, *J* = 7.7 Hz, 2H), 11.84 (s, 1H). ^{13}C NMR (125 MHz) δ 21.9, 22.9, 24.4, 43.1, 47.8, 65.5, 112.4, 124.3, 125.9, 127.8, 127.8, 128.4,* 135.1, 137.3, 139.6, 149.4, 155.9. LC-MS (ESI) (MeOH; method 1), RT 11.38 min, 98% purity, *m/z* = 364.1 ([M+H]$^+$).

*(S)-tert-Butyl 1-(4-Phenyl-1H-imidazol-2-yl)propylcarbamate (**C**, R^2 = (S)-CH_2CH_3, PG = Boc, Z = CH; **42**).* From **34** (8.03 g). CC using a gradient of petroleum ether/EtOAc (9:1) to (7:3). Yield 7.06 g, 94%. Yellow oil. ^{1}H NMR (500 MHz) δ 0.83 (t, *J* = 7.3 Hz, 3H), 1.38 (s, 9H), 1.67 – 1.78 (m, 1H), 1.80 – 1.89 (m, 1H), 4.48 – 4.54 (m, 1H), 6.95 – 7.02 (m, 1H), 7.14 (t, *J* = 8.0 Hz, 1H), 7.31 (t, *J* = 7.6 Hz, 2H), 7.45 (s, 1H), 7.68 – 7.74 (m, 2H), 11.74 (s, 1H).

^{13}C NMR (125 MHz) δ 10.6, 27.6, 28.4, 50.5, 78.1, 112.4, 124.3, 125.9, 128.5, 135.2, 139.4, 149.3, 155.3. LC-MS (ESI) (MeOH; method 1), RT 10.71 min, 99% purity, *m/z* = 301.9 ($[M+H]^+$).

*Benzyl 2-(4-Phenyl-1H-imidazol-2-yl)propan-2-ylcarbamate (**C**, R^2 = $(CH_3)_2$, PG = Cbz, Z = CH; **43**).* From **35** (8.88 g). CC using a gradient of petroleum ether/EtOAc (4:1) to (1:1). Yield 6.54 g, 78%. Yellowish solid; mp 153–155 °C. ^{1}H NMR (500 MHz) δ 1.60 (s, 6H), 4.97 (s, 2H), 7.14 (tt, *J* = 7.4, 1.3 Hz, 1H), 7.18 – 7.42 (m, 7H), 7.44 (d, *J* = 2.0 Hz, 1H), 7.49 (s, 1H), 7.71 – 7.76 (m, 2H), 11.77 (s, 1H). ^{13}C NMR (125 MHz) δ 27.7, 51.9, 65.0, 112.6, 124.3, 125.8, 127.7,* 128.4,* 135.3, 137.3, 138.8, 152.8, 154.6. LC-MS (ESI) (MeOH; method 1), RT 10.29 min, 100% purity, *m/z* = 336.0 ($[M+H]^+$).

*(R)-Benzyl 2-tert-Butoxy-1-(4-phenyl-1H-imidazol-2-yl)ethylcarbamate (**C**, R^2 = (R)-CH_2OtBu, PG = Cbz, Z = CH; **44**).* From **36** (10.3 g). CC using a gradient of petroleum ether/EtOAc (9:1) to (4:1). Yield 9.25 g, 94%. Yellow solid; mp 120–123 °C. ^{1}H NMR (600 MHz) δ 1.09 (s, 9H), 3.54 – 3.62 (m, 1H), 3.71 (dd, *J* = 9.4, 5.6 Hz, 1H), 4.75 (app q, *J* = 7.1 Hz, 1H), 5.00 – 5.10 (m, 2H), 7.15 (t, *J* = 7.4 Hz, 1H), 7.27 – 7.38 (m, 7H), 7.46 – 7.51 (m, 2H), 7.74 (d, *J* = 7.6 Hz, 2H), 11.86 (s, 1H). ^{13}C NMR (150 MHz) δ 27.8, 50.9, 63.7, 65.9, 73.3, 113.0, 124.7, 126.3, 128.1, 128.2, 128.8, 128.8, 135.4, 137.6, 140.0, 147.3, 156.3. LC-MS (ESI) (MeOH; method 1), RT 11.19 min, 99% purity, *m/z* = 394.1 ($[M+H]^+$).

*(S)-tert-Butyl 3-(Benzyloxycarbonylamino)-3-(4-phenyl-1H-imidazol-2-yl)propanoate (**C**, R^2 = (S)-CH_2CO_2tBu, PG = Cbz, Z = CH; **45**).* From **37** (11.0 g). CC using a gradient of petroleum ether/EtOAc (10:1) to (1:1). Yield 9.59 g, 91%. Yellow solid; mp 98–100 °C. ^{1}H NMR (500 MHz) δ 1.34 (s, 9H), 2.70 (dd, *J* = 15.5, 8.1 Hz, 1H), 2.97 (dd, *J* = 15.5, 6.7 Hz, 1H), 4.99 – 5.10 (m, 3H), 7.15 (t, *J* = 7.0 Hz, 1H), 7.28 – 7.35 (m, 7H), 7.48 (d, *J* = 2.1 Hz, 1H), 7.71 – 7.76 (m, 3H), 11.93 (s, 1H). ^{13}C NMR (125 MHz) δ 27.8, 40.9, 46.5, 65.6, 80.0, 113.0, 124.3, 126.0, 127.8, 127.9, 128.4,* 135.0, 137.1, 139.5, 147.7, 155.7, 169.4. LC-MS (ESI) (MeOH; method 1), RT 9.74 min, 98% purity, *m/z* = 422.1 ($[M+H]^+$).

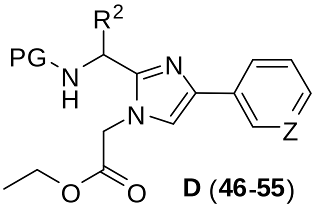

D (**46**-**55**)

*(S)-Ethyl 2-(2-(1-(tert-Butoxycarbonylamino)-2-cyclohexylethyl)-4-phenyl-1H-imidazol-1-yl)acetate (**D**, R^2 = (S)-$CH_2C_6H_{11}$, PG = Boc, Z = CH; **46**). Typical Procedure to N-Alkylated Imidazoles **D**.* Compound **38** (5.54 g, 15.0 mmol) was dissolved in anhyd DMF (25 mL) and

Cs_2CO_3 (12.2 g, 37.5 mmol) was added to the solution. After stirring at room temperature for 30 min, ethyl bromoacetate (3.01 g, 18.0 mmol) dissolved in anhyd DMF (17.5 mL) was added dropwise over a period of 30 min. The mixture was stirred for a further 2.5 h at room temperature, then quenched with iced H_2O (120 mL) and extracted with EtOAc (3 × 120 mL). The organic layer was washed with brine (120 mL) and H_2O (2 × 120 mL), and dried over Na_2SO_4, filtered and evaporated to dryness. The crude residue was purified by preparative CC using a gradient of petroleum ether/EtOAc (10:1) to (7:1) to yield **46** as yellowish oil (6.08 g, 89%). ^{1}H NMR (500 MHz) δ 0.77 – 0.88 (m, 1H), 0.89 – 1.01 (m, 1H), 1.05 – 1.19 (m, 3H), 1.22 (t, *J* = 7.1 Hz, 3H), 1.25 – 1.34 (m, 1H), 1.35 (s, 9H), 1.54 – 1.74 (m, 5H), 1.74 – 1.80 (m, 1H), 1.86 (ddd, *J* = 14.3, 9.6, 5.0 Hz, 1H), 4.09 – 4.21 (m, 2H), 4.62 – 4.70 (m, 1H), 4.99 (s, 2H), 7.15 – 7.20 (m, 1H), 7.23 (d, *J* = 8.8 Hz, 1H), 7.31 – 7.35 (m, 2H), 7.49 (s, 1H), 7.68 – 7.71 (m, 2H). ^{13}C NMR (125 MHz) δ 14.1, 25.9, 26.1, 26.2, 28.3, 32.1, 33.4, 33.7, 40.5, 43.6, 46.8, 61.3, 78.1, 117.8, 124.2, 126.3, 128.6, 134.5, 138.4, 149.5, 155.6, 168.4. LC-MS (ESI) (MeOH; method 1), RT 12.13 min, 91% purity, *m/z* = 456.1 ([M+H]$^+$).

*(R)-Ethyl 2-(2-(1-(Benzyloxycarbonylamino)-2-cyclohexylethyl)-4-phenyl-1H-imidazol-1-yl)acetate (**D**, R^2 = (R)-$CH_2C_6H_{11}$, PG = Cbz, Z = CH; **47**).* From the corresponding imidazole (6.05 g).[4] CC using petroleum ether/EtOAc (10:1). Yield 5.66 g, 77%. Yellowish oil. ^{1}H NMR (500 MHz) δ 0.77 – 0.88 (m, 1H), 0.90 – 0.98 (m, 1H), 1.02 – 1.18 (m, 3H), 1.21 (t, *J* = 7.1 Hz, 3H), 1.25 – 1.36 (m, 1H), 1.54 – 1.67 (m, 4H), 1.68 – 1.75 (m, 2H), 1.91 (ddd, *J* = 14.3, 9.6, 5.0 Hz, 1H), 4.10 – 4.16 (m, 2H), 4.70 (td, *J* = 9.2, 5.3 Hz, 1H), 4.91 – 5.11 (m, 4H), 7.15 – 7.20 (m, 1H), 7.26 – 7.36 (m, 7H), 7.51 (s, 1H), 7.68 – 7.72 (m, 2H), 7.76 (d, *J* = 8.6 Hz, 1H). ^{13}C NMR (125 MHz) δ 14.1, 25.8, 26.0, 26.2, 32.0, 33.4, 33.6, 40.5, 44.1, 46.9, 61.3, 65.5, 117.9, 124.2, 126.3, 127.6, 127.8, 128.4, 128.6, 134.5, 137.3, 138.5, 149.3, 156.1, 168.4. LC-MS (ESI) (MeOH; method 1), RT 12.05 min, 90% purity, *m/z* = 490.3 ([M+H]$^+$).

*(S)-Ethyl 2-(2-(1-(Benzyloxycarbonylamino)-2-phenylethyl)-4-phenyl-1H-imidazol-1-yl)acetate (**D**, R^2 = (S)-$CH_2C_6H_5$, PG = Cbz, Z = CH; **48**).* From **39** (5.96 g). CC using a gradient of petroleum ether/EtOAc (9:1) to (4:1). Yield 5.73 g, 79%. Orange semi-solid. ^{1}H NMR (500 MHz) δ 1.19 (t, *J* = 7.1 Hz, 3H), 3.21 – 3.28 (m, 2H), 4.08 – 4.14 (m, 2H), 4.82 – 5.03 (m, 5H), 7.14 – 7.32 (m, 11H), 7.33 – 7.39 (m, 2H), 7.55 (s, 1H), 7.73 – 7.77 (m, 2H), 7.90 (d, *J* = 8.9 Hz, 1H). ^{13}C NMR (125 MHz) δ 14.1, 38.8, 46.9, 48.2, 61.3, 65.3, 118.1, 124.3, 126.3, 126.4, 127.3, 127.7, 128.1, 128.4, 128.6, 129.5, 134.5, 137.1, 138.5, 138.6, 148.7, 155.9, 168.4. LC-MS (ESI) (MeOH; method 2), RT 10.08 min, 98% purity, *m/z* = 484.4 ([M+H]$^+$).

*(S)-Ethyl 2-(2-((Benzyloxycarbonylamino)(cyclohexyl)methyl)-4-phenyl-1H-imidazol-1-yl)acetate (**D**, R^2 = (S)-C_6H_{11}, PG = Cbz, Z = CH; **49**).* From **40** (5.84 g). CC using petroleum ether/EtOAc (4:1). Yield 4.28 g, 60%. Yellowish solid; mp 124–127 °C. ^{1}H NMR (500 MHz) δ 0.80 – 1.01 (m, 2H), 1.05 – 1.17 (m, 3H), 1.20 (t, *J* = 7.1 Hz, 3H), 1.42 – 1.49 (m, 1H), 1.53 – 1.63 (m, 2H), 1.66 – 1.74 (m, 1H) and 1.90 – 1.97 (m, 1H), 2.01 – 2.09 (m, 1H), 4.13 (q, *J* = 7.1 Hz, 2H), 4.37 (app t, *J* = 9.3 Hz, 1H), 4.93 – 5.09 (m, 4H), 7.16 – 7.20 (m, 1H), 7.25 – 7.36 (m, 7H), 7.50 (s, 1H), 7.67 – 7.73 (m, 3H, 2-H). ^{13}C NMR (125 MHz)♦ δ 14.1, 25.6, 25.6, 26.1, 29.1, 29.8, 46.8, 51.7, 61.3, 65.5, 117.7, 124.3, 126.3, 127.7, 127.9, 128.4, 128.6, 134.5, 137.2, 138.8, 148.5, 156.3, 168.3. LC-MS (ESI) (MeOH; method 1), RT 12.15 min, 95% purity, *m/z* = 476.3 ($[M+H]^+$).

*(S)-Ethyl 2-(2-(1-(Benzyloxycarbonylamino)-3-methylbutyl)-4-phenyl-1H-imidazol-1-yl)acetate (**D**, R^2 = (S)-$CH_2CH(CH_3)_2$, PG = Cbz, Z = CH; **50**).* From **41** (5.45 g). CC using a gradient of petroleum ether/EtOAc (10:1) to (4:1). Yield 5.87 g, 87%. Yellow oil. ^{1}H NMR (500 MHz) δ 0.86 (d, *J* = 6.4 Hz), 0.89 (d, *J* = 6.5 Hz, 3H), 1.20 (t, *J* = 7.1 Hz, 3H), 1.59 – 1.66 (m, 1H), 1.67 – 1.74 (m, 1H), 1.93 (ddd, *J* = 14.0, 9.6, 5.0 Hz, 1H), 4.10 – 4.16 (m, 2H), 4.68 (td, *J* = 9.2, 5.3 Hz, 1H), 4.96 (d, *J* = 12.6 Hz, 1H), 5.00 – 5.08 (m, 3H), 7.15 – 7.20 (m, 1H), 7.26 – 7.36 (m, 7H), 7.51 (s, 1H), 7.68 – 7.72 (m, 2H), 7.77 (d, *J* = 8.6 Hz, 1H). ^{13}C NMR (125 MHz) δ 14.1, 21.8, 23.0, 24.3, 42.0, 44.9, 46.9, 61.3, 65.6, 117.9, 124.3, 126.3, 127.7, 127.9, 128.4, 128.6, 134.5, 137.1, 138.5, 149.2, 156.1, 168.4. LC-MS (ESI) (MeOH; method 1), RT 11.65 min, 90% purity, *m/z* = 450.0 ($[M+H]^+$).

*(S)-Ethyl 2-(2-(1-(tert-Butoxycarbonylamino)propyl)-4-phenyl-1H-imidazol-1-yl)acetate (**D**, R^2 = (S)-CH_2CH_3, PG = Boc, Z = CH; **51**).* From **42** (4.52 g). CC using a gradient of petroleum ether/EtOAc (10:1) to (4:1). Yield 4.13 g, 71%. Yellow crystals; mp 88–91 °C. ^{1}H NMR (500 MHz) δ 0.86 (t, *J* = 7.3 Hz, 3H), 1.22 (t, *J* = 7.1 Hz, 3H), 1.35 (s, 9H), 1.80 – 1.90 (m, 1H), 1.91 – 1.99 (m, 1H), 4.11 – 4.19 (m, 2H), 4.47 (app q, *J* = 7.8 Hz, 1H), 5.00 (s, 2H), 7.15 – 7.19 (m, 1H), 7.20 (d, *J* = 8.8 Hz, 1H), 7.31 – 7.36 (m, 2H), 7.50 (s, 1H), 7.68 – 7.72 (m, 2H). ^{13}C NMR (125 MHz) δ 10.8, 14.1, 26.3, 28.3, 46.8, 47.7, 61.2, 78.1, 117.8, 124.2, 126.3, 128.6, 134.5, 138.5, 149.1, 155.6, 168.4. LC-MS (ESI) (MeOH; method 1), RT 11.06 min, 98% purity, *m/z* = 388.2 ($[M+H]^+$).

*Ethyl 2-(2-(2-(Benzyloxycarbonylamino)propan-2-yl)-4-phenyl-1H-imidazol-1-yl)acetate (**D**, R^2 = $(CH_3)_2$, PG = Cbz, Z = CH; **52**).* From **43** (5.03 g). CC using a gradient of petroleum ether/EtOAc (9:1) to (3:2). Yield 6.07 g, 96%. Yellowish oil. ^{1}H NMR (600 MHz) δ 1.18 (t, *J* = 7.1 Hz, 3H), 1.58 (s, 6H), 4.11 (q, *J* = 7.1 Hz, 2H), 4.91 (s, 2H), 4.99 (s, 2H), 7.17 (tt, *J* = 7.1, 1.4 Hz, 1H), 7.22 – 7.42 (m, 7H), 7.46 (s, 1H), 7.70 (d, *J* = 7.6 Hz, 2H), 7.77 (s, 1H). ^{13}C

NMR (150 MHz) δ 14.1, 28.0, 47.9, 52.2, 61.3, 65.3, 119.4, 124.2, 126.2, 127.9,* 128.4, 128.6, 134.6, 137.1, 137.2, 150.6, 154.3, 168.5. LC-MS (ESI) (MeOH; method 1), RT 10.92 min, 98% purity, *m/z* = 422.1 ($[M+H]^+$).

*(R)-Ethyl 2-(2-(1-(Benzyloxycarbonylamino)-2-tert-butoxyethyl)-4-phenyl-1H-imidazol-1-yl)acetate (**D**, R^2 = (R)-CH_2OtBu, PG = Cbz, Z = CH; **53**).* From **44** (5.90 g). CC using a gradient of petroleum ether/EtOAc (10:1) to (4:1). Yield 6.11 g, 85%. Yellow oil. ^{1}H NMR (500 MHz) δ 1.11 (s, 9H), 1.20 (t, *J* = 7.1 Hz, 3H), 3.68 – 3.78 (m, 2H), 4.13 (app q, *J* = 7.1 Hz, 2H), 4.71 (td, *J* = 8.1, 5.7 Hz, 1H), 4.96 (d, *J* = 12.7 Hz, 1H), 4.99 (s, 2H), 5.07 (d, *J* = 12.5 Hz, 1H), 7.16 – 7.21 (m, 1H), 7.26 – 7.37 (m, 7H), 7.54 (s, 1H), 7.66 (d, *J* = 8.7 Hz, 1H), 7.69 – 7.72 (m, 2H). ^{13}C NMR (125 MHz) δ 14.1, 27.5, 47.0, 47.9, 61.3, 62.9, 65.6, 73.0, 118.0, 124.3, 126.4, 127.7, 127.9, 128.4, 128.6, 134.4, 137.2, 138.7, 147.1, 156.2, 168.3. LC-MS (ESI) (MeOH; method 1), RT 11.42 min, 95% purity, *m/z* = 480.0 ($[M+H]^+$).

*(S)-tert-Butyl 3-(Benzyloxycarbonylamino)-3-(1-(2-ethoxy-2-oxoethyl)-4-phenyl-1H-imidazol-2-yl)propanoate (**D**, R^2 = (S)-CH_2CO_2tBu, PG = Cbz, Z = CH; **54**).* From **45** (6.32 g). CC using petroleum ether/EtOAc (10:1). Yield 7.08 g, 93%. Yellow semi-solid. ^{1}H NMR (500 MHz) δ 1.19 – 1.22 (m, 3H), 1.32 (s, 9H), 2.84 (dd, *J* = 15.8, 7.6 Hz, 1H), 3.00 (dd, *J* = 15.8, 7.2 Hz, 1H), 4.10 – 4.14 (m, 2H), 4.93 – 5.10 (m, 5H), 7.18 (t, *J* = 6.1 Hz, 1H), 7.26 – 7.37 (m, 7H), 7.55 (s, 1H), 7.68 – 7.72 (m, 2H), 7.87 (d, *J* = 7.6 Hz, 1H). ^{13}C NMR (125 MHz) δ 14.4, 28.1, 40.6, 44.2, 47.2, 61.6, 66.0, 80.3, 118.8, 124.6, 126.7, 128.1, 128.3, 128.8, 128.9, 134.7, 137.3, 138.7, 147.9, 156.1, 168.5, 169.8. LC-MS (ESI) (MeOH; method 1), RT 11.87 min, 86% purity, *m/z* = 508.2 ($[M+H]^+$).

*(S)-Ethyl 2-(2-(1-(Benzyloxycarbonylamino)-2-cyclohexylethyl)-4-(pyridin-3-yl)-1H-imidazol-1-yl)acetate (**D**, R^2 = (S)-$CH_2C_6H_{11}$, PG = Cbz, Z = N; **55**).* From the corresponding imidazole (6.07 g).[4] CC using a gradient of petroleum ether/EtOAc (1:1) to EtOAc (100%). Yield 5.67 g, 77%. Orange oil. ^{1}H NMR (500 MHz) δ 0.77 – 0.88 (m, 1H), 0.90 – 1.00 (m, 1H), 1.04 – 1.18 (m, 3H), 1.21 (t, *J* = 7.1 Hz, 3H), 1.26 – 1.36 (m, 1H), 1.53 – 1.78 (m, 6H), 1.92 (ddd, *J* = 14.2, 9.8, 5.3 Hz, 1H), 4.08 – 4.19 (m, 2H), 4.72 (td, *J* = 9.2, 5.3 Hz, 1H), 4.94 (d, *J* = 12.7 Hz, 1H), 4.98 – 5.11 (m, 3H), 7.26 – 7.34 (m, 5H), 7.36 (ddd, *J* = 7.9, 4.8, 0.9 Hz, 1H), 7.66 (s, 1H), 7.80 (d, *J* = 8.6 Hz, 1H), 8.03 (ddd, *J* = 7.9, 2.3, 1.7 Hz, 1H), 8.39 (dd, *J* = 4.7, 1.6 Hz, 1H), 8.91 (dd, *J* = 2.3, 0.9 Hz, 1H). ^{13}C NMR (125 MHz) δ 14.1, 25.8, 26.0, 26.2, 32.0, 33.4, 33.6, 41.4, 44.1, 47.0, 61.4, 65.5, 118.9, 123.8, 127.6, 127.9, 128.4, 130.1, 131.3, 135.6, 137.2, 145.7, 147.4, 149.9, 156.2, 168.3. LC-MS (ESI) (MeOH; method 1), RT 11.65 min, 97% purity, *m/z* = 491.3 ($[M+H]^+$).

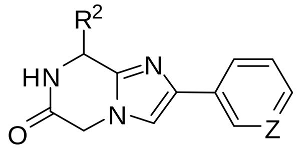

E (**56**-**65**)

*(S)-8-(Cyclohexylmethyl)-2-phenyl-7,8-dihydroimidazo[1,2-a]pyrazin-6(5H)-one (**E**, R^2 = (S)-$CH_2C_6H_{11}$, Z = CH; **56**). Typical Procedure to Lactams **E** from Boc-protected precursors.* Compound **46** (4.56 g, 10.0 mmol) was dissolved in anhyd CH_2Cl_2 (75 mL) and TFA (15 mL) was slowly added. The reaction mixture was stirred at room temperature for 2 h. After concentrating the mixture *in vacuo,* the residue was diluted with CH_2Cl_2 (4 × 50 mL) and again evaporated. The residue was redissolved in CH_2Cl_2 (60 mL) and washed with H_2O (2 × 60 mL). The organic layer was dried over Na_2SO_4, filtered and evaporated to dryness. Next, the residue was redissolved in anhyd CH_2Cl_2 (32.5 mL), treated with triethylamine (4.09 mL, 3.04 g, 30.0 mmol) and stirred at room temperature for 2 h. The solvent was evaporated and the crude residue was purified by CC on silica gel using a gradient of EtOAc (100%) to EtOAc/MeOH (9:1) to obtain **56** as a yellow solid (2.41 g, 78%); mp 195–198 °C (lit.[5] mp 206–207 °C). ^{1}H NMR (500 MHz) δ 0.83 – 0.95 (m, 2H), 1.08 – 1.27 (m, 3H), 1.56 – 1.72 (m, 7H), 1.71 – 1.79 (m, 1H), 4.59 – 4.65 (m, 2H), 4.70 (dd, *J* = 17.7, 1.3 Hz, 1H), 7.16 – 7.21 (m, 1H), 7.31 – 7.36 (m, 2H), 7.51 (s, 1H), 7.69 – 7.74 (m, 2H), 8.59 (d, *J* = 2.9 Hz, 1H). ^{13}C NMR (125 MHz) δ 25.7, 25.9, 26.2, 32.6, 32.7, 33.1, 44.3, 47.0, 48.0, 114.2, 124.4, 126.5, 128.6, 134.4, 140.7, 143.1, 165.0. LC-MS (ESI) (MeOH; method 1), RT 11.11 min, 98% purity, *m/z* = 310.1 ($[M+H]^+$).

*(R)-8-(Cyclohexylmethyl)-2-phenyl-7,8-dihydroimidazo[1,2-a]pyrazin-6(5H)-one (**E**, R^2 = (R)-$CH_2C_6H_{11}$, Z = CH; **57**). Typical Procedure to Lactams **E** from Cbz-protected precursors.* Compound **47** (4.90 g, 10.0 mmol) was dissolved in anhyd MeOH (80 mL) containing Pd/C (10% Pd) and was hydrogenated under atmospheric pressure at room temperature for 24 h. The catalyst was filtered off through celite and rinsed with MeOH (2 × 70 mL). The removal of the solvent *in vacuo* yielded a yellow solid without further purification (2.85 g, 92%); mp 194–197 °C. ^{1}H NMR (500 MHz) δ 0.83 – 0.95 (m, 2H), 1.08 – 1.27 (m, 3H), 1.56 – 1.64 (m, 5H), 1.64 – 1.72 (m, 2H), 1.73 – 1.79 (m, 1H), 4.60 – 4.65 (m, 2H), 4.66 – 4.72 (m, 1H), 7.17 – 7.21 (m, 1H), 7.31 – 7.37 (m, 2H), 7.51 (s, 1H), 7.69 – 7.74 (m, 2H), 8.59 (d, *J* = 2.9 Hz, 1H). ^{13}C NMR (125 MHz) δ 25.7, 25.9, 26.2, 32.6, 32.7, 33.1, 44.2, 47.0, 48.0, 114.2, 124.4, 126.5, 128.6, 134.4, 140.7, 143.1, 165.0. LC-MS (ESI) (MeOH; method 1), RT 10.94 min, 98% purity, *m/z* = 310.0 ($[M+H]^+$).

*(S)-8-Benzyl-2-phenyl-7,8-dihydroimidazo[1,2-a]pyrazin-6(5H)-one (**E**, R^2 = (S)-$CH_2C_6H_5$, Z = CH; **58**).* From **48** (4.84 g). Yield 2.82 g, 93%. Yellow solid; mp 228–230 °C. ^{1}H NMR (500 MHz) δ 3.09 (dd, *J* = 13.4, 4.4 Hz, 1H), 3.23 (dd, *J* = 13.4, 3.8 Hz, 1H), 3.26 – 3.30 (m, 1H), 4.33 (dd, *J* = 17.4 Hz, 1.3 Hz, 1H), 5.01 (app q, *J* = 3.7 Hz, 1H), 6.80 – 6.86 (m, 2H), 7.12 – 7.25 (m, 4H), 7.33 – 7.39 (m, 3H), 7.73 – 7.79 (m, 2H), 8.55 (d, *J* = 3.0 Hz, 1H). ^{13}C NMR (125 MHz) δ 42.1, 46.3, 51.2, 113.8, 124.5, 126.6, 126.8, 128.1, 128.7, 130.1, 134.4, 135.7, 141.0, 141.6, 164.7. LC-MS (ESI) (MeOH; method 1), RT 10.25 min, 99% purity, *m/z* = 303.9 ($[M+H]^+$).

*(S)-8-Cyclohexyl-2-phenyl-7,8-dihydroimidazo[1,2-a]pyrazin-6(5H)-one (**E**, R^2 = (S)-C_6H_{11}, Z = CH; **59**).* From **49** (4.76 g). CC using a gradient of EtOAc (100%) to EtOAc/MeOH (9:1). Yield 798 mg, 27%. Yellowish solid; mp > 250 °C. ^{1}H NMR (500 MHz) δ 0.90 – 1.06 (m, 2H), 1.09 – 1.21 (m, 2H), 1.21 – 1.31 (m, 1H), 1.38 – 1.46 (m, 1H), 1.55 – 1.62 (m, 1H), 1.62 – 1.73 (m, 3H), 1.76 – 1.85 (m, 1H), 4.40 (t, *J* = 3.4 Hz, 1H), 4.63 (dd, *J* = 17.7, 1.2 Hz, 1H), 4.65 – 4.70 (m, 1H), 7.16 – 7.22 (m, 1H), 7.31 – 7.37 (m, 2H), 7.52 (s, 1H), 7.70 – 7.75 (m, 2H), 8.53 (d, *J* = 3.3 Hz, 1H). ^{13}C NMR (125 MHz) δ 25.7, 25.7, 25.9, 27.3, 27.9, 44.9, 47.0, 55.3, 114.2, 124.4, 126.5, 128.6, 134.4, 140.8, 141.6, 165.1. LC-MS (ESI) (MeOH; method 1), RT 10.66 min, 95% purity, *m/z* = 296.0 ($[M+H]^+$).

*(S)-8-Isobutyl-2-phenyl-7,8-dihydroimidazo[1,2-a]pyrazin-6(5H)-one (**E**, R^2 = (S)-$CH_2CH(CH_3)_2$, Z = CH; **60**).* From **50** (4.50 g). CC using a gradient of petroleum ether/EtOAc (2:3) to (1:4). Yield 485 mg, 18%. Yellow solid; mp > 250 °C. ^{1}H NMR (500 MHz) δ 0.87 (d, *J* = 6.6 Hz), 0.91 (d, *J* = 6.6 Hz, 3H), 1.62 – 1.75 (m, 2H), 1.88 – 1.98 (m, 1H), 4.56 – 4.60 (m, 1H), 4.62 (dd, *J* = 17.5, 1.4 Hz, 1H), 4.71 (dd, *J* = 17.6, 1.1 Hz, 1H), 7.16 – 7.23 (m, 1H), 7.30 – 7.37 (m, 2H), 7.52 (s, 1H), 7.68 – 7.76 (m, 2H), 8.58 (d, *J* = 2.9 Hz, 1H). ^{13}C NMR (125 MHz) δ 22.5, 22.8, 23.5, 45.7, 47.0, 48.6, 114.2, 124.4, 126.5, 128.6, 134.4, 140.7, 143.0, 165.0. LC-MS (ESI) (MeOH; method 1), RT 9.99 min, 96% purity, *m/z* = 269.9 ($[M+H]^+$).

*(S)-8-Ethyl-2-phenyl-7,8-dihydroimidazo[1,2-a]pyrazin-6(5H)-one (**E**, R^2 = (S)-CH_2CH_3, Z = CH; **61**).* From **51** (3.87 g). CC using EtOAc (100%). Yield 1.86 g, 77%. Yellowish solid; mp 180–182 °C. ^{1}H NMR (600 MHz) δ 0.83 (t, *J* = 7.3 Hz, 3H), 1.82 – 1.92 (m, 2H), 4.60 (app q, *J* = 4.3 Hz, 1H), 4.64 (dd, *J* = 17.6, 1.5 Hz, 1H), 4.70 (d, *J* = 17.5 Hz, 1H), 7.19 (t, *J* = 7.4 Hz, 1H), 7.34 (t, *J* = 7.6 Hz, 2H), 7.52 (s, 1H), 7.73 (d, *J* = 7.6 Hz, 2H), 8.53 (d, *J* = 2.7 Hz, 1H). ^{13}C NMR (150 MHz) δ 8.5, 28.9, 47.0, 51.2, 114.2, 124.4, 126.6, 128.7, 134.4, 140.9, 142.3, 165.0. LC-MS (ESI) (MeOH; method 1), RT 8.99 min, 98% purity, *m/z* = 242.0 ($[M+H]^+$).

*8,8-Dimethyl-2-phenyl-7,8-dihydroimidazo[1,2-a]pyrazin-6(5H)-one (**E**, R^2 = $(CH_3)_2$, Z = CH; **62**).* From **52** (4.21 g). Yield 2.29 g, 95%. Yellowish solid; mp 226–229 °C. ^{1}H NMR (600 MHz) δ 1.56 (s, 6H), 4.69 (s, 2H), 7.16 – 7.22 (m, 1H), 7.33 (t, *J* = 7.7 Hz), 7.49 (s, 1H), 7.71 – 7.75 (m, 2H), 8.62 (s, 1H). ^{13}C NMR (150 MHz) δ 30.1, 47.1, 52.4, 114.0, 124.4, 126.5, 128.6, 134.4, 140.5, 147.0, 164.4. LC-MS (ESI) (MeOH; method 1), RT 8.68 min, 100% purity, *m/z* = 242.2 ($[M+H]^+$).

*(R)-8-(tert-Butoxymethyl)-2-phenyl-7,8-dihydroimidazo[1,2-a]pyrazin-6(5H)-one (**E**, R^2 = (S)-CH_2OtBu, Z = CH; **63**).* From **53** (4.80 g). Yield 2.54 g, 85%. Yellowish solid; mp 228–230 °C. ^{1}H NMR (600 MHz) δ 1.00 (s, 9H), 3.60 – 3.65 (m, 2H), 4.51 – 4.56 (m, 1H), 4.61 (dd, *J* = 17.3, 1.0 Hz, 1H), 4.64 (app q, *J* = 3.1 Hz, 1H), 7.16 – 7.21 (m, 1H), 7.31 – 7.36 (m, 2H), 7.52 (s, 1H), 7.70 – 7.75 (m, 2H), 8.36 (d, *J* = 3.2 Hz, 1H). ^{13}C NMR (150 MHz) δ 27.3, 47.5, 51.4, 65.6, 73.3, 114.3, 124.4, 126.5, 128.7, 134.5, 140.7, 141.5, 166.1. LC-MS (ESI) (MeOH; method 1), RT 10.07 min, 96% purity, *m/z* = 300.0 ($[M+H]^+$).

*(S)-tert-Butyl 2-(6-Oxo-2-phenyl-5,6,7,8-tetrahydroimidazo[1,2-a]pyrazin-8-yl)acetate (**E**, R^2 = (S)-CH_2CO_2tBu, Z = CH; **64**).* From **54** (5.08 g). Yield 3.24 g, 99%. Yellow solid; mp 178–180 °C. ^{1}H NMR (500 MHz) δ 1.33 (s, 9H), 2.78 (dd, *J* = 13.5, 4.8 Hz, 1H), 2.88 (dd, *J* = 13.5, 1.4 Hz, 1H), 4.60 (dd, *J* = 14.5, 1.4 Hz, 1H), 4.67 (dd, *J* = 14.5, 1.5 Hz, 1H), 4.89 – 4.93 (m, 1H), 7.15 – 7.21 (m, 1H), 7.30 – 7.35 (m, 2H), 7.52 (s, 1H), 7.67 – 7.73 (m, 2H), 8.48 (d, *J* = 1.9 Hz, 1H). ^{13}C NMR (125 MHz) δ 27.8, 40.8, 47.1, 47.4, 80.4, 114.1, 124.4, 126.6, 128.6, 134.4, 140.9, 141.7, 164.8, 169.0. LC-MS (ESI) (MeOH; method 1), RT 10.01 min, 95% purity, *m/z* = 328.1 ($[M+H]^+$).

*(S)-8-(Cyclohexylmethyl)-2-(pyridin-3-yl)-7,8-dihydroimidazo[1,2-a]pyrazin-6(5H)-one (**E**, R^2 = (S)-$CH_2C_6H_{11}$, Z = N; **65**).* From **55** (4.91 g). Yield 2.87 g, 93%. Yellowish solid; mp 189–192 °C. ^{1}H NMR (600 MHz) δ 0.84 – 0.94 (m, 2H), 1.08 – 1.26 (m, 3H), 1.56 – 1.72 (m, 7H), 1.73 – 1.78 (m, 1H), 4.61 – 4.67 (m, 2H), 4.73 (dd, *J* = 17.6, 1.2 Hz, 1H), 7.36 (dd, *J* = 7.9, 4.7 Hz, 1H), 7.67 (s, 1H), 8.05 (dt, *J* = 7.9, 2.0 Hz, 1H), 8.39 (dd, *J* = 4.7, 1.6 Hz, 1H), 8.63 (d, *J* = 2.9 Hz, 1H, NH), 8.93 (d, *J* = 2.1 Hz, 1H). ^{13}C NMR (150 MHz) δ 25.8, 25.9, 26.2, 32.5, 32.8, 33.1, 44.3, 47.1, 48.0, 115.2, 123.9, 130.1, 131.5, 137.8, 143.7, 145.9, 147.6, 164.9. LC-MS (ESI) (MeOH; method 1), RT 10.25 min, 97% purity, *m/z* = 310.7 ($[M+H]^+$).

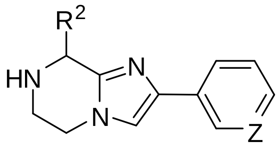

F (**66**-**75**)

*(S)-8-(Cyclohexylmethyl)-2-phenyl-5,6,7,8-tetrahydroimidazo[1,2-a]pyrazine (**F**, R^2 = (S)-$CH_2C_6H_{11}$, Z = CH; **66** ≡ **J**). Typical Procedure to 5,6,7,8-Tetrahydroimidazo[1,2-a]pyrazines **F**.* Compound **56** (1.54 g, 5.00 mmol) was dissolved in anhyd THF (80 mL) under argon atm. 1 M Borane-THF complex solution (30 mL) was added dropwise over a period of 10 min, followed by heating the mixture to reflux for 48 h. After evaporating the solvent *in vacuo*, the residue was redissolved in anhyd MeOH (60 mL) and added to a Schlenk flask containing Pd/C (10% Pd) under argon atm. The reaction mixture was stirred at room temperature over night. The catalyst was filtered off through celite and rinsed with MeOH (2 × 50 mL). The solvent was evaporated to dryness and the crude residue was purified by CC on silica gel using EtOAc/MeOH (9:1). Compound **66** was isolated as yellowish oil (1.26 g, 85%).[5] ^{1}H NMR (600 MHz) δ 0.86 – 0.94 (m, 1H), 0.94 – 1.02 (m, 1H), 1.12 – 1.20 (m, 1H), 1.21 – 1.30 (m, 2H), 1.45 (ddd, *J* = 13.7, 9.8, 4.0 Hz, 1H), 1.61 – 1.69 (m, 5H), 1.83 – 1.88 (m, 1H), 1.88 – 1.94 (m, 1H), 2.94 (ddd, *J* = 13.5, 8.9, 4.8 Hz, 1H), 3.17 (dt, *J* = 13.3, 4.1 Hz, 1H), 3.81 – 3.90 (m, 3H), 7.12 – 7.17 (m, 1H), 7.30 (t, *J* = 7.7 Hz, 2H), 7.42 (s, 1H), 7.69 (dd, *J* = 8.1, 1.4 Hz, 2H). The NH signal is not recognizable. ^{13}C NMR (150 MHz) δ 25.9, 26.2, 26.4, 32.1, 33.3, 34.1, 41.3, 41.5, 45.2, 51.1, 114.9, 124.3, 126.0, 128.5, 135.1, 138.7, 147.5. LC-MS (ESI) (MeOH; method 1), RT 11.43 min, 96% purity, *m/z* = 296.1 ($[M+H]^+$).

*(R)-8-(Cyclohexylmethyl)-2-phenyl-5,6,7,8-tetrahydroimidazo[1,2-a]pyrazine (**F**, R^2 = (R)-$CH_2C_6H_{11}$, Z = CH; **67**).* From **57** (1.54 g). CC using EtOAc/MeOH (9:1). Yield 1.21 g, 82%. Yellowish oil. ^{1}H NMR (500 MHz) δ 0.87 – 1.04 (m, 2H), 1.13 – 1.30 (m, 3H), 1.44 – 1.50 (m, 1H), 1.60 – 1.73 (m, 5H), 1.83 – 1.88 (m, 1H), 1.91 (ddd, *J* = 13.5, 9.1, 4.0 Hz, 1H), 2.94 (ddd, *J* = 13.5, 8.8, 5.0 Hz, 1H), 3.17 (dt, *J* = 13.3, 4.1 Hz, 1H), 3.80 – 3.90 (m, 3H), 7.12 – 7.17 (m, 1H), 7.28 – 7.33 (m, 2H), 7.42 (s, 1H), 7.67 – 7.71 (m, 2H). The NH signal is not recognizable. ^{13}C NMR (125 MHz) δ 25.9, 26.2, 26.4, 32.1, 33.3, 34.1, 41.3, 41.4, 45.2, 51.1, 114.8, 124.2, 126.0, 128.5, 135.0, 138.7, 147.5. LC-MS (ESI) (MeOH; method 1), RT 11.17 min, 96% purity, *m/z* = 296.1 ($[M+H]^+$).

*(S)-8-Benzyl-2-phenyl-5,6,7,8-tetrahydroimidazo[1,2-a]pyrazine (**F**, R^2 = (S)-$CH_2C_6H_5$, Z = CH; **68**).* From **58** (1.52 g). CC using EtOAc/MeOH (9:1). Yield 1.35 g, 93%. Yellow oil. ^{1}H NMR (500 MHz) δ 2.85 (dd, *J* = 13.8, 9.7 Hz, 1H), 2.91 (ddd, *J* = 13.5, 9.1, 4.8 Hz, 1H),

3.16 (dt, J = 13.3, 4.0 Hz, 1H), 3.45 (dd, J = 13.9, 3.5 Hz, 1H), 3.83 – 3.93 (m, 2H), 4.11 (dd, J = 9.7, 3.4 Hz, 1H), 7.15 – 7.22 (m, 2H), 7.26 – 7.35 (m, 6H), 7.47 (s, 1H), 7.73 – 7.76 (m, 2H). The NH signal is not recognizable. ^{13}C NMR (125 MHz)♦ δ 41.3, 45.2, 55.1, 115.0, 124.3, 126.1, 126.2, 128.2, 128.5, 129.6, 134.9, 138.9, 139.1, 146.4. LC-MS (ESI) (MeOH; method 1), RT 10.24 min, 96% purity, *m/z* = 289.9 ([M+H]$^+$).

*(S)-8-Cyclohexyl-2-phenyl-5,6,7,8-tetrahydroimidazo[1,2-a]pyrazine (**F**, R^2 = (S)-C_6H_{11}, Z = CH; **69**).* From **59** (1.48 g). CC using EtOAc/MeOH (9:1). Yield 1.27 g, 90%. Yellow oil. ^{1}H NMR (500 MHz) δ 1.03 – 1.20 (m, 2H), 1.21 – 1.37 (m, 3H), 1.58 – 1.82 (m, 5H), 1.96 – 2.06 (m, 1H), 2.87 – 2.95 (m, 1H), 3.17 (ddd, J = 12.9, 4.5, 3.2 Hz, 1H), 3.73 (d, J = 3.9 Hz, 1H), 3.81 – 3.92 (m, 2H), 7.11 – 7.17 (m, 1H), 7.27 – 7.33 (m, 2H), 7.42 (s, 1H), 7.66 – 7.71 (m, 2H). The NH signal is not recognizable. ^{13}C NMR (125 MHz) δ 26.3, 26.4, 26.4, 27.4, 29.5, 41.4, 41.9, 45.2, 58.7, 114.8, 124.3, 126.0, 128.5, 135.1, 138.9, 146.1. LC-MS (ESI) (MeOH; method 1), RT 10.85 min, 99% purity, *m/z* = 281.9 ([M+H]$^+$).

*(S)-8-Isobutyl-2-phenyl-5,6,7,8-tetrahydroimidazo[1,2-a]pyrazine (**F**, R^2 = (S)-$CH_2CH(CH_3)_2$, Z = CH; **70**).* From **60** (1.35 g). CC using EtOAc/MeOH (9:1). Yield 856 mg, 67%. Yellow oil. ^{1}H NMR (500 MHz) δ 0.94 (d, J = 6.7 Hz, 3H), 0.95 (d, J = 6.6 Hz, 3H), 1.48 (ddd, J = 18.6, 9.5, 4.7 Hz, 1H), 1.83 – 1.91 (m, 1H), 1.95 – 2.04 (m, 1H), 2.95 (ddd, J = 13.5, 8.8, 4.9 Hz, 1H), 3.17 (dt, J = 13.3, 4.1 Hz, 1H), 3.83 – 3.93 (m, 3H), 7.12 – 7.17 (m, 1H), 7.30 (t, J = 7.7 Hz, 2H), 7.43 (s, 1H), 7.67 – 7.71 (m, 2H). The NH signal is not recognizable. ^{13}C NMR (125 MHz) δ 21.8, 23.7, 23.9, 41.3, 43.0, 45.2, 51.7, 114.8, 124.3, 126.0, 128.5, 135.0, 138.7, 147.4. LC-MS (ESI) (MeOH; method 1), RT 10.18 min, 95% purity, *m/z* = 255.9 ([M+H]$^+$).

*(S)-8-Ethyl-2-phenyl-5,6,7,8-tetrahydroimidazo[1,2-a]pyrazine (**F**, R^2 = (S)-CH_2CH_3, Z = CH; **71**).* From **61** (1.21 g). CC using EtOAc/MeOH (9:1). Yield 875 mg, 77%. Yellow oil. ^{1}H NMR (500 MHz) δ 1.00 (t, J = 7.4 Hz, 3H), 1.59 – 1.71 (m, 1H), 1.97 – 2.08 (m, 1H), 2.95 (ddd, J = 13.5, 8.9, 4.9 Hz, 1H), 3.19 (dt, J = 13.1, 4.1 Hz, 1H), 3.75 (dd, J = 8.0, 4.1 Hz, 1H), 3.83 – 3.95 (m, 2H), 7.11 – 7.18 (m, 1H), 7.31 (t, J = 7.7 Hz, 2H), 7.43 (s, 1H), 7.66 – 7.74 (m, 2H). The NH signal is not recognizable. ^{13}C NMR (125 MHz) δ 10.5, 26.8, 41.5, 45.2, 55.1, 114.8, 124.3, 126.0, 128.5, 135.0, 138.9, 147.0. LC-MS (ESI) (MeOH; method 1), RT 8.91 min, 97% purity, *m/z* = 228.1 ([M+H]$^+$).

*8,8-Dimethyl-2-phenyl-5,6,7,8-tetrahydroimidazo[1,2-a]pyrazine (**F**, R^2 = $(CH_3)_2$, Z = CH; **72**).* From **62** (1.21 g). CC using EtOAc/MeOH (9:1). Yield 750 mg, 66%. Orange oil. ^{1}H NMR (600 MHz) δ 1.39 (s, 6H), 3.08 (t, J = 5.4 Hz, 2H), 3.88 (t, J = 5.4 Hz, 2H), 7.14 (tt, J = 7.3, 1.3 Hz, 1H), 7.28 – 7.32 (m, 2H), 7.38 (s, 1H), 7.69 (dd, J = 8.2, 1.4 Hz, 2H). The NH

signal is not recognizable. ^{13}C NMR (150 MHz) δ 28.9, 38.1, 45.6, 51.7, 114.5, 124.3, 126.0, 128.5, 135.0, 138.6, 150.7. LC-MS (ESI) (MeOH; method 1), RT 8.88 min, 100% purity, *m/z* = 228.0 ([M+H]$^+$).

*(R)-8-(tert-Butoxymethyl)-2-phenyl-5,6,7,8-tetrahydroimidazo[1,2-a]pyrazine (**F**, R^2 = (R)-CH_2OtBu, Z = CH; **73**).* From **63** (1.50 g). CC using EtOAc/MeOH (9:1). Yield 1.36 g, 95%. Yellow oil. ^{1}H NMR (600 MHz) δ 1.16 (s, 9H), 2.96 (ddd, *J* = 13.1, 8.8, 4.6 Hz, 1H), 3.24 (dt, *J* = 13.0, 4.1 Hz, 1H), 3.49 (app t, *J* = 8.7 Hz, 1H), 3.86 – 3.94 (m, 3H), 3.96 (dd, *J* = 8.6, 3.0 Hz, 1H), 7.15 (tt, *J* = 7.1, 1.3 Hz, 1H), 7.31 (t, *J* = 7.7 Hz, 2H), 7.48 (s, 1H), 7.68 – 7.72 (m, 2H). The NH signal is not recognizable. ^{13}C NMR (150 MHz) δ 27.6, 41.1, 45.2, 54.4, 63.5, 72.8, 115.2, 124.3, 126.2, 128.5, 134.9, 139.1, 144.0. LC-MS (ESI) (MeOH; method 1), RT 10.35 min, 92% purity, *m/z* = 286.0 ([M+H]$^+$).

*(S)-tert-Butyl 2-(2-Phenyl-5,6,7,8-tetrahydroimidazo[1,2-a]pyrazin-8-yl)acetate (**F**, R^2 = (S)-CH_2CO_2tBu, Z = CH; **74**).* From **64** (1.64 g). CC using EtOAc/MeOH (10:0.5). Yield 830 mg, 53%. Yellow-brownish semi-solid. ^{1}H NMR (500 MHz) δ 1.42 (s, 9H), 2.53 (dd, *J* = 15.8, 8.7 Hz, 1H), 2.88 (dd, *J* = 15.8, 4.4 Hz, 1H), 2.96 – 3.03 (m, 1H), 3.14 – 3.23 (m, 1H), 3.84 – 3.94 (m, 2H), 4.35 (t, *J* = 5.3 Hz, 1H), 7.10 – 7.18 (m, 1H), 7.28 – 7.33 (m, 2H), 7.47 (s, 1H), 7.65 – 7.71 (m, 2H). The NH signal is not recognizable. ^{13}C NMR (125 MHz) δ 28.0, 40.2, 41.4, 45.2, 51.1, 80.1, 115.2, 124.3, 128.2, 128.6, 134.9, 139.0, 145.7, 170.4. LC-MS (ESI) (MeOH; method 1), RT 10.56 min, 86% purity, *m/z* = 314.0 ([M+H]$^+$).

*(S)-8-(Cyclohexylmethyl)-2-(pyridin-3-yl)-5,6,7,8-tetrahydroimidazo[1,2-a]pyrazine (**F**, R^2 = (S)-$CH_2C_6H_{11}$, Z = N; **75**).* From **65** (1.55 g). CC using EtOAc/MeOH (9:1). Yield 963 mg, 65%. Yellow oil. ^{1}H NMR (600 MHz) δ 0.83 – 1.02 (m, 2H), 1.11 – 1.29 (m, 3H), 1.46 (ddd, *J* = 14.1, 10.0, 4.3 Hz, 1H), 1.60 – 1.72 (m, 5H), 1.82 – 1.87 (m, 1H), 1.90 (ddd, *J* = 13.5, 9.0, 3.9 Hz, 1H), 2.94 (ddd, *J* = 13.5, 9.0, 4.7 Hz, 1H), 3.17 (dt, *J* = 13.3, 4.0 Hz, 1H), 3.85 – 3.95 (m, 3H), 7.33 (dd, *J* = 7.9, 4.7 Hz, 1H), 7.58 (s, 1H), 8.02 (dt, *J* = 7.9, 2.0 Hz, 1H), 8.35 (dd, *J* = 4.8, 1.6 Hz, 1H), 8.90 (d, *J* = 2.1 Hz, 1H). The NH signal is not recognizable. ^{13}C NMR (150 MHz) δ 25.9, 26.2, 26.4, 32.1, 33.3, 34.1, 41.2, 41.4, 45.3, 51.1, 115.9, 123.7, 130.6, 131.2, 135.8, 145.8, 147.0, 148.1. LC-MS (ESI) (MeOH; method 1), RT 10.38 min, 95% purity, *m/z* = 297.0 ([M+H]$^+$).

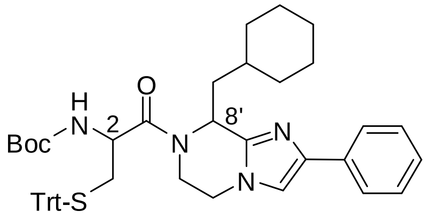

H (**76**-**79**)

*tert-Butyl (R)-1-((S)-8-(Cyclohexylmethyl)-2-phenyl-5,6-dihydroimidazo[1,2-a]pyrazin-7(8H)-yl)-1-oxo-3-(tritylthio)propan-2-ylcarbamate (**H**, 2R,8'S; **76**). Typical Procedure to Protected Cysteine Derivatives **H**.* Boc-L-Cys(Trt)-OH (2.09 g, 4.50 mmol) and DCC (928 mg, 4.50 mmol) were stirred in anhyd CH_2Cl_2 (28 mL) under argon atm for 30 min. Compound **66** (443 mg, 1.50 mmol) dissolved in anhyd CH_2Cl_2 (18 mL) and DIPEA (0.26 mL, 194 mg, 1.50 mmol) were added and the reaction mixture was stirred at room temperature and argon atm for 48 h. After removing the solvent *in vacuo*, the residue was treated with a mixture of petroleum ether/EtOAc (7:3) (200 mL). The white urea was filtered off, the filtrate was evaporated to dryness and the crude oily residue was purified by preparative CC on silica gel using a gradient of petroleum ether/EtOAc (4:1) to (7:3) to obtain **76** as yellowish oil (756 mg, 68%).[19] ^{1}H NMR (500 MHz) δ 0.79 – 0.93 (m, 2H), 1.08 – 1.22 (m, 3H), 1.37 (s, 9H), 1.51 – 1.69 (m, 7H), 1.88 – 1.95 (m, 1H), 2.42 – 2.49 (m, 2H), 3.51 – 3.62 (m, 2H), 3.95 – 3.99 (m, 2H), 4.14 – 4.21 (m, 1H), 5.56 (dd, *J* = 9.6, 5.4 Hz, 1H), 7.15 – 7.19 (m, 1H), 7.22 – 7.36 (m, 18H), 7.50 (s, 1H), 7.68 – 7.71 (m, 2H). ^{13}C NMR (125 MHz) δ 25.6, 25.8, 26.2, 28.2, 32.5, 33.1, 33.1, 33.3, 38.0, 40.7, 44.6, 47.8, 50.0, 66.4, 78.5, 114.8, 124.3, 126.3, 126.9, 128.2, 128.6, 129.2, 134.5, 139.8, 144.4, 144.6, 155.0, 169.7. LC-MS (ESI) (MeOH; method 2), RT 12.10 min, 95% purity, *m/z* = 741.6 ([M+H]$^+$).

*tert-Butyl (S)-1-((S)-8-(Cyclohexylmethyl)-2-phenyl-5,6-dihydroimidazo[1,2-a]pyrazin-7(8H)-yl)-1-oxo-3-(tritylthio)propan-2-ylcarbamate (**H**, 2S,8'S; **77**).* From **66** and Boc-D-Cys(Trt)-OH. CC using a gradient of petroleum ether/EtOAc (4:1) to (7:3). Yield 878 mg, 79%. Yellowish oil. ^{1}H NMR (500 MHz) δ 0.77 – 0.96 (m, 2H), 1.01 – 1.09 (m, 2H), 1.14 – 1.29 (m, 1H), 1.32 (s, 9H), 1.39 – 1.63 (m, 6H), 1.70 (ddd, *J* = 14.6, 11.0, 3.9 Hz, 1H), 1.92 – 1.99 (m, 1H), 2.38 (dd, *J* = 12.7, 7.5 Hz, 1H), 2.50 – 2.54 (m, 1H), 3.41 – 3.48 (m, 1H), 3.68 – 3.75 (m, 1H), 3.75 – 3.81 (m, 1H), 3.98 – 4.05 (m, 1H), 4.13 (app q, *J* = 7.6 Hz, 1H), 5.62 (dd, *J* = 11.0, 4.5 Hz, 1H), 7.14 – 7.18 (m, 1H), 7.24 – 7.37 (m, 18H), 7.50 (s, 1H), 7.65 – 7.69 (m, 2H). ^{13}C NMR (125 MHz) δ 25.7, 25.9, 26.2, 28.2, 32.1, 33.4,* 33.5, 38.3, 40.8, 44.5, 47.4, 50.3, 66.6, 78.7, 114.8, 124.3, 126.4, 127.0, 128.2, 128.5, 129.3, 134.5, 139.8, 144.5, 144.6, 154.9, 169.6. LC-MS (ESI) (MeOH; method 2), RT 12.55 min, 96% purity, *m/z* = 741.6 ([M+H]$^+$).

*tert-Butyl (R)-1-((R)-8-(Cyclohexylmethyl)-2-phenyl-5,6-dihydroimidazo[1,2-a]pyrazin-7(8H)-yl)-1-oxo-3-(tritylthio)propan-2-ylcarbamate (**H**, 2R,8'R; **78**).* From **67** and Boc-L-Cys(Trt)-OH. CC using petroleum ether/EtOAc (4:1). Yield 722 mg, 65%. Yellowish oil. ^{1}H NMR (500 MHz) δ 0.78 – 0.87 (m, 1H), 0.87 – 0.99 (m, 1H), 1.00 – 1.12 (m, 2H), 1.14 – 1.27 (m, 1H), 1.32 (s, 9H), 1.46 – 1.57 (m, 5H), 1.56 – 1.63 (m, 1H), 1.69 (ddd, *J* = 14.6, 11.1, 4.0 Hz, 1H), 1.92 – 2.01 (m, 1H), 2.38 (dd, *J* = 12.7, 7.5 Hz, 1H), 2.50 – 2.55 (m, 1H), 3.41 – 3.50 (m, 1H), 3.68 – 3.80 (m, 2H), 4.00 – 4.05 (m, 1H), 4.13 (app q, *J* = 7.6 Hz, 1H), 5.62 (dd, *J* = 11.0, 4.4 Hz, 1H), 7.14 – 7.18 (m, 1H), 7.24 – 7.38 (m, 18H), 7.50 (s, 1H), 7.66 – 7.69 (m, 2H). ^{13}C NMR (125 MHz) δ 25.7, 25.9, 26.2, 28.2, 32.1, 33.4,* 33.5, 38.2, 40.8, 44.5, 47.4, 50.1, 66.6, 78.7, 114.8, 124.3, 126.4, 127.0, 128.2, 128.5, 129.3, 134.5, 139.8, 144.5, 144.6, 154.9, 169.6. LC-MS (ESI) (MeOH; method 1), RT 13.06 min, 96% purity, *m/z* = 741.3 ([M+H]$^+$).

*tert-Butyl (S)-1-((R)-8-(Cyclohexylmethyl)-2-phenyl-5,6-dihydroimidazo[1,2-a]pyrazin-7(8H)-yl)-1-oxo-3-(tritylthio)propan-2-ylcarbamate (**H**, 2S,8'R; **79**).* From **67** and Boc-D-Cys(Trt)-OH. CC using petroleum ether/EtOAc (4:1). Yield 700 mg, 63%. Yellowish oil. ^{1}H NMR (500 MHz) δ 0.77 – 0.94 (m, 2H), 1.04 – 1.21 (m, 3H), 1.37 (s, 9H), 1.46 – 1.76 (m, 7H), 1.88 – 1.95 (m, 1H), 2.42 – 2.48 (m, 2H), 3.51 – 3.62 (m, 2H), 3.93 – 4.00 (m, 2H), 4.12 – 4.22 (m, 1H), 5.56 (dd, *J* = 9.6, 5.4 Hz, 1H), 7.14 – 7.19 (m, 1H), 7.22 – 7.36 (m, 18H), 7.50 (s, 1H), 7.67 – 7.70 (m, 2H). ^{13}C NMR (125 MHz) δ 25.6, 25.8, 26.2, 28.3, 32.5, 33.1, 33.1, 33.3, 38.0, 40.7, 44.6, 47.8, 50.0, 66.4, 78.5, 114.8, 124.3, 126.3, 127.0, 128.2, 128.6, 129.2, 134.5, 139.7, 144.4, 144.6, 155.0, 169.7. LC-MS (ESI) (MeOH; method 2), RT 12.33 min, 98% purity, *m/z* = 741.5 ([M+H]$^+$).

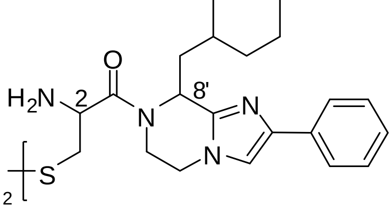

1 2*R*, 8'*S*
2 2*S*, 8'*S*
3 2*R*, 8'*R*
4 2*S*, 8'*R*

*(S,2R,2'R)-3,3'-Disulfanediylbis(2-amino-1-((S)-8-(cyclohexylmethyl)-2-phenyl-5,6-dihydroimidazo[1,2-a]pyrazin-7(8H)-yl)propan-1-one) Tetrahydrochloride (**1**). Typical Procedure to Deprotected Cystine Derivatives **1-4**.* Compound **76** (556 mg, 0.75 mmol) was dissolved in anhyd CH_2Cl_2 (6 mL) under argon atm and a mixture of triisopropylsilane (1.56 mL, 1.18 g, 7.50 mmol) and TFA (6 mL) was added. After stirring at room temperature for 18 h, the solvent was evaporated to dryness without heating. The residue was treated with *n*-hexane (30 mL), followed by decanting the organic phase. The remaining oil was dissolved in

anhyd EtOAc (20 mL) and treated with 1 M HCl in EtOAc (3 mL). The precipitate was filtered off, washed with anhyd EtOAc (2 × 25 mL) and dried *in vacuo*. The crude salt was dissolved in MeOH (25 mL) containing H_2O (0.5 mL). A solution of iodine (190 mg, 0.75 mmol) in MeOH (25 mL) was added dropwise under vigorous stirring and the reaction mixture was stirred at room temperature for 2 h. After evaporating the solvent *in vacuo*, the crude residue was purified by means of preparative CC using $CHCl_3$ / 7 N NH_3 in MeOH (100:5). The isolated yellowish oil was redissolved in anhyd EtOAc (20 mL) and treated with 1 M HCl in EtOAc (3 mL). The precipitate was filtered off, washed with anhyd EtOAc (2 × 25 mL) and dried *in vacuo* to obtain compound **1** as a yellowish solid (254 mg, 72%); mp > 240 °C decomp (lit.[5] mp 240–241 °C). ^{1}H NMR (500 MHz) δ 0.87 – 1.05 (m, 4H), 1.08 – 1.17 (m, 2H), 1.18 – 1.33 (m, 4H), 1.40 – 1.49 (m, 2H), 1.55 – 1.64 (m, 8H), 1.83 – 1.94 (m, 4H), 2.00 – 2.08 (m, 2H), 3.26 (dd, *J* = 14.8, 5.8 Hz, 1H), 3.31 (dd, *J* = 14.7, 5.9 Hz, 1H), 3.90 (ddd, *J* = 15.7, 11.5, 4.5 Hz, 2H), 4.16 – 4.26 (m, 2H), 4.27 – 4.37 (m, 2H), 4.38 – 4.55 (m, 2H), 4.79 – 4.88 (m, 2H), 5.86 (dd, *J* = 10.1, 4.7 Hz, 2H), 7.38 (t, *J* = 7.4 Hz, 2H), 7.47 (t, *J* = 7.7 Hz, 4H), 7.86 (d, *J* = 7.7 Hz, 4H), 8.01 (s, 2H), 8.77 (s, 6H). ^{13}C NMR (125 MHz)♦ δ 25.4, 25.7, 26.3, 32.1, 32.5, 33.6, 38.1, 38.5, 45.4, 47.1, 48.9, 116.7, 125.3, 128.5, 128.9, 129.2, 134.2, 144.0, 166.7. LC-MS (ESI) (MeOH; method 1), RT 12.69 min, 100% purity, *m/z* = 795.4 ([M+H]$^+$). HRMS, calcd. for $C_{44}H_{58}N_8O_2S_2$: [M+H]$^+$ *m/z* 795.4197; found: 795.4208.

*(S,2S,2'S)-3,3'-Disulfanediylbis(2-amino-1-((S)-8-(cyclohexylmethyl)-2-phenyl-5,6-dihydroimidazo[1,2-a]pyrazin-7(8H)-yl)propan-1-one) Tetrahydrochloride (**2**).* From **77**. CC using $CHCl_3$ / 7 N NH_3 in MeOH (100:5). Yield 155 mg, 44%. Orange solid; mp > 246 °C decomp. ^{1}H NMR (500 MHz) δ 0.92 – 1.06 (m, 4H), 1.06 – 1.25 (m, 6H), 1.27 – 1.35 (m, 2H), 1.51 – 1.73 (m, 8H), 1.95 – 2.11 (m, 6H), 3.36 (dd, *J* = 13.9, 7.0 Hz, 2H), 3.61 (dd, *J* = 13.9, 5.0 Hz, 2H), 3.89 (ddd, *J* = 15.6, 11.8, 4.3 Hz, 2H), 4.21 – 4.30 (m, 2H), 4.46 – 4.57 (m, 2H), 4.64 – 4.75 (m, 2H), 4.95 – 5.05 (m, 2H), 6.11 (dd, *J* = 11.5, 4.1 Hz, 2H), 7.40 – 7.46 (m, 2H), 7.47 – 7.54 (m, 4H), 7.89 – 7.96 (m, 4H), 8.20 (s, 2H), 8.93 (s, 6H). ^{13}C NMR (125 MHz)♦ δ 25.6, 26.1, 26.2, 31.4, 33.2, 33.7, 37.9, 38.1, 45.8, 46.1, 48.4, 117.3, 125.5, 127.0, 129.3, 129.4, 132.7, 143.7, 167.9. LC-MS (ESI) (MeOH; method 2), RT 10.02 min, 98% purity, *m/z* = 796.1 ([M+H]$^+$). HRMS, calcd. for $C_{44}H_{58}N_8O_2S_2$: [M+H]$^+$ *m/z* 795.4197; found: 795.4192.

*(R,2R,2'R)-3,3'-Disulfanediylbis(2-amino-1-((R)-8-(cyclohexylmethyl)-2-phenyl-5,6-dihydroimidazo[1,2-a]pyrazin-7(8H)-yl)propan-1-one) Tetrahydrochloride (**3**).* From **78**. CC using $CHCl_3$ / 7 N NH_3 in MeOH (100:5). Yield 201 mg, 57%. Yellowish solid (164 mg,

58%); mp > 246 °C decomp. ^{1}H NMR (500 MHz) δ 0.91 – 1.10 (m, 4H), 1.10 – 1.24 (m, 6H), 1.27 – 1.38 (m, 2H), 1.50 – 1.72 (m, 8H), 1.96 – 2.10 (m, 6H), 3.36 (dd, *J* = 13.9, 6.9 Hz, 2H), 3.61 (dd, *J* = 13.9, 5.0 Hz, 2H), 3.88 (ddd, *J* = 15.6, 11.5, 4.2 Hz, 2H), 4.20 – 4.31 (m, 2H), 4.46 – 4.56 (m, 2H), 4.63 – 4.74 (m, 2H), 4.95 – 5.06 (m, 2H), 6.10 (dd, *J* = 11.4, 4.1 Hz, 2H), 7.39 – 7.46 (m, 2H), 7.50 (dd, *J* = 8.4, 6.9 Hz, 4H), 7.87 – 7.97 (m, 4H), 8.19 (s, 2H), 8.93 (s, 6H). ^{13}C NMR (125 MHz)♦ δ 25.6, 26.1, 26.2, 31.4, 33.2, 33.7, 37.9, 38.1, 45.8, 46.2, 48.4, 117.2, 125.5, 127.1, 129.3,* 132.8, 143.7, 167.9. LC-MS (ESI) (MeOH; method 2), RT 11.70 min, 99% purity, *m/z* = 795.6 ([M+H]$^+$). HRMS, calcd. for $C_{44}H_{58}N_8O_2S_2$: [M+H]$^+$ *m/z* 795.4197; found: 795.4217.

*(R,2S,2'S)-3,3'-Disulfanediylbis(2-amino-1-((R)-8-(cyclohexylmethyl)-2-phenyl-5,6-dihydroimidazo[1,2-a]pyrazin-7(8H)-yl)propan-1-one) Tetrahydrochloride (**4**).* From **79**. CC using $CHCl_3$ / 7 N NH_3 in MeOH (100:5). Yield 159 mg, 45%. Yellow solid; mp > 240 °C decomp. ^{1}H NMR (500 MHz) δ 0.86 – 1.05 (m, 4H), 1.06 – 1.34 (m, 6H), 1.40 – 1.50 (m, 2H), 1.52 – 1.66 (m, 8H), 1.88 – 2.00 (m, 4H), 2.03 – 2.12 (m, 2H), 3.28 (dd, *J* = 14.7, 5.8 Hz, 2H), 3.35 (dd, *J* = 14.7, 5.7 Hz, 2H), 3.86 – 3.97 (m, 2H), 4.19 – 4.28 (m, 2H), 4.30 – 4.38 (m, 2H), 4.44 – 4.55 (m, 2H), 4.81 – 4.91 (m, 2H), 5.93 (dd, *J* = 10.5, 5.1 Hz, 2H), 7.40 (t, *J* = 7.4 Hz, 2H), 7.48 (t, *J* = 7.6 Hz, 4H), 7.92 (d, *J* = 7.6 Hz, 4H), 8.12 (s, 2H), 8.86 (s, 6H). ^{13}C NMR (125 MHz)♦ δ 25.3, 25.7, 26.3, 31.9, 32.4, 33.7, 38.0, 38.4, 45.6, 46.8, 48.8, 117.0, 125.5, 127.6, 129.3,* 133.2, 144.0, 166.8. LC-MS (ESI) (MeOH; method 2), RT 11.37 min, 96% purity, *m/z* = 795.6 ([M+H]$^+$). HRMS, calcd. for $C_{44}H_{58}N_8O_2S_2$: [M+H]$^+$ *m/z* 795.4197; found: 795.4147.

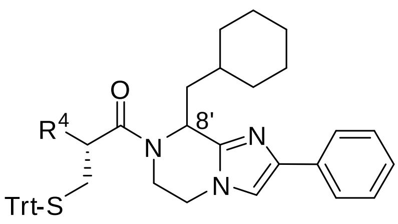

I (**80**-**83**)

*(S)-1-(8-(Cyclohexylmethyl)-2-phenyl-5,6-dihydroimidazo[1,2-a]pyrazin-7(8H)-yl)-3-(tritylthio)propan-1-one (**I**, 8'S, R^4 = H; **80**). Typical Procedure to Protected 3-Mercaptopropanoic Acid Derivatives **I**.* 3-(Tritylthio)propanoic acid (1.05 g, 3.00 mmol) and DCC (619 mg, 3.00 mmol) were stirred in anhyd CH_2Cl_2 (19 mL) under argon atm for 30 min. Compound **66** (295 mg, 1.00 mmol) dissolved in anhyd CH_2Cl_2 (12 mL) and DIPEA (0.17 mL, 129 mg, 1.00 mmol) were added and the reaction mixture was stirred at room temperature and argon atm for 48 h. After removing the solvent *in vacuo*, the residue was

treated with a mixture of petroleum ether/EtOAc (7:3) (80 mL). The white urea was filtered off, the filtrate was evaporated to dryness and the crude oily residue was purified by preparative CC on silica gel using a gradient of petroleum ether/EtOAc (4:1) to (7:3) to obtain **80** as yellow oil (394 mg, 63%). ^{1}H NMR (500 MHz) δ 0.79 – 0.96 (m, 2H), 1.09 – 1.29 (m, 3H), 1.49 – 1.73 (m, 7H), 1.92 – 1.98 (m, 1H), 2.29 – 2.47 (m, 4H), 3.49 – 3.58 (m, 1H), 3.81 – 3.88 (m, 1H), 3.88 – 3.94 (m, 1H), 3.94 – 4.01 (m, 1H), 5.63 (dd, *J* = 9.8, 5.4 Hz, 1H), 7.14 – 7.19 (m, 1H), 7.19 – 7.38 (m, 17H), 7.49 (s, 1H), 7.67 – 7.70 (m, 2H). ^{13}C NMR (125 MHz) δ 25.8, 25.9, 26.2, 27.3, 31.7, 32.4, 33.3, 33.5, 38.1, 40.9, 44.3, 47.2, 66.3, 80.6, 114.8, 124.3, 126.3, 126.8, 128.1, 128.5, 129.3, 134.6, 139.6, 144.7, 144.8, 169.7. LC-MS (ESI) (MeOH; method 1), RT 11.47 min, 99% purity, *m/z* = 626.4 ([M+H]$^{+}$).

*(R)-1-(8-(Cyclohexylmethyl)-2-phenyl-5,6-dihydroimidazo[1,2-a]pyrazin-7(8H)-yl)-3-(tritylthio)propan-1-one (**I**, 8'R, R^4 = H; **81**).* From **67** and 3-(tritylthio)propanoic acid. CC using a gradient of petroleum ether/EtOAc (4:1) to (7:3). Yield 401 mg, 64%. Yellow oil. ^{1}H NMR (500 MHz) δ 0.76 – 0.99 (m, 2H), 1.04 – 1.37 (m, 3H), 1.48 – 1.74 (m, 7H), 1.92 – 1.97 (m, 1H), 2.28 – 2.47 (m, 4H), 3.53 (ddd, *J* = 15.3, 11.5, 4.3 Hz, 1H), 3.82 – 3.88 (m, 1H), 3.88 – 3.95 (m, 1H), 3.96 – 4.01 (m, 1H), 5.63 (dd, *J* = 9.8, 5.3 Hz, 1H), 7.14 – 7.20 (m, 1H), 7.22 – 7.26 (m, 3H), 7.27 – 7.37 (m, 14H), 7.50 (s, 1H), 7.68 (dd, *J* = 8.3, 1.4 Hz, 2H). ^{13}C NMR (125 MHz) δ 25.8, 25.9, 26.2, 27.2, 31.7, 32.4, 33.3, 33.5, 38.1, 40.8, 44.3, 47.2, 66.3, 114.8, 124.3, 126.4, 126.8, 128.1, 128.6, 129.3, 134.4, 139.5, 144.7, 144.8, 169.7. LC-MS (ESI) (MeOH; method 1), RT 13.57 min, 97% purity, *m/z* = 626.5 ([M+H]$^{+}$).

*(9H-Fluoren-9-yl)methyl (R)-1-((S)-8-(Cyclohexylmethyl)-2-phenyl-5,6-dihydroimidazo[1,2-a]pyrazin-7(8H)-yl)-1-oxo-3-(tritylthio)propan-2-ylcarbamate (**I**, 8'S, R^4 = NH-Fmoc; **82**).* From **66** and Fmoc-L-Cys(Trt)-OH (1.76 g). CC using a gradient of petroleum ether/EtOAc (4:1) to (7:3). Yield 587 mg, 68%. Yellow oil. ^{1}H NMR (600 MHz)$^{\blacklozenge}$ δ 0.76 – 0.89 (m, 2H), 0.97 – 1.12 (m, 2H), 1.20 – 1.32 (m, 1H), 1.38 – 1.63 (m, 7H), 1.63 – 1.73 (m, 1H), 1.80 – 1.88 (m, 1H), 2.53 – 2.62 (m, 1H), 3.51 (ddd, *J* = 15.6, 11.8, 4.0 Hz, 1H), 3.60 – 3.71 (m, 1H), 3.84 – 3.94 (m, 1H), 3.96 – 4.01 (m, 1H), 4.16 – 4.25 (m, 2H), 4.26 – 4.30 (m, 2H), 5.53 – 5.59 (m, 1H), 7.17 (tt, *J* = 7.2, 1.3 Hz, 1H), 7.19 – 7.45 (m, 21H), 7.50 (s, 1H), 7.66 – 7.72 (m, 4H), 7.87 (d, *J* = 7.6 Hz, 2H), 7.99 (d, *J* = 8.6 Hz, 1H). ^{13}C NMR (150 MHz) δ 25.7, 25.9, 26.2, 32.6, 33.2, 33.2, 33.2, 38.1, 40.7, 44.6, 46.8, 47.7, 50.3, 66.0, 66.4, 114.8, 120.3, 124.4, 125.4, 125.4, 126.4, 127.0, 127.2, 127.2, 127.8, 127.8, 128.3, 128.6, 129.3, 134.5, 139.8, 140.9, 143.8, 143.9, 144.5,* 155.7, 169.3, four aromatic carbons gave two signals each. LC-MS (ESI) (MeOH; method 2), RT 12.97 min, 94% purity, *m/z* = 863.2 ([M+H]$^{+}$).

*N-((R)-1-((S)-8-(Cyclohexylmethyl)-2-phenyl-5,6-dihydroimidazo[1,2-a]pyrazin-7(8H)-yl)-1-oxo-3-(tritylthio)propan-2-yl)acetamide (**I**, 8'S, R^4 = NH-Ac; **83**).* From **66** and Ac-L-Cys(Trt)-OH (1.22 g). Ac-L-Cys(Trt)-OH (1.22 g, 3.00 mmol) and HATU (1.14 g, 3.00 mmol) were stirred in anhyd DMF (12.5 mL) under argon atm for 30 min. Compound **66** (295 mg, 1.00 mmol) dissolved in anhyd DMF (5 mL) and DIPEA (0.17 mL, 129 mg, 1.00 mmol) were added and the reaction mixture was stirred at room temperature and argon atm for 48 h. After removing the solvent *in vacuo*, the residue was purified by preparative CC on silica gel using petroleum ether/EtOAc (1:1) to obtain **83** as yellow oil (396 mg, 29%). ^{1}H NMR (500 MHz) δ 0.80 – 0.88 (m, 2H), 1.05 – 1.19 (m, 3H), 1.23 (s, 3H), 1.54 – 1.73 (m, 7H), 1.82 – 1.92 (m, 1H), 2.41 (dd, *J* = 12.3, 7.6 Hz, 1H), 2.50 – 2.55 (m, 1H), 3.54 (ddd, *J* = 15.6, 11.9, 4.0 Hz, 1H), 3.65 – 3.73 (m, 1H), 3.96 – 4.06 (m, 2H), 4.61 (app q, *J* = 7.7 Hz, 1H), 5.56 (app t, *J* = 7.6 Hz, 1H), 7.14 – 7.19 (m, 1H), 7.21 – 7.37 (m, 17H), 7.51 (s, 1H), 7.66 – 7.71 (m, 2H), 8.37 (d, *J* = 8.9 Hz, 1H). ^{13}C NMR (125 MHz) δ 22.3, 25.8, 25.9, 26.2, 32.7, 33.1, 33.4, 33.7, 38.2, 40.9, 44.7, 47.8, 47.9, 66.3, 114.8, 124.4, 126.4, 127.0, 128.2, 128.6, 129.2, 134.5, 139.8, 144.4, 144.5, 168.8, 169.3. LC-MS (ESI) (MeOH; method 1), RT 13.13 min, 94% purity, *m/z* = 683.5 ([M+H]$^+$).

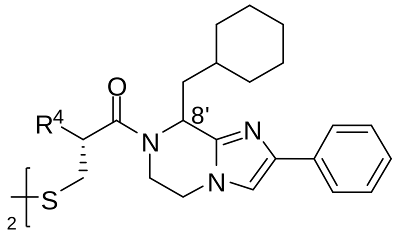

5 8'*S*, R^4 = H
6 8'*R*, R^4 = H
7 8'*S*, R^4 = NH-Fmoc
8 8'*S*, R^4 = NH-Ac

*(S)-3,3'-Disulfanediylbis(1-((S)-8-(cyclohexylmethyl)-2-phenyl-5,6-dihydroimidazo[1,2-a]pyrazin-7(8H)-yl)propan-1-one) Dihydrochloride (**5**). Typical Procedure to Disulfanediyldipropanoic Acid Derivatives **5-8**.* Compound **80** (313 mg, 0.50 mmol) was dissolved in anhyd CH_2Cl_2 (4 mL) under argon atm and a mixture of triisopropylsilane (1.04 mL, 792 mg, 5.00 mmol) and TFA (2 mL) was added. After stirring at room temperature for 18 h, the solvent was evaporated to dryness without heating. The residue was treated with *n*-hexane (20 mL), followed by decanting the organic phase. The remaining oil was dissolved in MeOH (13.5 mL) and a solution of iodine (127 mg, 0.50 mmol) in MeOH (13.5 mL) was added dropwise under vigorous stirring. The reaction mixture was stirred at room temperature for 2 h. After evaporating the solvent *in vacuo*, the crude residue was purified by means of preparative CC using a gradient of CH_2Cl_2/EtOAc (4:1) to (7:3). The resulting oil was dissolved in MeOH/MeCN (1:1) (52.5 mg/mL) and purified through semi-preparative HPLC with a gradient from 30% MeCN + 0.05% TFA / 70% H_2O + 0.05% TFA to 90% MeCN +

0.05% TFA / 10% H_2O + 0.05% TFA within 15 min (200 µL; 14.0 mL/min; 255 nm), followed by volatile evaporation and lyophilization. The residue was redissolved in anhyd EtOAc (15 mL), treated with 1 M HCl in EtOAc (1 mL) and evaporated to dryness. The remaining salt was dried *in vacuo* to obtain compound **5** as a yellowish solid (16.8 mg, 8%); mp > 212 °C decomp. ^{1}H NMR (500 MHz) δ 0.82 – 1.03 (m, 4H), 1.08 – 1.20 (m, 6H), 1.21 – 1.31 (m, 2H), 1.53 – 1.65 (m, 8H), 1.83 – 1.95 (m, 4H), 1.98 – 2.04 (m, 2H), 2.80 – 3.11 (m, 8H), 3.71 – 3.80 (m, 2H), 4.13 – 4.27 (m, 4H), 4.29 – 4.39 (m, 2H), 6.00 (dd, *J* = 11.0, 4.3 Hz, 2H), 7.41 (t, *J* = 7.5 Hz, 2H), 7.49 (t, *J* = 7.6 Hz, 4H), 7.83 (d, *J* = 7.7 Hz, 4H), 8.07 (s, 2H). ^{13}C NMR (125 MHz)♦ δ 25.6, 26.0, 26.1, 31.7, 32.2, 33.4, 33.5, 33.7, 37.5, 45.4, 45.6, 117.0, 125.3, 127.8, 129.3,* 133.1, 144.6, 170.4. LC-MS (ESI) (MeOH; method 1), RT 13.19 min, 99% purity, *m/z* = 765.5 ([M+H]$^+$). HRMS, calcd. for $C_{44}H_{56}N_6O_2S_2$: [M+H]$^+$ *m/z* 765.3979; found: 765.3993.

*(R)-3,3'-Disulfanediylbis(1-((R)-8-(cyclohexylmethyl)-2-phenyl-5,6-dihydroimidazo[1,2-a]pyrazin-7(8H)-yl)propan-1-one) Dihydrochloride (**6**).* From **81**. CC using a gradient of CH_2Cl_2/EtOAc (4:1) to (7:3). The oil in MeOH/MeCN (1:1) (52.5 mg/mL) was purified through semi-preparative HPLC with a gradient from 30% MeCN + 0.05% TFA / 70% H_2O + 0.05% TFA to 90% MeCN + 0.05% TFA / 10% H_2O + 0.05% TFA within 15 min (200 µL; 14.0 mL/min; 255 nm). Yield 27.2 mg, 13%. Yellowish solid; mp > 212 °C decomp. ^{1}H NMR (600 MHz) δ 0.89 – 1.10 (m, 4H), 1.05 – 1.19 (m, 6H), 1.20 – 1.29 (m, 2H), 1.52 – 1.71 (m, 8H), 1.82 (ddd, *J* = 14.1, 9.7, 3.9 Hz, 2H), 1.87 – 1.96 (m, 2H), 1.99 – 2.07 (m, 2H), 2.82 – 3.06 (m, 8H), 3.75 (ddd, *J* = 15.5, 9.9, 6.2 Hz, 2H), 4.13 – 4.26 (m, 4H), 4.27 – 4.38 (m, 2H), 5.99 (dd, *J* = 11.4, 4.0 Hz, 2H), 7.41 (t, *J* = 7.4 Hz, 2H), 7.49 (t, *J* = 7.6 Hz, 4H), 7.81 (d, *J* = 7.8 Hz, 4H), 8.06 (s, 2H). ^{13}C NMR (150 MHz)♦ δ 25.7, 26.0, 26.2, 31.8, 32.2, 33.4, 33.6, 33.7, 37.5, 45.5, 45.6, 117.1, 125.3, 127.8, 129.4,* 133.2, 144.6, 170.5. LC-MS (ESI) (MeOH; method 1), RT 12.27 min, 97% purity, *m/z* = 765.5 ([M+H]$^+$). HRMS, calcd. for $C_{44}H_{56}N_6O_2S_2$: [M+H]$^+$ *m/z* 765.3979; found: 765.3981.

*Bis((9H-fluoren-9-yl)methyl) (S,2R,2'R)-3,3'-Disulfanediylbis(1-((S)-8-(cyclohexylmethyl)-2-phenyl-5,6-dihydroimidazo[1,2-a]pyrazin-7(8H)-yl)-1-oxopropane-3,2-diyl)dicarbamate Dihydrochloride (**7**).* From **82** (432 mg). CC using a gradient of CH_2Cl_2/EtOAc (9:1) to (3:2). The oil in MeOH/MeCN (1:1) (30.0 mg/mL) was purified through semi-preparative HPLC with a gradient from 70% MeCN + 0.05% TFA / 30% H_2O + 0.05% TFA to 90% MeCN + 0.05% TFA / 10% H_2O + 0.05% TFA within 17 min (400 µL; 14.0 mL/min; 260 nm). Yield 26.3 mg, 8%. Yellowish solid; mp > 230 °C decomp. ^{1}H NMR (500 MHz) δ 0.80 – 1.29 (m, 10H), 1.35 – 1.62 (m, 10H), 1.79 – 2.04 (m, 6H), 3.02 (dd, *J* = 13.7, 8.2 Hz, 2H), 3.15 (dd, *J* =

13.9, 5.4 Hz, 2H), 4.13 – 4.39 (m, 10H), 4.45 – 4.58 (m, 2H), 4.81 (app q, *J* = 7.6 Hz, 2H), 5.92 – 6.03 (m, 2H), 7.30 (t, *J* = 7.4 Hz, 4H), 7.35 – 7.44 (m, 6H), 7.47 (t, *J* = 7.6 Hz, 4H), 7.66 (d, *J* = 7.6 Hz, 4H), 7.80 (d, *J* = 7.7 Hz, 4H), 7.87 (d, ^3J = 7.6 Hz, 4H), 7.98 (s, 2H), 8.15 (d, *J* = 8.6 Hz, 2H). One proton signal is obscured by the water peak. ^{13}C NMR (125 MHz)♦♦δ 25.5, 25.9, 26.1, 31.9, 33.0, 33.5, 37.5, 45.6, 46.0, 46.8, 50.2, 66.1, 116.8, 120.3, 125.2, 125.3, 125.3, 127.1, 127.2, 127.8,* 129.3,* 133.6, 140.9, 143.8, 144.4, 156.1, 169.9. Two aromatic carbons gave two signals each. LC-MS (ESI) (MeOH; method 1), RT 14.94 min, 99% purity, *m/z* = 1240.0 ([M+H]$^+$). HRMS, calcd. for $C_{74}H_{78}N_8O_6S_2$: [M+Na]$^+$ *m/z* 1261.5378; found: 1261.5333.

*N,N'-(S,2R,2'R)-3,3'-Disulfanediylbis(1-((S)-8-(cyclohexylmethyl)-2-phenyl-5,6-dihydroimidazo[1,2-a]pyrazin-7(8H)-yl)-1-oxopropane-3,2-diyl)diacetamide Dihydrochloride (**8**).* From **83** (341 mg). CC using CH_2Cl_2/MeOH (19:1). The oil in MeOH/MeCN (1:1) (49.6 mg/mL) was purified through semi-preparative HPLC with a gradient from 20% MeCN + 0.05% TFA / 80% H_2O + 0.05% TFA to 90% MeCN + 0.05% TFA / 10% H_2O + 0.05% TFA within 15 min (200 µL; 14.0 mL/min; 255 nm). Yield 23.8 mg, 10%. Yellowish solid; mp > 218 °C decomp. ^{1}H NMR (600 MHz) δ 0.82 – 1.03 (m, 4H), 1.07 – 1.26 (m, 6H), 1.49 – 1.68 (m, 10H), 1.86 (s, 6H), 1.87 – 2.04 (m, 6H), 2.90 – 2.97 (m, 2H), 3.14 (dd, *J* = 13.6, 6.1 Hz, 2H), 3.79 (ddd, *J* = 16.0, 11.8, 4.4 Hz, 2H), 4.13 – 4.23 (m, 2H), 4.27 – 4.36 (m, 2H), 4.55 – 4.66 (m, 2H), 5.10 (app q, *J* = 7.5 Hz, 2H), 5.98 (dd, *J* = 11.2, 4.6 Hz, 2H), 7.41 (t, *J* = 7.4 Hz, 2H), 7.49 (t, *J* = 7.5 Hz, 4H), 7.80 (d, *J* = 7.6 Hz, 4H), 8.08 (s, 2H), 8.63 (d, *J* = 8.3 Hz, 2H). ^{13}C NMR (150 MHz)♦♦δ 22.3, 25.7, 25.9, 26.1, 32.0, 33.3, 33.5, 37.7, 45.9, 46.0, 47.9, 117.1, 125.4, 127.5, 129.3,* 133.1, 144.3, 169.4, 170.0. LC-MS (ESI) (MeOH; method 1), RT 10.65 min, 99% purity, *m/z* = 879.4 ([M+H]$^+$). HRMS, calcd. for $C_{48}H_{62}N_8O_4S_2$: [M+H]$^+$ *m/z* 879.4408; found: 879.4421.

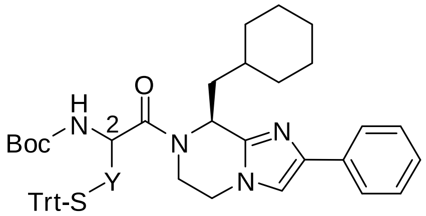

K (**84**-**86**)

*tert-Butyl (R)-1-((S)-8-(Cyclohexylmethyl)-2-phenyl-5,6-dihydroimidazo[1,2-a]pyrazin-7(8H)-yl)-3-methyl-1-oxo-3-(tritylthio)butan-2-ylcarbamate (**K**, 2R, Y = C(CH$_3$)$_2$; **84**). Typical Procedure to Protected Mercaptobutanoic Acid Derivatives **K**.* Boc-L-Pen(Trt)-OH (2.21 g, 4.50 mmol) and DCC (928 mg, 4.50 mmol) were stirred in anhyd CH_2Cl_2 (28 mL)

under argon atm for 30 min. Compound **66** (443 mg, 1.50 mmol) dissolved in anhyd CH_2Cl_2 (18 mL) and DIPEA (0.26 mL, 194 mg, 1.50 mmol) were added and the reaction mixture was stirred at room temperature and argon atm for 48 h. After removing the solvent *in vacuo,* the residue was treated with a mixture of petroleum ether/EtOAc (7:3) (175 mL). The white urea was filtered off, the filtrate was evaporated to dryness and the crude oily residue was purified by preparative CC on silica gel using petroleum ether/EtOAc (4:1) to obtain **84** as yellowish oil (404 mg, 35%). ^{1}H NMR (500 MHz) δ 0.76 – 0.91 (m, 2H), 0.93 (s, 3H), 1.01 (s, 3H), 1.12 – 1.30 (m, 3H), 1.42 (s, 9H), 1.50 – 1.84 (m, 7H), 1.90 – 1.97 (m, 1H), 3.64 – 3.76 (m, 1H), 3.96 – 4.08 (m, 1H), 4.09 – 4.17 (m, 1H), 4.22 – 4.33 (m, 1H), 4.52 (d, *J* = 9.1 Hz, 1H), 5.71 (dd, *J* = 8.9, 5.6 Hz, 1H), 7.04 (d, *J* = 8.9 Hz, 1H), 7.17 – 7.25 (m, 4H), 7.26 – 7.33 (m, 6H), 7.35 (t, *J* = 7.8 Hz, 2H), 7.41 – 7.57 (m, 6H), 7.58 (s, 1H), 7.70 – 7.74 (m, 2H). ^{13}C NMR (125 MHz) δ 24.3, 25.7, 25.8, 26.2, 26.8, 28.3, 32.7, 33.1, 33.3, 38.6, 40.5, 44.3, 47.6, 54.0, 56.2, 68.0, 78.7, 114.9, 124.4, 126.5, 126.7, 127.8, 128.6, 129.8, 134.2, 139.6, 144.7, 144.8, 155.7, 169.6. LC-MS (ESI) (MeOH; method 2), RT 12.70 min, 97% purity, *m/z* = 769.2 ([M+H]$^+$).

*tert-Butyl (S)-1-((S)-8-(Cyclohexylmethyl)-2-phenyl-5,6-dihydroimidazo[1,2-a]pyrazin-7(8H)-yl)-3-methyl-1-oxo-3-(tritylthio)butan-2-ylcarbamate (**K**, 2S, Y = C(CH_3)$_2$; **85**).* From **66** and Boc-D-Pen(Trt)-OH (2.21 g). CC using petroleum ether/EtOAc (4:1). Yield 404 mg, 35%. Yellow oil. ^{1}H NMR (500 MHz) δ 0.77 – 0.89 (m, 2H), 0.95 (s, 3H), 0.98 – 1.09 (m, 2H), 1.13 (s, 3H), 1.20 – 1.29 (m, 1H), 1.33 (s, 9H), 1.40 – 1.82 (m, 7H), 2.00 – 2.07 (m, 1H), 3.57 – 3.70 (m, 1H), 3.75 – 3.82 (m, 1H), 3.88 – 3.96 (m, 1H), 3.99 – 4.07 (m, 1H), 4.24 (d, *J* = 9.2 Hz, 1H), 5.68 – 5.75 (m, 1H), 7.09 – 7.12 (m, 1H), 7.14 – 7.18 (m, 1H), 7.20 – 7.24 (m, 3H), 7.28 – 7.34 (m, 8H), 7.51 – 7.56 (m, 7H), 7.67 – 7.70 (m, 2H). ^{13}C NMR (125 MHz) δ 25.2, 25.6, 25.8, 26.1, 26.2, 28.2, 32.1, 33.4, 33.6, 38.9, 40.9, 44.3, 47.3, 53.9, 56.5, 67.8, 78.7, 114.8, 124.3, 126.3, 126.6, 127.8, 128.5, 129.8, 134.5, 139.8, 144.9, 145.1, 155.3, 168.7. LC-MS (ESI) (MeOH; method 2), RT 12.24 min, 95% purity, *m/z* = 769.2 ([M+H]$^+$).

*tert-Butyl (S)-1-((S)-8-(Cyclohexylmethyl)-2-phenyl-5,6-dihydroimidazo[1,2-a]pyrazin-7(8H)-yl)-1-oxo-4-(tritylthio)butan-2-ylcarbamate (**K**, 2S, Y = CH_2CH_2; **86**).* From **66** and Boc-L-Hcy(Trt)-OH (2.15 g). CC using a gradient of petroleum ether/EtOAc (4:1) to (7:3). Yield 464 mg, 41%. Colorless oil. ^{1}H NMR (500 MHz) δ 0.77 – 0.95 (m, 2H), 1.09 – 1.25 (m, 3H), 1.37 (s, 9H), 1.52 – 1.71 (m, 9H), 1.94 – 2.05 (m, 1H), 2.02 – 2.09 (m, 1H), 2.12 (dt, *J* = 11.4, 7.7 Hz, 1H), 3.58 – 3.68 (m, 1H), 3.71 – 3.81 (m, 1H), 4.02 – 4.08 (m, 1H), 4.22 – 4.37 (m, 1H), 4.46 (app q, *J* = 7.5 Hz, 1H), 5.55 (dd, *J* = 9.8, 5.3 Hz, 1H), 7.10 (d, *J* = 8.1 Hz, 1H), 7.13 – 7.37 (m, 18H), 7.49 (s, 1H), 7.66 – 7.74 (m, 2H). ^{13}C NMR (125 MHz) δ 25.7, 25.9,

26.2, 28.0, 28.3, 30.3, 32.6, 33.1, 33.3, 38.1, 40.8, 44.5, 47.8, 49.6, 66.2, 78.3, 114.8, 124.4, 126.3, 126.8, 128.1, 128.6, 129.1, 134.6, 139.8, 144.5, 144.8, 155.4, 170.5. LC-MS (ESI) (MeOH; method 2), RT 12.55 min, 100% purity, *m/z* = 755.7 ($[M+H]^+$).

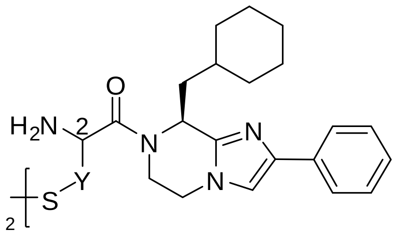

9 2*R*, Y = $C(CH_3)_2$
10 2*S*, Y = $C(CH_3)_2$
11 2*S*, Y = CH_2CH_2

*(S,2R,2'R)-3,3'-Disulfanediylbis(2-amino-1-((S)-8-(cyclohexylmethyl)-2-phenyl-5,6-dihydroimidazo[1,2-a]pyrazin-7(8H)-yl)-3-methylbutan-1-one) Tetrahydrochloride (**9**). Typical Procedure to Disulfanediyldibutanoic Acid Derivatives **9-11**.* Compound **84** (769 mg, 1.00 mmol) was dissolved in anhyd CH_2Cl_2 (8 mL) under argon atm and a mixture of triisopropylsilane (2.08 mL, 1.58 g, 10.0 mmol) and TFA (8 mL) was added. After stirring at room temperature for 18 h, the solvent was evaporated to dryness without heating. The residue was treated with *n*-hexane (40 mL), followed by decanting the organic phase. The remaining oil was dissolved in anhyd EtOAc (35 mL) and treated with 1 M HCl in EtOAc (4 mL). The precipitate was filtered off, washed with anhyd EtOAc (2 × 30 mL) and dried *in vacuo*. The crude salt was dissolved in MeOH (30 mL) containing H_2O (0.6 mL). A solution of iodine (254 mg, 1.00 mmol) in MeOH (30 mL) was added dropwise under vigorous stirring and the reaction mixture was stirred at room temperature for 2 h. After evaporating the solvent *in vacuo*, the crude residue was purified by means of preparative CC $CHCl_3$ / 7 N NH_3 in MeOH (100:2.5). The resulting oil was redissolved in anhyd EtOAc (35 mL) and treated with 1 M HCl in EtOAc (4 mL). The precipitate was filtered off, washed with anhyd EtOAc (2 × 30 mL) and dried *in vacuo*. The crude salt was dissolved in MeOH (100 mg/mL) and purified through semi-preparative HPLC with a gradient from 30% MeCN + 0.05% TFA / 70% H_2O + 0.05% TFA to 60% MeCN + 0.05% TFA / 40% H_2O + 0.05% TFA within 15 min (100 µL; 15.0 mL/min; 255 nm), followed by volatile evaporation and lyophilization. The residue was redissolved in anhyd EtOAc (35 mL) and treated with 1 M HCl in EtOAc (4 mL). The precipitate was again filtered off, washed with anhyd EtOAc (2 × 30 mL) and dried *in vacuo* to obtain compound **9** as a yellowish solid (189 mg, 38%); > mp 220 °C decomp. ^1H NMR (500 MHz) δ 0.83 – 1.25 (m, 10H), 1.26 – 1.72 (m, 20H), 1.73 – 1.97 (m, 8H), 3.77 – 3.97 (m, 2H), 3.99 – 4.21 (m, 2H), 4.22 – 4.33 (m, 2H), 4.47 – 4.66 (m, 4H), 5.66 – 5.90 (m, 2H), 7.28 – 7.37 (m, 2H), 7.38 – 7.53 (m, 4H), 7.73 – 7.96 (m, 6H), 8.77 (s, 6H). ^{13}C NMR

(125 MHz)♦♦δ 24.6, 25.7,** 26.3, 32.6, 32.7, 33.1, 44.6, 47.7, 51.1, 54.6, 116.3, 125.1, 128.3, 128.7, 129.1, 131.3, 144.2, 166.0. LC-MS (ESI) (MeOH; method 1), RT 12.86 min, 99% purity, *m/z* = 851.8 ($[M+H]^+$). HRMS, calcd. for $C_{48}H_{66}N_8O_2S_2$: $[M+H]^+$ *m/z* 851.4823; found: 851.4847.

*(S,2S,2'S)-3,3'-Disulfanediylbis(2-amino-1-((S)-8-(cyclohexylmethyl)-2-phenyl-5,6-dihydroimidazo[1,2-a]pyrazin-7(8H)-yl)-3-methylbutan-1-one) Tetrahydrochloride (**10**).* From **85**. CC using $CHCl_3$ / 7 N NH_3 in MeOH (100:2.5). After precipitation, the crude salt in MeOH (78 mg/mL) was purified through semi-preparative HPLC with a gradient from 30% MeCN + 0.05% TFA / 70% H_2O + 0.05% TFA to 60% MeCN + 0.05% TFA / 40% H_2O + 0.05% TFA within 15 min (200 µL; 14.0 mL/min; 255 nm). Yield 105 mg, 21%. Yellowish solid; mp > 224 °C decomp. ^{1}H NMR (600 MHz) δ 0.94 – 1.08 (m, 4H), 1.08 – 1.21 (m, 4H), 1.28 – 1.37 (m, 2H), 1.47 (s, 12H), 1.54 – 1.75 (m, 10H), 1.96 (ddd, *J* = 14.2, 10.3, 4.0 Hz, 2H), 2.00 – 2.06 (m, 2H), 2.06 – 2.11 (m, 2H), 3.87 (ddd, *J* = 15.6, 11.8, 4.3 Hz, 2H), 4.16 – 4.24 (m, 2H), 4.55 – 4.64 (m, 2H), 4.67 – 4.77 (m, 4H), 6.09 (dd, *J* = 11.5, 3.9 Hz, 2H), 7.41 (t, *J* = 7.4 Hz, 2H), 7.50 (t, *J* = 7.7 Hz, 4H), 7.85 (d, *J* = 7.8 Hz, 4H), 8.11 (s, 2H), 8.80 (s, 6H). ^{13}C NMR (150 MHz)♦δ 25.2, 25.3, 25.8, 26.2, 26.2, 31.4, 33.7, 33.8, 38.9, 45.5, 46.6, 51.2, 55.0, 117.1, 125.4, 127.8, 129.3, 129.4, 133.6, 143.8, 166.9. LC-MS (ESI) (MeOH; method 1), RT 13.21 min, 99% purity, *m/z* = 851.4 ($[M+H]^+$). HRMS, calcd. for $C_{48}H_{66}N_8O_2S_2$: $[M+H]^+$ *m/z* 851.4823; found: 851.4773.

*(S,2S,2'S)-4,4'-Disulfanediylbis(2-amino-1-((S)-8-(cyclohexylmethyl)-2-phenyl-5,6-dihydroimidazo[1,2-a]pyrazin-7(8H)-yl)butan-1-one) Tetrahydrochloride (**11**).* From **86** (755 mg). CC using a gradient of $CHCl_3$ / 7 N NH_3 in MeOH (100:2.5) to (100:5). After precipitation, the crude salt in MeOH (92 mg/mL) was purified through semi-preparative HPLC with a gradient from 20% MeCN + 0.05% TFA / 80% H_2O + 0.05% TFA to 50% MeCN + 0.05% TFA / 50% H_2O + 0.05% TFA within 15 min (200 µL; 14.0 mL/min; 255 nm). Yield 24.2 mg, 5%. Yellowish solid; mp > 230 °C decomp. ^{1}H NMR (500 MHz) δ 0.88 – 1.06 (m, 4H), 1.07 – 1.33 (m, 6H), 1.37 – 1.49 (m, 2H), 1.56 – 1.65 (m, 8H), 1.78 – 1.94 (m, 4H), 2.03 – 2.22 (m, 6H), 2.86 – 2.99 (m, 4H), 3.84 (ddd, *J* = 15.7, 11.7, 4.3 Hz, 2H), 4.03 – 4.12 (m, 2H), 4.24 – 4.32 (m, 2H), 4.34 – 4.45 (m, 2H), 4.58 – 4.65 (m, 2H), 5.83 (dd, *J* = 9.9, 4.6 Hz, 2H), 7.37 (t, *J* = 7.4 Hz, 2H), 7.47 (t, *J* = 7.7 Hz, 4H), 7.82 (d, *J* = 7.6 Hz, 4H), 7.99 (s, 2H), 8.56 (s, 6H). ^{13}C NMR (125 MHz)♦δ 25.4, 25.8, 26.3, 30.4, 31.7, 32.2, 32.6, 33.6, 38.0, 45.3, 47.1, 49.1, 116.6, 125.3, 126.2, 128.8, 129.3, 134.1, 144.5, 167.7. LC-MS (ESI) (MeOH; method 1), RT 12.70 min, 94% purity, *m/z* = 823.5 ($[M+H]^+$). HRMS, calcd. for $C_{46}H_{62}N_8O_2S_2$: $[M+H]^+$ *m/z* 823.4510; found: 823.4536.

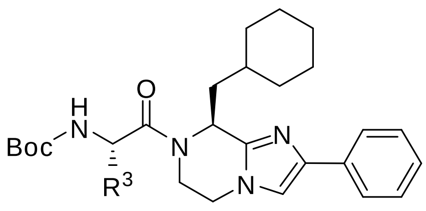

L (**87**-**93**)

*tert-Butyl (S)-1-((S)-8-(Cyclohexylmethyl)-2-phenyl-5,6-dihydroimidazo[1,2-a]pyrazin-7(8H)-yl)-1-oxobutan-2-ylcarbamate (**L**, R^3 = (S)-CH_2CH_3; **87**). Typical Procedure to Various N-Protected Amino Acid Derivatives **L**.* Boc-L-Abu-OH (610 mg, 3.00 mmol) and DCC (619 mg, 3.00 mmol) were stirred in anhyd CH_2Cl_2 (19 mL) under argon atm for 30 min. Compound **66** (295 mg, 1.00 mmol) dissolved in anhyd CH_2Cl_2 (12 mL) and DIPEA (0.17 mL, 129 mg, 1.00 mmol) were added and the reaction mixture was stirred at room temperature and argon atm for 48 h. After removing the solvent *in vacuo*, the residue was treated with a mixture of petroleum ether/EtOAc (7:3) (140 mL). The white urea was filtered off, the filtrate was evaporated to dryness and the crude oily residue was purified by preparative CC on silica gel using CH_2Cl_2/EtOAc (9.5:0.5) to obtain **87** as yellow oil (356 mg, 74%). ^{1}H NMR (500 MHz) δ 0.85 (t, *J* = 7.3 Hz, 3H), 0.87 – 0.94 (m, 1H), 0.95 – 1.08 (m, 1H), 1.09 – 1.27 (m, 3H), 1.39 (s, 9H), 1.48 – 1.73 (m, 9H), 1.94 – 2.05 (m, 1H), 3.64 – 3.72 (m, 1H), 3.90 – 3.99 (m, 1H), 4.07 – 4.14 (m, 1H), 4.33 – 4.43 (m, 2H), 5.64 (dd, *J* = 9.8, 5.2 Hz, 1H), 7.07 (d, *J* = 7.7 Hz, 1H), 7.14 – 7.19 (m, 1H), 7.32 (t, *J* = 7.7 Hz, 2H), 7.52 (s, 1H), 7.67 – 7.72 (m, 2H). ^{13}C NMR (125 MHz) δ 10.5, 24.5, 25.7, 25.9, 26.3, 28.4, 32.6, 33.2, 33.4, 38.0, 40.9, 44.8, 47.7, 52.0, 78.1, 114.8, 124.4, 126.3, 128.6, 134.6, 139.7, 144.9, 155.7, 171.5. LC-MS (ESI) (MeOH; method 1), RT 10.97 min, 98% purity, *m/z* = 481.2 ($[M+H]^+$).

*tert-Butyl (S)-1-((S)-8-(Cyclohexylmethyl)-2-phenyl-5,6-dihydroimidazo[1,2-a]pyrazin-7(8H)-yl)-1-oxo-3-(trityloxy)propan-2-ylcarbamate (**L**, R^3 = (S)-CH_2OTrt; **88**).* From compound **66** and Boc-L-Ser(Trt)-OH (1.34 g, 3.00 mmol). CC using petroleum ether/EtOAc (7:3). Yield 594 mg, 82%. Yellow oil. ^{1}H NMR (500 MHz) δ 0.78 – 0.87 (m, 1H), 0.89 – 0.98 (m, 1H), 1.09 – 1.31 (m, 3H), 1.40 (s, 9H), 1.41 – 1.46 (m, 1H), 1.54 – 1.68 (m, 5H), 1.68 – 1.80 (m, 1H), 1.95 – 2.05 (m, 1H), 3.13 (dd, *J* = 8.9, 6.1 Hz, 1H), 3.64 – 3.76 (m, 2H), 3.97 – 4.02 (m, 1H), 4.36 – 4.47 (m, 1H), 4.76 (app q, *J* = 6.9 Hz, 1H), 5.68 (dd, *J* = 9.4, 5.7 Hz, 1H), 7.15 – 7.41 (m, 19H), 7.47 (s, 1H), 7.71 – 7.74 (m, 2H). One proton signal is obscured by the water peak. ^{13}C NMR (125 MHz) δ 25.7, 25.8, 26.2, 28.3, 32.6, 33.2, 33.6, 38.2, 40.9, 44.5, 47.9, 50.1, 63.5, 78.5, 86.2, 114.9, 124.3, 126.3, 127.1, 127.9, 128.3, 128.6, 134.6,

139.7, 143.6, 144.8, 155.3, 169.7. LC-MS (ESI) (MeOH; method 2), RT 12.46 min, 100% purity, *m/z* = 725.9 ($[M+H]^+$).

*tert-Butyl (S)-3-((S)-8-(Cyclohexylmethyl)-2-phenyl-5,6-dihydroimidazo[1,2-a]pyrazin-7(8H)-yl)-3-oxopropane-1,2-diyldicarbamate (**L**, R^3 = (S)-CH_2NH_2; **89**).* Boc-L-Dap-OH (817 mg, 4.00 mmol) and $NaHCO_3$ (1.68 g, 20.0 mmol) were dissolved in H_2O (20 mL) and 1,4-dioxane (20 mL). Di-*tert*-butyl dicarbonate (1.75 g, 8.00 mmol) was added and the reaction mixture was stirred at room temperature for 4 h. It was diluted with H_2O (60 mL) and washed with CH_2Cl_2 (2 × 30 mL). The aqueous layer was adjusted to pH ~2.5 with 5 M HCl and extracted with CH_2Cl_2 (2 × 60 mL). The combined organic layer was dried over Na_2SO_4, filtered and evaporated to dryness yielding Boc-L-Dap(Boc)-OH as a white semi-solid (1.12 g, 92%). ^{1}H NMR (500 MHz) δ 1.36 and 1.37 (2 × s, 18H), 3.22 (t, *J* = 6.1 Hz, 2H), 3.98 (dt, *J* = 8.0, 6.1 Hz, 1H), 6.75 (t, *J* = 6.3 Hz, 1H), 6.83 (d, *J* = 8.0 Hz, 1H). The CO_2H signal is not recognizable. ^{13}C NMR (125 MHz) δ 28.3,* 41.3, 53.9, 78.1, 78.4, 155.5, 155.8, 172.4. LC-MS (ESI) (MeOH; method 1), *m/z* = 305.3 ($[M+H]^+$), 322.4 ($[M+NH_4]^+$), 327.3 ($[M+Na]^+$). Compound **89** was prepared from compound **66** and Boc-L-Dap(Boc)-OH (913 mg). CC using petroleum ether/EtOAc (7:3). Yield 506 mg, 87%. Yellow oil. ^{1}H NMR (500 MHz) δ 0.77 – 0.96 (m, 2H), 1.09 – 1.20 (m, 2H), 1.22 – 1.28 (m, 1H), 1.30 (s, 9H), 1.39 (s, 9H), 1.40 – 1.48 (m, 1H), 1.53 – 1.68 (m, 5H), 1.71 (ddd, *J* = 14.9, 10.2, 5.1 Hz, 1H), 1.98 – 2.03 (m, 1H), 3.07 – 3.25 (m, 2H), 3.68 – 3.75 (m, 1H), 4.01 – 4.17 (m, 2H), 4.33 – 4.40 (m, 1H), 4.60 (app q, *J* = 6.8 Hz, 1H), 5.61 – 5.66 (m, 1H), 6.84 (t, *J* = 6.3 Hz, 1H), 7.02 (d, *J* = 8.0 Hz, 1H), 7.14 – 7.19 (m, 1H), 7.32 (t, *J* = 7.7 Hz, 2H), 7.53 (s, 1H), 7.67 – 7.72 (m, 2H). ^{13}C NMR (125 MHz) δ 25.7, 25.9, 26.2, 28.2, 28.3, 32.5, 33.1, 33.4, 38.1, 40.9, 41.6, 44.5, 47.7, 50.4, 78.2, 78.4, 114.7, 124.3, 126.3, 128.5, 134.6, 139.7, 144.8, 155.3, 155.8, 169.8. LC-MS (ESI) (MeOH; method 2), RT 11.43 min, 97% purity, *m/z* = 582.6 ($[M+H]^+$).

*(R)-2-(tert-Butoxycarbonylamino)-3-((S)-8-(cyclohexylmethyl)-2-phenyl-5,6-dihydroimidazo[1,2-a]pyrazin-7(8H)-yl)-3-oxopropane-1-sulfonic acid (**L**, R^3 = (R)-CH_2SO_3H; **90**).* To a solution of L-cysteic acid (1.69 g, 10.0 mmol) and triethylamine (2.77 mL, 2.02 g, 20.0 mmol) in anhyd DMF (34 mL) was added a solution of di-*tert*-butyl dicarbonate (2.62 g, 12.0 mmol) in anhyd DMF (17 mL). The reaction mixture was stirred at room temperature for 48 h. The solvent was evaporated *in vacuo*. The residue was redissolved in CH_2Cl_2 (35 mL) and treated with Et_2O (100 mL) under vigorous stirring. The organic solvents were decanted and discarded. The oily residue was washed with Et_2O (35 mL) and dried *in vacuo* to give Boc-L-cysteic acid as a colorless semi-solid (2.05 g, 76%). ^{1}H NMR (600 MHz) δ 1.36 (s, 9H), 2.76 (dd, *J* = 13.8, 4.7 Hz, 1H), 2.81 (dd, *J* = 13.8, 6.8 Hz, 1H),

4.07 (td, J = 6.7, 4.6 Hz, 1H), 6.86 (d, J = 6.5 Hz, 1H). The CO_2H and SO_3H signals are not recognizable. ^{13}C NMR (150 MHz) δ 28.6, 51.2, 51.4, 78.6, 155.4, 172.6. HRMS, calcd. for $C_8H_{15}NO_7S$: $[M–H]^-$ *m/z* 268.0496; found: 268.0502. Compound **90** was prepared from compound **66** (886 mg, 3.00 mmol), DIPEA (388 mg, 0.52 mL, 3.00 mmol), DCC (619 mg) and Boc-L-cysteic acid (808 mg). CC using CH_2Cl_2/MeOH (9:1). Yield 459 mg, 28%. Yellowish solid; mp > 250 °C. ^1H NMR (600 MHz) δ 0.85 – 1.03 (m, 2H), 1.04 – 1.33 (m, 4H), 1.38 (s, 9H), 1.47 – 1.72 (m, 5H), 1.91 – 2.00 (m, 1H), 2.06 – 2.14 (m, 1H), 2.60 (dd, J = 13.4, 3.7 Hz, 1H), 3.05 (dd, J = 13.3, 9.4 Hz, 1H), 3.71 – 3.82 (m, 1H), 4.06 – 4.16 (m, 1H), 4.64 – 4.76 (m, 2H), 4.76 – 4.84 (m, 1H), 5.85 – 5.99 (m, 1H), 7.36 (d, J = 7.1 Hz, 1H), 7.39 (t, J = 7.6 Hz, 1H), 7.49 (t, J = 7.6 Hz, 2H), 7.70 (d, J = 7.6 Hz, 2H), 8.00 (s, 1H). The SO_3H signal is not recognizable. ^{13}C NMR (150 MHz) δ 25.9, 26.2, 26.5, 28.7, 32.2, 33.4, 33.9, 38.2, 40.6, 45.4, 46.3, 47.4, 53.8, 78.6, 117.2, 125.5, 129.3, 129.6, 145.0, 155.5, 172.4. Two carbon signals are not recognizable. LC-MS (ESI) (MeOH; method 1), RT 11.27 min, 90% purity, *m/z* = 547.4 ($[M+H]^+$).

*tert-Butyl (S)-1-((S)-8-(Cyclohexylmethyl)-2-phenyl-5,6-dihydroimidazo[1,2-a]pyrazin-7(8H)-yl)-4-(methylthio)-1-oxobutan-2-ylcarbamate (**L**, R^3 = (S)-$CH_2CH_2SCH_3$; **91**).* From compound **66** and Boc-L-Met-OH (748 mg). CC using a gradient of petroleum ether/EtOAc (7:3) to (3:2). Yield 269 mg, 51%. Yellow semi-solid. ^1H NMR (600 MHz)♦ δ 0.77 – 0.97 (m, 2H), 1.10 – 1.31 (m, 3H), 1.39 (s, 9H), 1.46 – 1.69 (m, 6H), 1.72 (ddd, J = 14.6, 10.1, 4.6 Hz, 1H), 1.81 (q, J = 7.5 Hz, 2H), 1.96 – 2.08 (m, 4H), 2.43 – 2.48 (m, 1H), 3.71 (ddd, J = 16.2, 12.4, 4.3 Hz, 1H), 3.95 – 4.03 (m, 1H), 4.10 – 4.16 (m, 1H), 4.36 – 4.44 (m, 1H), 4.57 (app q, J = 7.5 Hz, 1H), 5.64 (dd, J = 10.1, 5.1 Hz, 1H), 7.14 – 7.19 (m, 1H), 7.24 (d, J = 8.1 Hz, 1H), 7.32 (t, J = 7.7 Hz, 2H), 7.52 (s, 1H), 7.67 – 7.71 (m, 2H). ^{13}C NMR (150 MHz) δ 14.8, 25.7, 25.9, 26.3, 28.4, 29.9, 31.0, 32.6, 33.2, 33.5, 38.1, 41.0, 44.7, 47.8, 49.7, 78.3, 114.9, 124.4, 126.4, 128.6, 134.6, 139.7, 144.9, 155.7, 171.1. LC-MS (ESI) (MeOH; method 2), RT 10.86 min, 90% purity, *m/z* = 526.8 ($[M+H]^+$).

*tert-Butyl (R)-3-(Acetamidomethylthio)-1-((S)-8-(cyclohexylmethyl)-2-phenyl-5,6-dihydroimidazo[1,2-a]pyrazin-7(8H)-yl)-1-oxopropan-2-ylcarbamate (**L**, R^3 = (S)-CH_2SCH_2NH-Ac; **92**).* From compound **66** and Boc-L-Cys(Acm)-OH (877 mg). CC using EtOAc (100%). Yield 365 mg, 64%. Yellow oil. ^1H NMR (500 MHz) δ 0.83 – 1.06 (m, 2H), 1.07 – 1.30 (m, 4H), 1.40 (s, 9H), 1.54 – 1.69 (m, 5H), 1.73 (ddd, J = 14.9, 10.0, 5.2 Hz, 1H), 1.83 (s, 3H), 1.95 – 2.04 (m, 1H), 2.72 (dd, J = 13.9, 8.4 Hz, 1H), 2.87 (dd, J = 13.9, 6.2 Hz, 1H), 3.68 (ddd, J = 15.7, 10.3, 6.0 Hz, 1H), 4.06 – 4.11 (m, 2H), 4.12 – 4.19 (m, 1H), 4.28 – 4.37 (m, 1H), 4.49 – 4.62 (m, 1H), 4.69 (app q, J = 7.8 Hz, 1H), 5.66 (dd, J = 9.6, 5.2 Hz,

1H), 7.13 – 7.20 (m, 1H), 7.28 – 7.34 (m, 3H), 7.54 (s, 1H), 7.65 – 7.73 (m, 2H), 8.46 (t, *J* = 6.6 Hz, 1H). ^{13}C NMR (125 MHz) δ 22.7, 25.7, 25.8, 26.2, 28.3, 32.2, 32.5, 33.2, 33.3, 38.3, 40.4, 40.8, 44.8, 47.7, 50.0, 78.4, 114.8, 124.3, 126.3, 128.5, 134.6, 139.7, 144.7, 155.4, 169.6, 170.4. LC-MS (ESI) (MeOH; method 1), RT 11.43 min, 97% purity, *m/z* = 570.4 ([M+H]$^+$).

*tert-Butyl (R)-3-(tert-Butyldisulfanyl)-1-((S)-8-(cyclohexylmethyl)-2-phenyl-5,6-dihydroimidazo[1,2-a]pyrazin-7(8H)-yl)-1-oxopropan-2-ylcarbamate (**L**, R^3 = (S)-CH_2S-StBu; **93**).* From compound **66** and Boc-L-Cys(StBu)-OH (928 mg). CC using petroleum ether/EtOAc (7:3). Yield 405 mg, 69%. Yellow oil. ^{1}H NMR (600 MHz) δ 0.77 – 0.98 (m, 2H), 0.98 – 1.20 (m, 3H), 1.28 (s, 9H), 1.40 (s, 9H), 1.52 – 1.80 (m, 7H), 1.93 – 2.00 (m, 1H), 2.88 (dd, *J* = 13.3, 7.7 Hz, 1H), 3.05 (dd, *J* = 13.3, 6.4 Hz, 1H), 3.72 (ddd, *J* = 15.7, 12.2, 4.1 Hz, 1H), 3.95 – 4.07 (m, 1H), 4.12 – 4.19 (m, 1H), 4.35 – 4.44 (m, 1H), 4.69 (app q, *J* = 7.5 Hz, 1H), 5.65 (dd, *J* = 10.0, 5.2 Hz, 1H), 7.14 – 7.20 (m, 1H), 7.29 – 7.35 (m, 2H), 7.46 (d, *J* = 8.3 Hz, 1H), 7.54 (s, 1H), 7.66 – 7.72 (m, 2H). ^{13}C NMR (150 MHz) δ 25.7, 25.9, 26.3, 28.3, 29.7, 32.5, 33.2, 33.4, 38.3, 40.9, 41.7, 44.7, 47.9, 48.0, 50.1, 78.6, 114.9, 124.4, 126.4, 128.6, 134.6, 139.8, 144.7, 155.3, 169.6. LC-MS (ESI) (MeOH; method 1), RT 12.41 min, 99% purity, *m/z* = 587.4 ([M+H]$^+$).

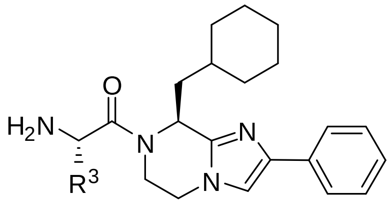

12 $R^3 = CH_2CH_3$
13 $R^3 = CH_2OH$
14 $R^3 = CH_2NH_2$
15 $R^3 = CH_2SO_3H$
16 $R^3 = CH_2CH_2SCH_3$
17 $R^3 = CH_2SCH_2NH$-Ac
18 $R^3 = CH_2S$-$SC(CH_3)_3$

*(S)-2-Amino-1-((S)-8-(cyclohexylmethyl)-2-phenyl-5,6-dihydroimidazo[1,2-a]pyrazin-7(8H)-yl)butan-1-one Dihydrochloride (**12**).* Compound **87** (240 mg, 0.50 mmol) was dissolved in anhyd CH_2Cl_2 (25 mL) and TFA (5 mL) was added. After stirring at room temperature for 2 h, volatiles were evaporated and the residue was diluted with CH_2Cl_2 (4 × 20 mL). The solvent was evaporated to remove the excess of TFA. The residue was dissolved in sat. aq. $NaHCO_3$-solution (35 mL) and extracted with EtOAc (3 × 50 mL). The combined organic layer was dried over Na_2SO_4, filtered and evaporated to dryness. The remaining oil was dissolved in anhyd EtOAc (15 mL) and a solution of 1 M HCl in EtOAc (2 mL) was added. After stirring the suspension for 1 h, the precipitate was filtered off, washed with anhyd EtOAc (20 mL), dried *in vacuo* and not further purified to give a yellowish solid (215 mg, 95%); mp > 220 °C decomp. ^{1}H NMR (500 MHz) δ 0.92 (t, *J* = 7.4 Hz, 3H), 0.95 – 1.18

(m, 3H), 1.19 – 1.34 (m, 2H), 1.36 – 1.44 (m, 1H), 1.51 – 1.61 (m, 4H), 1.69 – 1.76 (m, 1H), 1.80 – 1.88 (m, 1H), 1.92 – 1.98 (m, 2H), 2.10 – 2.16 (m, 1H), 3.85 (ddd, *J* = 15.5, 12.0, 4.0 Hz, 1H), 4.03 – 4.11 (m, 1H), 4.25 – 4.32 (m, 1H), 4.42 – 4.54 (m, 2H), 5.97 (app t, *J* = 7.3 Hz, 1H), 7.41 (t, *J* = 7.4 Hz, 1H), 7.49 (t, *J* = 7.6 Hz, 2H), 7.95 (d, *J* = 7.8 Hz, 2H), 8.12 (s, 1H), 8.45 – 8.60 (m, 3H). ^{13}C NMR (125 MHz)♦ δ 8.8, 23.8, 25.3, 25.7, 26.3, 31.9, 32.4, 33.8, 37.6, 45.5, 46.4, 50.7, 117.1, 125.5, 127.3, 129.3,* 132.6, 144.4, 168.2. LC-MS (ESI) (MeOH; method 1), RT 11.12 min, 98% purity, *m/z* = 381.2 ([M+H]$^+$). HRMS, calcd. for $C_{23}H_{32}N_4O$: [M+H]$^+$ *m/z* 381.2649; found: 381.2669.

*(S)-2-Amino-1-((S)-8-(cyclohexylmethyl)-2-phenyl-5,6-dihydroimidazo[1,2-a]pyrazin-7(8H)-yl)-3-hydroxypropan-1-one Dihydrochloride (**13**).* Compound **88** (725 mg, 1.00 mmol) was dissolved in anhyd CH_2Cl_2 (7.5 mL) under argon atm and a mixture of triisopropylsilane (2.08 mL, 1.58 g, 10.0 mmol) and TFA (7.5 mL) was added. After stirring at room temperature for 18 h, the solvent was evaporated to dryness without heating. The residue was treated with *n*-hexane (41 mL), followed by decanting the organic phase. The remaining oil was dissolved in anhyd EtOAc (20 mL) and a solution of 1 M HCl in EtOAc (4 mL) was added. After stirring the suspension for 1 h, the precipitate was filtered off, washed with anhyd EtOAc (20 mL), dried *in vacuo* and not further purified to give a greenish solid (301 mg, 66%); mp > 204 °C decomp. ^{1}H NMR (500 MHz) δ 0.89 – 1.06 (m, 2H), 1.07 – 1.31 (m, 3H), 1.34 – 1.41 (m, 1H), 1.55 – 1.66 (m, 4H), 1.83 – 1.89 (m, 1H), 1.91 – 1.99 (m, 1H), 2.08 – 2.12 (m, 1H), 3.55 (s, 1H), 3.65 – 3.75 (m, 2H), 3.80 – 3.91 (m, 1H), 4.11 – 4.22 (m, 1H), 4.24 – 4.32 (m, 1H), 4.42 – 4.51 (m, 1H), 4.53 – 4.63 (m, 1H), 5.94 (dd, *J* = 10.9, 4.0 Hz, 1H), 7.41 (t, *J* = 7.4 Hz, 1H), 7.49 (t, *J* = 7.6 Hz, 2H), 7.86 (d, *J* = 7.7 Hz, 2H), 8.05 (s, 1H), 8.34 (s, 3H). ^{13}C NMR (125 MHz)♦ δ 25.4, 25.8, 26.3, 32.0, 32.6, 33.7, 37.6, 45.6, 46.6, 52.2, 60.3, 116.9, 125.4, 127.8, 129.2, 129.4, 133.3, 144.3, 167.1. LC-MS (ESI) (MeOH; method 2), RT 8.79 min, 98% purity, *m/z* = 383.2 ([M+H]$^+$). HRMS, calcd. for $C_{22}H_{30}N_4O_2$: [M+H]$^+$ *m/z* 383.2442; found: 383.2465.

*(S)-2,3-Diamino-1-((S)-8-(cyclohexylmethyl)-2-phenyl-5,6-dihydroimidazo[1,2-a]pyrazin-7(8H)-yl)propan-1-one Trihydrochloride (**14**).* Compound **89** (436 mg, 0.75 mmol) was dissolved in anhyd CH_2Cl_2 (30 mL) and TFA (15 mL) was added. After stirring at room temperature for 2 h, volatiles were evaporated and the residue was diluted with CH_2Cl_2 (4 × 30 mL). The solvent was evaporated to remove the excess of TFA. The residue was dissolved in sat. aq. $NaHCO_3$-solution (50 mL) and extracted with EtOAc (3 × 75 mL). The combined organic layer was dried over Na_2SO_4, filtered and evaporated to dryness. The remaining oil was dissolved in anhyd EtOAc (20 mL) and a solution of 1 M HCl in EtOAc (4.5 mL) was

added. After stirring the suspension for 1 h, the precipitate was filtered off, washed with anhyd EtOAc (20 mL), dried *in vacuo* and not further purified to give a greenish solid (255 mg, 65%); mp > 230 °C decomp. ^{1}H NMR (500 MHz) δ 0.87 – 0.97 (m, 1H), 0.98 – 1.07 (m, 1H), 1.07 – 1.34 (m, 3H), 1.40 – 1.50 (m, 1H), 1.51 – 1.68 (m, 4H), 1.91 – 2.01 (m, 2H), 2.08 – 2.16 (m, 1H), 3.20 – 3.26 (m, 1H), 3.31 – 3.41 (m, 1H), 3.89 (ddd, *J* = 15.8, 11.3 Hz, 5.0 Hz, 1H), 4.20 – 4.33 (m, 2H), 4.65 – 4.75 (m, 1H), 4.92 – 5.02 (m, 1H), 5.93 (dd, *J* = 10.1, 4.8 Hz, 1H), 7.39 – 7.45 (m, 1H), 7.50 (dd, *J* = 8.5, 7.0 Hz, 2H), 7.89 – 7.96 (m, 2H), 8.16 (s, 1H), 8.88 and 8.99 (2 × s, 6H). ^{13}C NMR (125 MHz)♦♦δ 25.3, 25.7, 26.3, 31.8, 32.3, 33.8, 37.9, 45.6, 47.0, 47.9, 116.9, 125.4, 127.1, 129.3,* 132.3, 144.6, 165.6. LC-MS (ESI) (MeOH; method 1), RT 10.81 min, 98% purity, *m/z* = 382.0 ([M+H]$^+$). HRMS, calcd. for $C_{22}H_{31}N_5O$: [M+H]$^+$ *m/z* 382.2601; found: 382.2593.

*(R)-2-Amino-3-((S)-8-(cyclohexylmethyl)-2-phenyl-5,6-dihydroimidazo[1,2-a]pyrazin-7(8H)-yl)-3-oxopropane-1-sulfonic Acid Dihydrochloride (**15**).* Compound **90** (1.09 g, 2.00 mmol) was dissolved in anhyd CH_2Cl_2 (60 mL) and TFA (12 mL) was added. After stirring at room temperature for 2 h, volatiles were evaporated and the residue was diluted with CH_2Cl_2 (4 × 90 mL). The solvent was evaporated to remove the excess of TFA. The residue was dissolved in anhyd EtOAc (50 mL), treated with 1 M HCl in EtOAc (8 mL) and the precipitate was filtered off. The crude salt was dissolved in MeOH (49.4 mg/mL) and purified through semi-preparative HPLC with a gradient from 20% MeCN + 0.05% TFA / 80% H_2O + 0.05% TFA to 40% MeCN + 0.05% TFA / 60% H_2O + 0.05% TFA within 15 min (500 µL; 14.0 mL/min; 255 nm), followed by volatile evaporation and lyophilization. The obtained residue was dissolved in anhyd EtOAc (25 mL) and treated with 1 M HCl in EtOAc (4 mL). The precipitate was filtered off and dried *in vacuo* to obtain compound **15** as a white solid (51.9 mg, 5%); mp > 214 °C decomp. ^{1}H NMR (500 MHz) δ 0.90 – 1.05 (m, 2H), 1.07 – 1.31 (m, 3H), 1.35 – 1.46 (m, 1H), 1.51 – 1.78 (m, 4H), 1.83 (ddd, *J* = 14.0, 10.1, 3.7 Hz, 1H), 1.92 – 2.02 (m, 1H), 2.09 – 2.18 (m, 1H), 2.91 (dd, *J* = 14.0, 6.5 Hz, 1H), 3.03 (dd, *J* = 14.0, 5.8 Hz, 1H), 3.82 – 3.91 (m, 1H), 4.17 – 4.25 (m, 1H), 4.35 – 4.43 (m, 1H), 4.50 – 4.59 (m, 1H), 4.68 (app q, *J* = 5.6 Hz, 1H), 5.91 (dd, *J* = 11.3, 3.6 Hz, 1H), 7.43 (t, *J* = 7.6 Hz, 1H), 7.51 (t, *J* = 7.7 Hz, 2H), 7.83 (dd, *J* = 7.5, 1.8 Hz, 2H), 8.11 (s, 1H), 8.27 – 8.35 (m, 3H). ^{13}C NMR (125 MHz)♦δ 25.4, 25.8, 26.2, 31.8, 32.5, 33.8, 37.7, 44.7, 46.4, 47.6, 53.9, 117.2, 125.5, 127.1, 129.4,* 132.6, 144.2, 167.9. LC-MS (ESI) (MeOH; method 1), RT 10.49 min, 99% purity, *m/z* = 447.2 ([M+H]$^+$). HRMS, calcd. for $C_{22}H_{30}N_4O_4S$: [M+H]$^+$ *m/z* 447.2061; found: 447.2070.

*(S)-2-Amino-1-((S)-8-(cyclohexylmethyl)-2-phenyl-5,6-dihydroimidazo[1,2-a]pyrazin-7(8H)-yl)-4-(methylthio)butan-1-one dihydrochloride (**16**).* Compound **91** (263 mg, 0.50 mmol) was dissolved in anhyd CH_2Cl_2 (25 mL) and TFA (5 mL) was added. After stirring at room temperature for 2 h, volatiles were evaporated and the residue was diluted with CH_2Cl_2 (4 × 20 mL). The solvent was evaporated to remove the excess of TFA. The residue was dissolved in sat. aq. $NaHCO_3$-solution (35 mL) and extracted with EtOAc (3 × 50 mL). The combined organic layer was dried over Na_2SO_4, filtered and evaporated to dryness. The remaining oil was dissolved in anhyd EtOAc (15 mL) and a solution of 1 M HCl in EtOAc (2 mL) was added. After stirring the suspension for 1 h, the precipitate was filtered off, washed with anhyd EtOAc (15 mL) and dried *in vacuo*. The crude product was purified by CC using a gradient of $CHCl_3$ / 7 N NH_3 in MeOH (100:2.5) to (100:5). The resulting oil was dissolved in anhyd EtOAc (15 mL) and a solution of 1 M HCl in EtOAc (2 mL) was added. The precipitate was filtered off after 1 h of stirring, washed with anhyd EtOAc and dried *in vacuo* to give a brownish solid (62.4 mg, 25%); mp 128–130 °C. ^{1}H NMR (500 MHz) δ 0.88 – 1.06 (m, 2H), 1.08 – 1.19 (m, 1H), 1.20 – 1.34 (m, 2H), 1.37 – 1.43 (m, 1H), 1.52 – 1.63 (m, 4H), 1.85 – 2.09 (m, 7H), 2.08 – 2.15 (m, 1H), 2.55 – 2.67 (m, 2H), 3.87 (ddd, *J* = 15.8, 11.9, 4.1 Hz, 1H), 3.98 – 4.08 (m, 1H), 4.27 – 4.35 (m, 1H), 4.38 – 4.48 (m, 1H), 4.54 – 4.63 (m, 1H), 5.92 (dd, *J* = 10.7, 3.9 Hz, 1H), 7.39 – 7.45 (m, 1H), 7.50 (dd, *J* = 8.5, 6.9 Hz, 2H), 7.86 – 7.91 (m, 2H), 8.08 (s, 1H), 8.48 – 8.57 (m, 3H). ^{13}C NMR (125 MHz)♦ δ 14.5, 25.3, 25.7, 26.3, 27.9, 30.7, 31.9, 32.4, 33.8, 37.8, 45.4, 46.6, 49.2, 117.0, 125.4, 127.4, 129.3, 129.3, 132.8, 144.5, 167.9. LC-MS (ESI) (MeOH; method 1), RT 9.13 min, 99% purity, *m/z* = 427.3 ($[M+H]^+$). HRMS, calcd. for $C_{24}H_{34}N_4OS$: $[M+H]^+$ *m/z* 427.2526; found: 427.2556.

*N-(((R)-2-Amino-3-((S)-8-(cyclohexylmethyl)-2-phenyl-5,6-dihydroimidazo[1,2-a]pyrazin-7(8H)-yl)-3-oxopropylthio)methyl)acetamide (**17**).* Compound **92** (285 mg, 0.50 mmol) was dissolved in anhyd CH_2Cl_2 (15 mL) and TFA (3 mL) was added under argon atm. After stirring at room temperature for 2 h, volatiles were evaporated and the residue was diluted with CH_2Cl_2 (4 × 15 mL). The solvent was evaporated to remove the excess of TFA. The residue was dissolved in sat. aq. $NaHCO_3$-solution (35 mL) and extracted with EtOAc (3 × 50 mL). The combined organic layer was dried over Na_2SO_4, filtered and evaporated to dryness. The remaining oil was dissolved in anhyd EtOAc (15 mL) and a solution of 1 M HCl in EtOAc (2 mL) was added. After stirring the suspension for 1 h, the precipitate was filtered off, washed with anhyd EtOAc (15 mL) and dried *in vacuo*. The crude product was purified by CC using $CHCl_3$ / 7 N NH_3 in MeOH (100:5), yielding a colorless oil (32.9 mg, 14%). ^{1}H NMR (500 MHz) δ 0.86 – 1.05 (m, 2H), 1.07 – 1.31 (m, 3H), 1.42 – 1.50 (m, 1H), 1.58 – 1.81

(m, 6H), 1.83 (s, 3H), 1.89 – 1.97 (m, 1H), 2.60 (dd, J = 13.5, 7.1 Hz, 1H), 2.83 (dd, J = 13.5, 6.5 Hz, 1H), 3.67 (ddd, J = 15.6, 11.8, 4.4 Hz, 1H), 3.89 (app t, J = 6.8 Hz, 1H), 3.98 – 4.12 (m, 2H), 4.17 – 4.27 (m, 2H), 4.35 – 4.44 (m, 1H), 5.64 (app t, J = 7.5 Hz, 1H), 5.73 (s, 2H), 7.14 – 7.20 (m, 1H), 7.29 – 7.36 (m, 2H), 7.53 (s, 1H), 7.65 – 7.75 (m, 2H), 8.47 (t, J = 6.1 Hz, 1H). ^{13}C NMR (125 MHz) δ 22.7, 25.8, 25.9, 26.3, 32.7, 33.1, 33.4, 36.5, 38.3, 40.7, 40.8, 44.8, 47.5, 51.4, 114.8, 124.3, 126.3, 128.6, 134.6, 139.7, 144.8, 169.4, 172.9. LC-MS (ESI) (MeOH; method 2), RT 8.81 min, 93% purity, m/z = 470.1 ([M+H]$^+$). HRMS, calcd. for $C_{25}H_{35}N_5O_2S$: [M+H]$^+$ m/z 470.2584; found: 470.2578.

*(R)-2-Amino-3-(tert-butyldisulfanyl)-1-((S)-8-(cyclohexylmethyl)-2-phenyl-5,6-dihydroimidazo[1,2-a]pyrazin-7(8H)-yl)propan-1-one Dihydrochloride (**18**).* Compound **93** (293 mg, 0.50 mmol) was dissolved in anhyd CH_2Cl_2 (25 mL) and TFA (5 mL) was added under argon atm. After stirring at room temperature for 2 h, volatiles were evaporated and the residue was diluted with CH_2Cl_2 (4 × 20 mL). The solvent was evaporated to remove the excess of TFA. The residue was dissolved in sat. aq. $NaHCO_3$-solution (35 mL) and extracted with EtOAc (3 × 50 mL). The combined organic layer was dried over Na_2SO_4, filtered and evaporated to dryness. The remaining oil was dissolved in anhyd EtOAc (15 mL) and a solution of 1 M HCl in EtOAc (2 mL) was added. After stirring the suspension for 1 h, the precipitate was filtered off, washed with anhyd EtOAc (15 mL) and dried *in vacuo*. The crude product was purified by CC using $CHCl_3$ / 7 N NH_3 in MeOH (100:2.5). The resulting oil was dissolved in anhyd EtOAc (15 mL) and a solution of 1 M HCl in EtOAc (2 mL) was added. The precipitate was filtered off after 1 h of stirring, washed with anhyd EtOAc and dried *in vacuo* to give a greenish solid (75.6 mg, 27%); mp > 210 °C decomp. ^{1}H NMR (500 MHz) δ 0.88 – 1.07 (m, 2H), 1.09 – 1.19 (m, 2H), 1.22 (s, 9H), 1.24 – 1.35 (m, 1H), 1.41 – 1.67 (m, 5H), 1.92 – 2.02 (m, 2H), 2.08 – 2.15 (m, 1H), 3.17 (dd, J = 14.3, 5.8 Hz, 1H), 3.28 (dd, J = 14.3, 6.2 Hz, 1H), 3.92 (ddd, J = 15.9, 11.9, 4.4 Hz, 1H), 4.15 (ddd, J = 16.1, 9.3, 4.6 Hz, 1H), 4.32 – 4.39 (m, 1H), 4.46 – 4.55 (m, 1H), 4.70 – 4.78 (m, 1H), 5.92 – 6.01 (m, 1H), 7.41 (t, J = 7.4 Hz, 1H), 7.49 (t, J = 7.7 Hz, 2H), 7.86 – 7.96 (m, 2H), 8.15 (s, 1H), 8.78 (s, 3H). ^{13}C NMR (125 MHz)♦ δ 25.4, 25.7, 26.3, 29.5, 31.8, 32.4, 33.8, 37.8, 40.9, 45.5, 46.8, 48.3, 49.2, 117.0, 125.5, 127.3, 129.3,* 133.1, 144.2, 167.0. LC-MS (ESI) (MeOH; method 1), RT 12.00 min, 93% purity, m/z = 487.3 ([M+H]$^+$). HRMS, calcd. for $C_{26}H_{38}N_4OS_2$: [M+H]$^+$ m/z 487.2560; found: 487.2531.

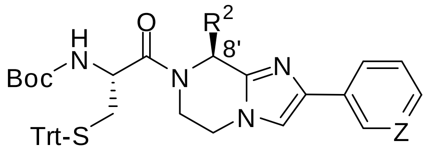

N (**94**-**101**)

*tert-Butyl (R)-1-((S)-8-Benzyl-2-phenyl-5,6-dihydroimidazo[1,2-a]pyrazin-7(8H)-yl)-1-oxo-3-(tritylthio)propan-2-ylcarbamate (**N**, R^2 = (S)-$CH_2C_6H_5$, Z = CH; **94**). Typical Procedure to Protected 8'-Substituted Derivatives **N**.* Boc-L-Cys(Trt)-OH (2.09 g, 4.50 mmol) and DCC (928 mg, 4.50 mmol) were stirred in anhyd CH_2Cl_2 (28 mL) under argon atm for 30 min. Compound **68** (434 mg, 1.50 mmol) dissolved in anhyd CH_2Cl_2 (18 mL) and DIPEA (0.26 mL, 194 mg, 1.50 mmol) were added and the reaction mixture was stirred at room temperature and argon atm for 48 h. After removing the solvent *in vacuo*, the residue was treated with a mixture of petroleum ether/EtOAc (7:3) (150 mL). The white urea was filtered off, the filtrate was evaporated to dryness and the crude oily residue was purified by preparative CC on silica gel using a gradient of petroleum ether/EtOAc (4:1) to (7:3) to obtain **94** as yellowish oil (739 mg, 67%). ^{1}H NMR (500 MHz) δ 1.17 – 1.44 (m, 9H), 2.19 – 2.27 (m, 1H), 2.39 – 2.48 (m, 1H), 3.09 – 3.29 (m, 3H), 3.55 – 4.05 (m, 3H), 4.14 – 4.59 (m, 1H), 5.41 – 5.59 (m, 1H), 6.94 – 6.99 (m, 1H), 7.09 – 7.38 (m, 23H), 7.48 – 7.54 (m, 1H), 7.70 – 7.77 (m, 2H). ^{13}C NMR (125 MHz) δ 28.2, 33.3, 38.1, 41.4, 44.1, 49.9, 51.8, 66.3, 78.5, 114.9, 124.4, 126.3, 126.5, 126.9, 128.1, 128.1, 128.6, 129.2, 129.7, 134.5, 137.1, 139.8, 143.1, 144.4, 154.9, 169.6. LC-MS (ESI) (MeOH; method 1), RT 13.03 min, 99% purity, *m/z* = 735.3 ([M+H]$^+$).

*tert-Butyl (R)-1-((S)-8-Cyclohexyl-2-phenyl-5,6-dihydroimidazo[1,2-a]pyrazin-7(8H)-yl)-1-oxo-3-(tritylthio)propan-2-ylcarbamate (**N**, R^2 = (S)-C_6H_{11}, Z = CH; **95**).* From **69** (422 mg)**.** CC using a gradient of petroleum ether/EtOAc (4:1) to (7:3). Yield 741 mg, 68%. Yellowish oil. ^{1}H NMR (500 MHz) δ 0.76 – 0.99 (m, 2H), 1.03 – 1.27 (m, 3H), 1.36 (s, 9H), 1.46 – 1.79 (m, 5H), 2.15 – 2.24 (m, 1H), 2.43 – 2.47 (m, 2H), 3.48 – 3.58 (m, 1H), 3.62 – 3.70 (m, 1H), 4.00 – 4.04 (m, 1H), 4.04 – 4.10 (m, 1H), 4.14 (app q, *J* = 7.7 Hz, 1H), 5.17 (d, *J* = 9.1 Hz, 1H), 7.15 – 7.19 (m, 1H), 7.21 – 7.26 (m, 4H), 7.26 – 7.36 (m, 14H), 7.53 (s, 1H), 7.68 – 7.71 (m, 2H). ^{13}C NMR (125 MHz) δ 25.6, 25.7, 26.0, 28.2, 28.3, 30.2, 32.9, 39.0, 40.5, 44.6, 49.6, 54.1, 66.4, 78.5, 115.0, 124.4, 126.3, 126.9, 128.2, 128.6, 129.2, 134.6, 139.5, 143.4, 144.4, 155.1, 170.0. LC-MS (ESI) (MeOH; method 1), RT 13.50 min, 96% purity, *m/z* = 727.5 ([M+H]$^+$).

*tert-Butyl (R)-1-((S)-8-Isobutyl-2-phenyl-5,6-dihydroimidazo[1,2-a]pyrazin-7(8H)-yl)-1-oxo-3-(tritylthio)propan-2-ylcarbamate (**N**, R^2 = (S)-$CH_2CH(CH_3)_2$, Z = CH; **96**).* From **70**

(383 mg). CC using a gradient of petroleum ether/EtOAc (4:1) to (7:3). Yield 641 mg, 61%. Yellow oil. ^{1}H NMR (500 MHz)♦ δ 0.84 – 0.88 (m, 3H), 0.91 (d, *J* = 5.9 Hz, 3H), 1.36 (s, 9H), 1.52 – 1.60 (m, 1H), 1.61 – 1.71 (m, 2H), 2.45 – 2.48 (m, 1H), 3.52 – 3.60 (m, 1H), 3.60 – 3.69 (m, 1H), 3.92 – 4.05 (m, 2H), 4.14 (app q, *J* = 8.0 Hz, 1H), 5.54 (dd, *J* = 9.2, 5.0 Hz, 1H), 7.14 – 7.19 (m, 1H), 7.22 – 7.27 (m, 4H), 7.27 – 7.35 (m, 14H), 7.50 (s, 1H), 7.67 – 7.71 (m, 2H). ^{13}C NMR (125 MHz) δ 22.4, 23.1, 24.2, 28.2, 33.0, 38.1, 42.4, 44.7, 48.4, 49.9, 66.3, 78.6, 114.8, 124.4, 126.4, 126.9, 128.2, 128.6, 129.2, 134.5, 139.8, 144.5, 144.5, 155.1, 169.7. LC-MS (ESI) (MeOH; method 1), RT 13.13 min, 99% purity, *m/z* = 701.5 ([M+H]$^+$).

*tert-Butyl (R)-1-((S)-8-Ethyl-2-phenyl-5,6-dihydroimidazo[1,2-a]pyrazin-7(8H)-yl)-1-oxo-3-(tritylthio)propan-2-ylcarbamate (**N**, R^2 = (S)-CH_2CH_3, Z = CH; **97**).* From **71** (341 mg). CC using a gradient of petroleum ether/EtOAc (4:1) to (7:3). Yield 727 mg, 72%. Yellow solid; mp 114–116 °C. ^{1}H NMR (500 MHz) δ 0.79 – 0.90 (m, 3H), 1.36 (s, 9H), 1.68 – 1.80 (m, 1H), 1.82 – 1.92 (m, 1H), 2.44 – 2.48 (m, 2H), 3.48 – 3.57 (m, 1H), 3.61 – 3.69 (m, 1H), 3.94 – 4.05 (m, 2H), 4.20 (app q, *J* = 8.0 Hz), 5.36 (dd, *J* = 9.0, 5.4 Hz, 1H), 7.14 – 7.20 (m, 1H), 7.21 – 7.37 (m, 18H), 7.52 (s, 1H), 7.68 – 7.72 (m, 2H). ^{13}C NMR (125 MHz) δ 10.3, 26.5, 28.2, 33.2, 38.6, 44.6, 50.0, 51.3, 66.3, 78.6, 114.8, 124.4, 126.4, 126.9, 128.2, 128.6, 129.2, 134.5, 139.8, 144.5, 144.6, 155.1, 169.8. LC-MS (ESI) (MeOH; method 1), RT 12.75 min, 98% purity, *m/z* = 673.1 ([M+H]$^+$).

*(R)-tert-Butyl 1-(8,8-Dimethyl-2-phenyl-5,6-dihydroimidazo[1,2-a]pyrazin-7(8H)-yl)-1-oxo-3-(tritylthio)propan-2-ylcarbamate (**N**, R^2 = $(CH_3)_2$, Z = CH; **98**).* From **72** (341 mg). CC using a gradient of petroleum ether/EtOAc (4:1) to (7:3). Yield 404 mg, 40%. Colorless oil. ^{1}H NMR (500 MHz) δ 1.30 – 1.39 (m, 9H), 1.71 and 1.72 (2 × s, 6H), 2.37 – 2.47 (m, 2H), 3.38 – 3.46 (m, 1H), 3.51 – 3.59 (m, 1H), 3.84 – 3.95 (m, 2H), 4.19 (app q, *J* = 7.4 Hz, 1H), 7.15 – 7.19 (m, 1H), 7.21 – 7.27 (m, 4H), 7.29 – 7.35 (m, 14H), 7.50 (s, 1H), 7.68 – 7.72 (m, 2H). ^{13}C NMR (125 MHz) δ 26.0, 26.7, 28.2, 33.8, 42.1, 44.0, 52.0, 59.2, 66.3, 78.6, 114.9, 124.4, 126.4, 126.9, 128.2, 128.6, 129.3, 134.6, 139.6, 144.5, 150.1, 155.0, 170.9. LC-MS (ESI) (MeOH; method 1), RT 13.31 min, 99% purity, *m/z* = 673.2 ([M+H]$^+$).

*tert-Butyl (R)-1-((R)-8-(tert-Butoxymethyl)-2-phenyl-5,6-dihydroimidazo[1,2-a]pyrazin-7(8H)-yl)-1-oxo-3-(tritylthio)propan-2-ylcarbamate (**N**, R^2 = (R)-CH_2OtBu, Z = CH; **99**).* From **73** (428 mg). CC on silica gel using petroleum ether/EtOAc (7:3). Yield 855 mg, 78%. Yellow solid; mp 107–109 °C. ^{1}H NMR (500 MHz)♦ δ 1.07 (s, 9H), 1.36 (s, 9H), 2.28 – 2.39 (m, 1H), 3.58 – 3.78 (m, 3H), 3.78 – 3.87 (m, 1H), 4.02 – 4.10 (m, 1H), 4.55 – 4.63 (m, 1H), 4.67 – 4.76 (m, 1H), 5.26 – 5.38 (m, 1H), 7.14 – 7.42 (m, 19H), 7.54 (d, *J* = 2.9 Hz, 1H), 7.67 – 7.73 (m, 2H). ^{13}C NMR (125 MHz) δ 27.3, 28.2, 33.5, 35.3, 43.8, 49.9, 53.6, 63.6, 66.4,

73.4, 78.7, 115.3, 124.3, 126.4, 126.8, 128.1, 128.6, 129.2, 134.5, 140.1, 142.3, 144.7, 154.8, 169.6. LC-MS (ESI) (MeOH; method 1), RT 13.07 min, 94% purity, *m/z* = 730.7 ($[M+H]^+$).

*tert-Butyl 2-((S)-7-((R)-2-(tert-Butoxycarbonylamino)-3-(tritylthio)propanoyl)-2-phenyl-5,6,7,8-tetrahydroimidazo[1,2-a]pyrazin-8-yl)acetate (**N**, R^2 = (S)-CH_2CO_2tBu, Z = CH; **100**).* From **74** (470 mg). CC using petroleum ether/EtOAc (7:3). Yield 911 mg, 80%. Yellow solid; mp 104–106 °C. ^{1}H NMR (500 MHz) δ 1.31 – 1.41 (m, 18H), 2.39 – 2.48 (m, 2H), 2.64 – 2.84 (m, 2H), 3.53 – 3.81 (m, 2H), 3.87 – 3.97 (m, 1H), 4.02 – 4.10 (m, 1H), 4.21 – 4.30 (m, 1H), 5.68 (t, 1H, *J* = 6.2 Hz), 7.22 – 7.27 (m, 3H), 7.28 – 7.37 (m, 16H), 7.58 (s, 1H), 7.64 – 7.73 (m, 2H). ^{13}C NMR (125 MHz)♦♦ δ 27.8, 28.2, 33.5, 44.4, 47.9, 50.0, 66.3, 78.6, 80.3, 115.3, 124.4, 126.8, 126.9, 128.1, 128.2, 128.6, 129.2, 134.5, 139.7, 142.9, 144.5, 154.9, 168.7, 169.4. LC-MS (ESI) (MeOH; method 2), RT 12.06 min, 96% purity, *m/z* = 759.4 ($[M+H]^+$).

*tert-Butyl (R)-1-((S)-8-(Cyclohexylmethyl)-2-(pyridin-3-yl)-5,6-dihydroimidazo[1,2-a]pyrazin-7(8H)-yl)-1-oxo-3-(tritylthio)propan-2-ylcarbamate (**N**, R^2 = (S)-$CH_2C_6H_{11}$, Z = N; **101**).* From **75** (445 mg). CC using a gradient of petroleum ether/EtOAc (3:7) to EtOAc (100%). Yield 579 mg, 52%. Yellow oil. ^{1}H NMR (500 MHz)♦ δ 0.76 – 1.05 (m, 2H), 1.06 – 1.33 (m, 3H), 1.37 (s, 9H), 1.51 – 1.70 (m, 7H), 1.88 – 1.95 (m, 1H), 2.45 – 2.48 (m, 1H), 3.52 – 3.67 (m, 2H), 3.94 – 4.03 (m, 2H), 4.17 (app q, *J* = 7.7 Hz, 1H), 5.58 (dd, *J* = 9.7, 5.5 Hz, 1H), 7.20 – 7.38 (m, 17H), 7.65 (s, 1H), 8.02 (dt, *J* = 8.0, 1.9 Hz, 1H), 8.33 – 8.40 (m, 1H), 8.86 – 8.93 (m, 1H). ^{13}C NMR (125 MHz) δ 25.6, 25.8, 26.2, 28.3, 32.5, 33.1, 33.1, 33.3, 38.0, 40.7, 44.8, 47.8, 50.0, 66.4, 78.5, 115.8, 123.8, 127.0, 128.2, 129.2, 130.2, 131.4, 136.8, 144.4, 145.2, 145.9, 147.4, 155.0, 169.7. LC-MS (ESI) (MeOH; method 1), RT 13.15 min, 99% purity, *m/z* = 742.6 ($[M+H]^+$).

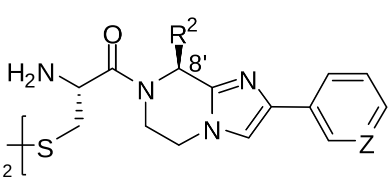

19 $R^2 = CH_2C_6H_5$, Z = CH
20 $R^2 = C_6H_{11}$, Z = CH
21 $R^2 = CH_2CH(CH_3)_2$, Z = CH
22 $R^2 = CH_2CH_3$, Z = CH
23 $R^2 = (CH_3)_2$, Z = CH
24 $R^2 = CH_2OH$, Z = CH
25 $R^2 = CH_2CO_2H$, Z = CH
26 $R^2 = CH_2C_6H_{11}$, Z = N

*(R)-2-Amino-3-(((S)-2-amino-3-((R)-8-benzyl-2-phenyl-5,6-dihydroimidazo[1,2-a]pyrazin-7(8H)-yl)-3-oxopropyl)disulfanyl)-1-((S)-8-benzyl-2-phenyl-5,6-dihydroimidazo[1,2-a]pyrazin-7(8H)-yl)propan-1-one Tetrahydrochloride (**19**). Typical Procedure to Deprotected 8'-Substituted Derivatives **19-26**.* Compound **94** (735 mg, 1.00 mmol) was dissolved in anhyd CH_2Cl_2 (8 mL) under argon atm and a mixture of triisopropylsilane (2.08 mL, 1.58 g, 10.0

mmol) and TFA (8 mL) was added. After stirring at room temperature for 18 h, the solvent was evaporated to dryness without heating. The residue was treated with *n*-hexane (40 mL), followed by decanting the organic phase. The remaining oil was dissolved in anhyd EtOAc (35 mL) and treated with 1 M HCl in EtOAc (4 mL). The precipitate was filtered off, washed with anhyd EtOAc (2 × 30 mL) and dried *in vacuo*. The crude salt was dissolved in MeOH (30 mL) containing H_2O (0.6 mL). A solution of iodine (254 mg, 1.00 mmol) in MeOH (30 mL) was added dropwise under vigorous stirring and the reaction mixture was stirred at room temperature for 2 h. After evaporating the solvent *in vacuo*, the crude residue was purified by means of preparative CC using a gradient of $CHCl_3$ / 7 N NH_3 in MeOH (100:2.5) to (100:5). The resulting oil was redissolved in anhyd EtOAc (35 mL) and treated with 1 M HCl in EtOAc (4 mL). The precipitate was filtered off, washed with anhyd EtOAc (2 × 30 mL) and dried *in vacuo*. The crude salt was dissolved in MeOH (118 mg/mL) and purified through semi-preparative HPLC with a gradient from 30% MeCN + 0.05% TFA / 70% H_2O + 0.05% TFA to 45% MeCN + 0.05% TFA / 55% H_2O + 0.05% TFA within 15 min (100 µL; 14.0 mL/min; 255 nm), followed by volatile evaporation and lyophilization. The residue was redissolved in anhyd EtOAc (35 mL) and treated with 1 M HCl in EtOAc (4 mL). The precipitate was again filtered off, washed with anhyd EtOAc (2 × 30 mL) and dried *in vacuo* to obtain compound **19** as a white solid (37.2 mg, 8%); mp > 224 °C decomp. ^{1}H NMR (600 MHz) δ 3.25 (s, 2H), 3.94 – 4.18 (m, 4H), 4.18 – 4.31 (m, 2H), 4.59 – 4.75 (m, 2H), 5.70 – 5.92 (m, 2H), 7.16 – 7.29 (m, 6H), 7.30 – 7.45 (m, 6H), 7.46 – 7.55 (m, 4H), 7.88 – 7.96 (m, 4H), 7.96 – 8.11 (m, 2H), 8.55 – 8.83 (m, 6H). Four proton signals are obscured by the water peak. ^{13}C NMR (150 MHz) of two rotamers δ 35.9, 36.1, 37.9, 38.0, 38.5, 38.7, 44.2, 44.8, 51.5, 51.5, 54.7, 54.8, 116.7, 116.9, 125.3, 125.3, 125.4, 127.3, 127.7, 127.8, 128.7, 128.9, 129.2, 129.3, 129.4, 129.4, 129.9, 130.0, 130.0, 130.1, 133.6, 134.9, 136.1, 136.2, 142.6, 142.9, 166.7, 167.9. LC-MS (ESI) (MeOH; method 1), RT 11.80 min, 99% purity, *m/z* = 783.2 ($[M+H]^+$). HRMS, calcd. for $C_{44}H_{46}N_8O_2S_2$: $[M+H]^+$ *m/z* 783.3258; found: 783.3261.

*(S,2R,2'R)-3,3'-Disulfanediylbis(2-amino-1-((S)-8-cyclohexyl-2-phenyl-5,6-dihydroimidazo[1,2-a]pyrazin-7(8H)-yl)propan-1-one) Tetrahydrochloride (**20**).* From **95** (727 mg). CC using a gradient of $CHCl_3$ / 7 N NH_3 in MeOH (100:2.5) to (100:5). After precipitation, the crude salt in MeOH (98.0 mg/mL) was purified through semi-preparative HPLC with a gradient from 10% MeCN + 0.05% TFA / 90% H_2O + 0.05% TFA to 30% MeCN + 0.05% TFA / 70% H_2O + 0.05% TFA within 15 min (150 µL; 14.0 mL/min; 255 nm). Yield 32.0 mg, 7%. Yellow solid; mp > 214 °C decomp. ^{1}H NMR (500 MHz) δ 1.03 – 1.25 (m, 8H), 1.35 – 1.50 (m, 2H), 1.56 – 1.64 (m, 2H), 1.64 – 1.78 (m, 8H), 2.09 – 2.21 (m,

2H), 3.20 – 3.36 (m, 4H), 3.87 – 3.97 (m, 2H), 4.20 – 4.32 (m, 2H), 4.33 – 4.40 (m, 2H), 4.43 – 4.56 (m, 2H), 4.80 – 4.92 (m, 2H), 5.61 (d, *J* = 7.9 Hz, 2H), 7.40 (t, *J* = 7.5 Hz, 2H), 7.48 (t, *J* = 7.6 Hz, 4H), 7.81 – 7.97 (m, 4H), 8.13 (s, 2H), 8.79 (s, 6H). ^{13}C NMR (125 MHz)♦ δ 25.6, 25.7, 25.9, 29.0, 29.7, 38.7, 41.3, 45.1, 49.0, 53.1, 117.3, 125.6, 127.7, 129.1, 129.2, 133.4, 142.5, 167.0. LC-MS (ESI) (MeOH; method 1), RT 12.41 min, 95% purity, *m/z* = 767.2 ([M+H]$^+$). HRMS, calcd. for $C_{42}H_{54}N_8O_2S_2$: [M+H]$^+$ *m/z* 767.3884; found: 767.3900.

*(S,2R,2'R)-3,3'-Disulfanediylbis(2-amino-1-((S)-8-isobutyl-2-phenyl-5,6-dihydroimidazo[1,2-a]pyrazin-7(8H)-yl)propan-1-one) Tetrahydrochloride (**21**).* From **96** (701 mg). CC using a gradient of $CHCl_3$ / 7 N NH_3 in MeOH (100:2.5) to (100:5). After precipitation, the crude salt in MeOH (83.0 mg/mL) was purified through semi-preparative HPLC with a gradient from 20% MeCN + 0.05% TFA / 80% H_2O + 0.05% TFA to 50% MeCN + 0.05% TFA / 50% H_2O + 0.05% TFA within 16.5 min (100 µL; 14.0 mL/min; 255 nm). Yield 51.6 mg, 12%. White solid; mp > 206 °C decomp. ^{1}H NMR (500 MHz) δ 0.89 (d, *J* = 6.5 Hz, 6H), 1.05 (d, *J* = 6.3 Hz, 6H), 1.70 – 1.79 (m, 2H), 1.84 (ddd, *J* = 13.7, 10.0, 4.0 Hz, 2H), 1.96 – 2.06 (m, 2H), 3.25 – 3.35 (m, 4H), 3.91 (ddd, *J* = 15.6, 11.5, 4.5 Hz, 2H), 4.18 – 4.27 (m, 2H), 4.28 – 4.36 (m, 2H), 4.42 – 4.52 (m, 2H), 4.80 – 4.91 (m, 2H), 5.87 (dd, *J* = 10.6, 4.0 Hz, 2H), 7.39 (t, *J* = 7.4 Hz, 2H), 7.48 (t, *J* = 7.6 Hz, 4H), 7.85 – 7.91 (m, 4H), 8.06 (s, 2H), 8.78 (s, 6H). ^{13}C NMR (125 MHz) δ 21.9, 23.6, 23.7, 38.1, 38.5, 41.6, 45.4, 47.6, 48.9, 116.8, 125.4, 128.1, 129.0, 129.3, 133.8, 143.9, 166.8. LC-MS (ESI) (MeOH; method 1), RT 11.96 min, 97% purity, *m/z* = 715.7 ([M+H]$^+$). HRMS, calcd. for $C_{38}H_{50}N_8O_2S_2$: [M+H]$^+$ *m/z* 715.3571; found: 715.3583.

*(S,2R,2'R)-3,3'-Disulfanediylbis(2-amino-1-((S)-8-ethyl-2-phenyl-5,6-dihydroimidazo[1,2-a]pyrazin-7(8H)-yl)propan-1-one) Tetrahydrochloride (**22**).* From **97** (673 mg). CC using $CHCl_3$ / 7 N NH_3 in MeOH (100:5). After precipitation, the crude salt in MeCN (143 mg/mL) was purified through semi-preparative HPLC with a gradient from 20% MeCN + 0.05% TFA / 80% H_2O + 0.05% TFA to 30% MeCN + 0.05% TFA / 70% H_2O + 0.05% TFA within 15 min (100 µL; 14.0 mL/min; 255 nm). Yield 76.4 mg, 19%. Yellowish solid; mp > 126 °C decomp. ^{1}H NMR (500 MHz) δ 0.97 (t, *J* = 7.4 Hz, 6H), 1.99 – 2.12 (m, 2H), 2.15 – 2.31 (m, 2H), 3.24 – 3.35 (m, 4H), 3.88 (ddd, *J* = 15.9, 11.6, 4.5 Hz, 2H), 4.19 – 4.30 (m, 2H), 4.30 – 4.40 (m, 2H), 4.43 – 4.54 (m, 2H), 4.85 (app t, *J* = 5.9 Hz, 2H), 5.75 (dd, *J* = 10.1, 4.4 Hz, 2H), 7.41 (t, *J* = 7.5 Hz, 2H), 7.44 – 7.53 (m, 4H), 7.83 – 7.92 (m, 4H), 8.11 (s, 2H), 8.77 (s, 6H). ^{13}C NMR (125 MHz) δ 10.5, 26.3, 38.5, 38.8, 45.5, 49.0, 50.2, 117.0, 125.4, 127.7, 129.4,* 133.5, 143.6, 167.0. LC-MS (ESI) (MeOH; method 1), RT 11.07 min, 100% purity,

m/z = 659.5 ([M+H]$^+$). HRMS, calcd. for $C_{34}H_{42}N_8O_2S_2$: [M+H]$^+$ *m/z* 659.2945; found: 659.2954.

*(2R,2'R)-3,3'-Disulfanediylbis(2-amino-1-(8,8-dimethyl-2-phenyl-5,6-dihydroimidazo[1,2-a]pyrazin-7(8H)-yl)propan-1-one) Tetrahydrochloride (**23**).* From **98** (673 mg). CC using a gradient of $CHCl_3$ / 7 N NH_3 in MeOH (100:2.5) to (100:5). After precipitation, the crude salt in MeOH (96.3 mg/mL) was purified through semi-preparative HPLC with an isocratic elution using 20% MeCN + 0.05% TFA / 80% H_2O + 0.05% TFA for 15 min (100 µL; 15.0 mL/min; 255 nm). Yield 52.3 mg, 13%. White solid; mp > 200 °C decomp. ^{1}H NMR (500 MHz) δ 2.05 and 2.05 (2 × s, 12H), 3.33 – 3.56 (m, 4H), 3.94 – 4.03 (m, 2H), 4.07 – 4.16 (m, 2H), 4.32 – 4.47 (m, 4H), 4.82 – 4.92 (m, 2H), 7.39 – 7.43 (m, 2H), 7.48 (t, *J* = 7.6 Hz, 4H), 7.99 (dd, *J* = 7.6, 2.0 Hz, 4H), 8.13 (s, 2H), 8.74 (s, 6H). ^{13}C NMR (125 MHz) δ 24.9, 25.2, 38.6, 42.0, 45.4, 50.5, 59.3, 117.1, 125.9, 127.7, 129.1,* 134.0, 148.5, 167.8. LC-MS (ESI) (MeOH; method 1), RT 11.17 min, 99% purity, *m/z* = 659.6 ([M+H]$^+$). HRMS, calcd. for $C_{34}H_{42}N_8O_2S_2$: [M+H]$^+$ *m/z* 659.2945; found: 659.2961.

*(R,2R,2'R)-3,3'-Disulfanediylbis(2-amino-1-((R)-8-(hydroxymethyl)-2-phenyl-5,6-dihydroimidazo[1,2-a]pyrazin-7(8H)-yl)propan-1-one) Tetrahydrochloride (**24**).* From **99** (731 mg), anhyd CH_2Cl_2 (12 mL) and TFA (12 mL). CC using $CHCl_3$ / 7 N NH_3 in MeOH (5:1). After precipitation, the crude salt in MeOH (162 mg/mL) was purified through semi-preparative HPLC with a gradient from 30% MeCN + 0.05% TFA / 70% H_2O + 0.05% TFA to 45% MeCN + 0.05% TFA / 55% H_2O + 0.05% TFA within 15 min (100 µL; 14.0 mL/min; 255 nm). Yield 16.2 mg, 4%. Yellow solid; mp > 236 °C decomp. ^{1}H NMR (500 MHz) δ 3.13 – 3.36 (m, 2H), 3.93 – 4.23 (m, 4H), 4.24 – 4.35 (m, 2H), 4.39 – 4.53 (m, 2H), 4.67 – 4.88 (m, 2H), 5.55 – 5.72 (m, 2H), 7.34 – 7.45 (m, 2H), 7.45 – 7.54 (m, 4H), 7.84 – 7.94 (m, 4H), 8.02 – 8.18 (m, 2H), 8.61 – 8.85 (m, 6H). Four proton signals are obscured by the water peak. ^{13}C NMR (125 MHz) of two rotamers δ 36.0, 36.3, 37.7, 38.1, 44.3, 45.1, 48.9, 49.2, 52.0, 62.7, 117.0, 117.1, 125.2, 125.5, 127.8, 129.3, 133.7, 142.0, 142.1, 166.8, 168.1. LC-MS (ESI) (MeCN; method 1), RT 7.06 min, 97% purity, *m/z* = 663.1 ([M+H]$^+$). HRMS, calcd. for $C_{32}H_{38}N_8O_4S_2$: [M+H]$^+$ *m/z* 663.2530; found: 663.2517.

*(R,2R,2'R)-3,3'-Disulfanediylbis(2-amino-1-((R)-8-(carboxymethyl)-2-phenyl-5,6-dihydroimidazo[1,2-a]pyrazin-7(8H)-yl)propan-1-one) Tetrahydrochloride (R^2 = (S)-CH_2CO_2H, Z = CH; **25**).* From **100** (759 mg), anhyd CH_2Cl_2 (12 mL) and TFA (12 mL). No CC was applied after the iodine-promoted oxidation. After precipitation, the crude salt in MeOH (57.1 mg/mL) was purified through semi-preparative HPLC with a gradient from 20% MeCN + 0.05% TFA / 80% H_2O + 0.05% TFA to 30% MeCN + 0.05% TFA / 70% H_2O +

0.05% TFA within 15 min (200 µL; 14.0 mL/min; 255 nm). Yield 73.5 mg, 17%. Yellow solid; mp > 250 °C decomp. ^{1}H NMR (600 MHz) δ 3.07 (dd, *J* = 16.7, 4.1 Hz, 2H), 3.24 – 3.30 (m, 4H), 3.38 (dd, *J* = 14.5, 6.6 Hz, 2H), 3.91 – 4.06 (m, 2H), 4.35 (dd, *J* = 12.4, 3.8 Hz, 2H), 4.40 – 4.50 (m, 4H), 4.94 (app t, *J* = 6.3 Hz, 2H), 6.06 (dd, *J* = 7.3, 4.1 Hz, 2H), 7.39 (t, *J* = 7.5 Hz, 2H), 7.48 (t, *J* = 7.7 Hz, 4H), 7.86 (d, *J* = 7.7 Hz, 4H), 8.09 (s, 2H), 8.79 (s, 6H). The CO_2H signal is not recognizable. ^{13}C NMR (150 MHz)♦ δ 37.2, 38.1, 45.4, 46.2, 48.9, 117.0, 125.4, 128.6, 129.0, 129.3, 134.5, 142.5, 167.5, 171.0. LC-MS (ESI) (MeOH; method 1), RT 8.97 min, 98% purity, *m/z* = 719.1 ([M+H]$^+$). HRMS, calcd. for $C_{34}H_{38}N_8O_6S_2$: [M+H]$^+$ *m/z* 719.2428; found: 719.2433.

*(R)-2-Amino-3-(((S)-2-amino-3-((R)-8-(cyclohexylmethyl)-2-(pyridin-3-yl)-5,6-dihydroimidazo[1,2-a]pyrazin-7(8H)-yl)-3-oxopropyl)disulfanyl)-1-((S)-8-(cyclohexylmethyl)-2-(pyridin-3-yl)-5,6-dihydroimidazo[1,2-a]pyrazin-7(8H)-yl)propan-1-one Hexahydrochloride (**26**).* From **101** (742 mg). For precipitation, 1 M HCl in EtOAc (6 mL) was used. CC was performed with $CHCl_3$ / 7 N NH_3 in MeOH (100:5). HPLC purification was not carried out. Yield 193 mg, 38%. Greenish solid; mp > 220 °C decomp. ^{1}H NMR (600 MHz) δ 0.86 – 0.98 (m, 4H), 1.07 – 1.35 (m, 6H), 1.48 – 1.55 (m, 2H), 1.55 – 1.73 (m, 8H), 1.75 – 1.87 (m, 4H), 1.93 – 1.99 (m, 2H), 3.23 (dd, *J* = 14.6, 5.6 Hz, 2H), 3.35 (dd, *J* = 14.6, 5.5 Hz, 2H), 3.83 – 3.90 (m, 2H), 4.16 – 4.24 (m, 2H), 4.28 – 4.35 (m, 2H), 4.40 – 4.48 (m, 2H), 4.78 – 4.86 (m, 2H), 5.71 (dd, *J* = 9.1, 5.6 Hz, 2H), 7.97 (dd, *J* = 8.3, 5.4 Hz, 2H), 8.25 (s, 2H), 8.73 (dd, *J* = 5.5, 1.4 Hz, 2H), 8.79 – 8.90 (m, 8H), 9.24 (d, *J* = 2.1 Hz, 2H). ^{13}C NMR (150 MHz) δ 25.5, 25.7, 26.3, 32.4, 32.7, 33.3, 38.3, 38.5, 40.4, 45.3, 47.9, 48.8, 119.3, 126.9, 130.4, 131.9, 138.8, 139.4, 141.8, 145.4, 166.5. LC-MS (ESI) (MeOH; method 1), RT 11.81 min, 97% purity, *m/z* = 797.6 ([M+H]$^+$). HRMS, calcd. for $C_{42}H_{56}N_{10}O_2S_2$: [M+H]$^+$ *m/z* 797.4102; found: 797.4104.

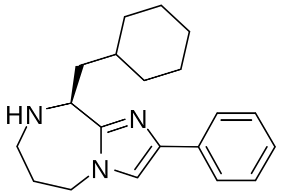

S (**102**)

*(S)-9-(Cyclohexylmethyl)-2-phenyl-6,7,8,9-tetrahydro-5H-imidazo[1,2-a][1,4]diazepine (**102** ≡ **S**).* The corresponding lactam **Q**[6] (1.62 g, 5.00 mmol) was dissolved in anhyd THF (80 mL) under argon atm. 1 M Borane-THF complex solution (30 mL) was added dropwise over a period of 10 min, followed by heating the mixture to reflux for 48 h. After evaporating

the solvent *in vacuo*, the residue was redissolved in anhyd MeOH (60 mL) and added to a Schlenk flask containing Pd/C (10% Pd) under argon atm. The reaction mixture was stirred at room temperature over night. The catalyst was filtered off through celite and rinsed with MeOH (2 × 50 mL). The solvent was evaporated to dryness and the crude residue was purified by CC on silica gel using EtOAc/MeOH (9:1). Compound **102** was isolated as yellow oil (789 mg, 51%). ^{1}H NMR (600 MHz) δ 0.83 – 0.92 (m, 1H), 0.96 – 1.04 (m, 1H), 1.10 – 1.26 (m, 3H), 1.52 – 1.79 (m, 8H), 1.79 – 1.85 (m, 1H), 1.93 (tt, *J* = 9.6, 4.7 Hz, 1H), 2.86 (ddd, *J* = 13.4, 10.3, 2.6 Hz, 1H), 3.22 (dt, *J* = 13.9, 4.0 Hz, 1H), 3.76 (dd, *J* = 8.8, 5.0 Hz, 1H), 4.02 – 4.09 (m, 1H), 4.11 – 4.18 (m, 1H), 7.11 – 7.15 (m, 1H), 7.30 (t, *J* = 7.7 Hz, 2H), 7.46 (s, 1H), 7.67 (dd, *J* = 8.2, 1.4 Hz, 2H). The NH signal is not recognizable. ^{13}C NMR (150 MHz)♦ δ 26.0, 26.2, 26.4, 30.9, 32.5, 33.8, 34.0, 46.9, 49.3, 53.0, 117.9, 124.1, 125.8, 128.5, 135.2, 136.8, 153.5. LC-MS (ESI) (MeOH; method 1), RT 11.73 min, 99% purity, *m/z* = 309.9 ([M+H]$^{+}$).

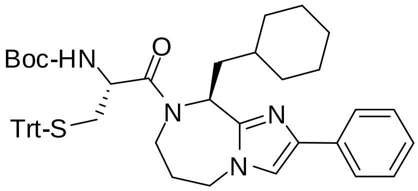

T (**103**)

*tert-Butyl (R)-1-((S)-9-(Cyclohexylmethyl)-2-phenyl-6,7-dihydro-5H-imidazo[1,2-a][1,4]diazepin-8(9H)-yl)-1-oxo-3-(tritylthio)propan-2-ylcarbamate (**103** ≡ **T**).* Boc-L-Cys(Trt)-OH (1.39 g, 3.00 mmol) and DCC (619 mg, 3.00 mmol) were stirred in anhyd CH_2Cl_2 (19 mL) under argon atm for 30 min. Compound **102** (309 mg, 1.00 mmol) dissolved in anhyd CH_2Cl_2 (12 mL) and DIPEA (0.17 mL, 129 mg, 1.00 mmol) were added and the reaction mixture was stirred at room temperature and argon atm for 48 h. After removing the solvent *in vacuo*, the residue was treated with a mixture of petroleum ether/EtOAc (7:3) (100 mL). The white urea was filtered off, the filtrate was evaporated to dryness and the crude oily residue was purified by preparative CC on silica gel using a gradient of petroleum ether/EtOAc (4:1) to (7:3) to obtain **103** as yellowish oil (596 mg, 79%). ^{1}H NMR (500 MHz) δ 0.77 – 0.88 (m, 1H), 0.89 – 0.97 (m, 1H), 1.04 – 1.14 (m, 3H), 1.35 (s, 9H), 1.39 – 1.83 (m, 8H), 1.85 – 1.93 (m, 1H), 1.94 – 2.03 (m, 1H), 2.32 (dd, *J* = 12.3, 6.3 Hz, 1H), 2.41 (dd, *J* = 12.3, 8.2 Hz, 1H), 3.89 – 4.03 (m, 3H), 4.04 – 4.13 (m, 1H), 4.18 (app q, *J* = 7.8 Hz, 1H), 5.72 – 5.94 (m, 1H), 7.12 – 7.16 (m, 1H), 7.19 – 7.24 (m, 3H), 7.24 – 7.36 (m, 15H), 7.45 (s, 1H), 7.63 – 7.67 (m, 2H). ^{13}C NMR (125 MHz) δ 25.6, 25.9, 26.2, 28.3, 29.5, 31.7, 33.3,*

33.4, 37.5, 40.9, 45.8, 50.1, 59.9, 66.4, 78.5, 118.4, 124.2, 126.1, 126.9, 128.2, 128.5, 129.2, 134.6, 137.6, 144.4, 148.3, 155.0, 170.6. LC-MS (ESI) (MeOH; method 1), RT 13.70 min, 100% purity, *m/z* = 755.4 ([M+H]$^+$).

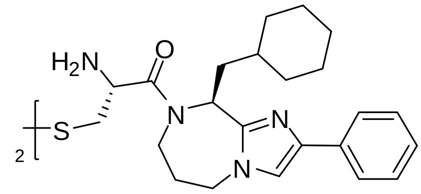

27

*(S,2R,2'R)-3,3'-Disulfanediylbis(2-amino-1-((S)-9-(cyclohexylmethyl)-2-phenyl-6,7-dihydro-5H-imidazo[1,2-a][1,4]diazepin-8(9H)-yl)propan-1-one) Tetrahydrochloride (**27**).* Compound **103** (566 mg, 0.75 mmol) was dissolved in anhyd CH_2Cl_2 (6 mL) under argon atm and a mixture of triisopropylsilane (1.56 mL, 1.19 g, 7.50 mmol) and TFA (6 mL) was added. After stirring at room temperature for 18 h, the solvent was evaporated to dryness without heating. The residue was treated with *n*-hexane (30 mL), followed by decanting the organic phase. The remaining oil was dissolved in anhyd EtOAc (20 mL) and treated with 1 M HCl in EtOAc (3 mL). The precipitate was filtered off, washed with anhyd EtOAc (2 × 25 mL) and dried *in vacuo*. The crude salt was dissolved in MeOH (25 mL) containing H_2O (0.5 mL). A solution of iodine (190 mg, 0.75 mmol) in MeOH (25 mL) was added dropwise under vigorous stirring and the reaction mixture was stirred at room temperature for 2 h. After evaporating the solvent in vacuo, the crude residue was purified by means of preparative CC using $CHCl_3$ / 7 N NH_3 in MeOH (100:2.5) to (100:5). The resulting oil was redissolved in anhyd EtOAc (20 mL) and treated with 1 M HCl in EtOAc (3 mL). The precipitate was filtered off, washed with anhyd EtOAc (2 × 25 mL) and dried *in vacuo*. The crude salt was dissolved in MeCN (141 mg/mL) and purified through semi-preparative HPLC with a gradient from 20% MeCN + 0.05% TFA / 80% H_2O + 0.05% TFA to 50% MeCN + 0.05% TFA / 50% H_2O + 0.05% TFA within 15 min (100 µL; 14.0 mL/min; 255 nm), followed by volatile evaporation and lyophilization. The residue was redissolved in anhyd EtOAc (20 mL) and treated with 1 M HCl in EtOAc (3 mL). The precipitate was again filtered off, washed with anhyd EtOAc (2 × 25 mL) and dried *in vacuo* to obtain compound **27** as a yellow solid (43.6 mg, 12%); mp > 194 °C decomp. ^{1}H NMR (500 MHz) δ 0.86 – 1.03 (m, 4H), 1.04 – 1.40 (m, 8H), 1.48 – 1.82 (m, 12H), 1.83 – 2.13 (m, 4H), 2.14 – 2.33 (m, 2H), 3.10 – 3.63 (m, 6H), 4.33 – 4.65 (m, 6H), 4.67 – 5.29 (m, 2H), 5.94 – 6.62 (m, 2H), 7.37 – 7.46 (m, 2H), 7.47 – 7.54 (m, 4H), 7.75 – 7.91 (m, 4H), 8.12 – 8.20 (m, 2H), 8.44 – 8.90 (m, 6H). ^{13}C NMR (125

MHz)♦ δ 25.3, 25.7, 26.1, 28.3, 31.5, 32.7, 33.2, 36.7, 38.1, 48.8, 49.3, 49.7, 120.5, 125.4, 127.0, 129.3,* 131.3, 146.7, 167.8. LC-MS (ESI) (MeOH; method 1), RT 12.72 min, 99% purity, *m/z* = 823.6 ($[M+H]^+$). HRMS, calcd. for $C_{46}H_{62}N_8O_2S_2$: $[M+H]^+$ *m/z* 823.4510; found: 823.4541.

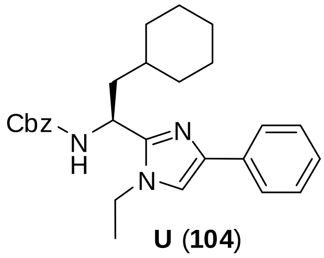

U (**104**)

*(S)-Benzyl 2-cyclohexyl-1-(1-ethyl-4-phenyl-1H-imidazol-2-yl)ethylcarbamate (**U** ≡ **104**).* The corresponding imidazole derivative **O**[4] (2.42 g, 10.0 mmol) was dissolved in anhyd DMF (16.5 mL) and treated with Cs_2CO_3 (8.15 g, 25.0 mmol). After stirring at room temperature for 30 min, bromoethane (1.31 g, 12.0 mmol) dissolved in anhyd DMF (11 mL) was added dropwise over a period of 30 min. The mixture was stirred for a further 2.5 h at room temperature, then quenched with iced H_2O (60 mL) and extracted with EtOAc (3 × 60 mL). The organic layer was washed with brine (60 mL) and H_2O (2 × 60 mL), and dried over Na_2SO_4, filtered and evaporated to dryness. The crude residue was purified by preparative CC using petroleum ether/EtOAc (10:1) to yield **104** as yellow crystals (3.80 g, 88%); mp 94–96 °C. ^{1}H NMR (500 MHz) δ 0.82 – 0.92 (m, 1H), 0.92 – 1.02 (m, 1H), 1.06 – 1.22 (m, 3H), 1.29 (t, *J* = 7.2 Hz, 3H), 1.55 – 1.69 (m, 5H), 1.72 (ddd, *J* = 13.9, 8.4, 5.4 Hz, 1H), 1.76 – 1.82 (m, 1H), 1.88 (ddd, *J* = 14.3, 9.6, 5.0 Hz, 1H), 3.95 (dq, *J* = 14.3, 7.2 Hz, 1H), 4.00 – 4.08 (m, 1H), 4.84 (td, *J* = 9.2, 5.5 Hz, 1H), 5.00 (d, *J* = 12.7 Hz, 1H), 5.06 (d, *J* = 12.7 Hz, 1H), 7.13 – 7.18 (m, 1H), 7.26 – 7.35 (m, 7H), 7.57 (s, 1H), 7.69 – 7.73 (m, 2H), 7.75 (d, *J* = 8.7 Hz, 1H). ^{13}C NMR (125 MHz) δ 16.4, 25.8, 26.0, 26.2, 32.1, 33.4, 33.8, 40.1, 41.1, 44.4, 65.4, 116.0, 124.2, 126.1, 127.6, 127.8, 128.4, 128.5, 134.8, 137.4, 138.8, 148.3, 156.0. LC-MS (ESI) (MeOH; method 1), RT 12.25 min, 97% purity, *m/z* = 432.1 ($[M+H]^+$).

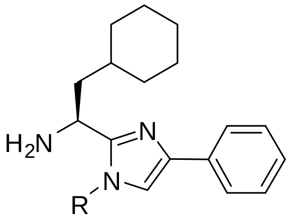

V (**105-106**)

*(S)-2-Cyclohexyl-1-(4-phenyl-1H-imidazol-2-yl)ethanamine (**V**, R = H, **105**). Typical Procedure for Cbz-Deprotection to **V**.* The corresponding imidazole derivative **O**[4] (2.42 g, 6.00 mmol) was dissolved in anhyd MeOH (60 mL) containing Pd/C (10% Pd) and was hydrogenated under atmospheric pressure at room temperature for 24 h. The catalyst was filtered off through celite and rinsed with MeOH (2 × 45 mL). The removal of the solvent *in vacuo* yielded a yellow oil without further purification (1.52 g, 94%). ^{1}H NMR (500 MHz) δ 0.79 – 0.95 (m, 2H), 1.03 – 1.21 (m, 3H), 1.31 – 1.39 (m, 1H), 1.49 (ddd, *J* = 13.7, 8.0 Hz, 6.1 Hz, 1H), 1.54 – 1.74 (m, 6H), 3.93 (dd, *J* = 8.0, 6.6 Hz, 1H), 7.12 – 7.17 (m, 1H), 7.31 (t, *J* = 7.7 Hz, 2H), 7.38 (s, 1H), 7.68 – 7.73 (m, 2H). Three proton signals are not recognizable. ^{13}C NMR (125 MHz) δ 25.8, 26.0, 26.3, 32.6, 33.4, 33.8, 45.0, 47.6, 113.3, 124.2, 125.9, 128.5, 134.5, 153.6. One carbon signal is not recognizable. LC-MS (ESI) (MeOH; method 1), RT 10.67 min, 96% purity, *m/z* = 270.1 ([M+H]$^{+}$).

*(S)-2-Cyclohexyl-1-(1-ethyl-4-phenyl-1H-imidazol-2-yl)ethanamine (**V**, R = CH_2CH_3; **106**).* From **104** (≡ **U**; 2.59 g). Yield 1.70 g, 95%. Orange oil. ^{1}H NMR (500 MHz) δ 0.81 – 0.98 (m, 2H), 1.06 – 1.24 (m, 3H), 1.35 (t, *J* = 7.3 Hz, 3H), 1.37 – 1.45 (m, 1H), 1.52 – 1.85 (m, 7H), 3.96 (dd, *J* = 8.1, 6.2 Hz, 1H), 3.97 – 4.07 (m, 2H), 7.11 – 7.16 (m, 1H), 7.28 – 7.33 (m, 2H), 7.52 (s, 1H), 7.68 – 7.73 (m, 2H). One proton signal is not recognizable. ^{13}C NMR (125 MHz)♦♦ δ 16.4, 25.8, 26.0, 26.3, 32.6, 33.6, 34.0, 45.2, 115.4, 124.1, 125.9, 128.4, 135.1, 138.4, 151.8. LC-MS (ESI) (MeOH; method 1), RT 10.99 min, 98% purity, *m/z* = 298.2 ([M+H]$^{+}$).

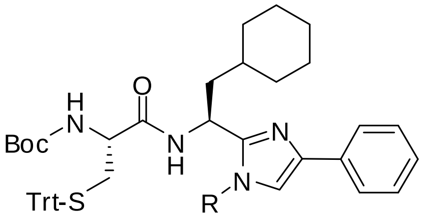

W (**107-108**)

*tert-Butyl (R)-1-((S)-2-Cyclohexyl-1-(4-phenyl-1H-imidazol-2-yl)ethylamino)-1-oxo-3-(tritylthio)propan-2-ylcarbamate (**W**, R = H, **107**). Typical Procedure to Protected Cysteine Derivatives **W**.* Boc-L-Cys(Trt)-OH (2.09 g, 4.50 mmol) and DCC (928 mg, 4.50 mmol) were

stirred in anhyd CH_2Cl_2 (28 mL) under argon atm for 30 min. Compound **105** (404 mg, 1.50 mmol) dissolved in anhyd CH_2Cl_2 (18 mL) and DIPEA (0.26 mL, 194 mg, 1.50 mmol) were added and the reaction mixture was stirred at room temperature and argon atm for 48 h. After removing the solvent *in vacuo*, the residue was treated with a mixture of petroleum ether/EtOAc (7:3) (75 mL). The white urea was filtered off, the filtrate was evaporated to dryness and the crude oily residue was purified by preparative CC on silica gel using a gradient of petroleum ether/EtOAc (4:1) to (7:3) to obtain **107** as white solid (858 mg, 80%); mp 183–185 °C. ^{1}H NMR (500 MHz) δ 0.74 – 0.93 (m, 2H), 0.98 – 1.14 (m, 2H), 1.16 – 1.26 (m, 1H), 1.36 (s, 9H), 1.49 – 1.75 (m, 8H), 2.33 (dd, *J* = 12.0, 5.5 Hz, 1H), 2.39 (dd, *J* = 12.0, 8.7 Hz, 1H), 3.85 – 3.97 (m, 1H), 4.99 (td, *J* = 9.0, 6.1 Hz, 1H), 7.01 (d, *J* = 8.5 Hz, 1H), 7.13 – 7.37 (m, 18H), 7.42 (s, 1H), 7.69 (d, *J* = 7.7 Hz, 2H), 7.83 (d, *J* = 8.6 Hz, 1H), 11.87 (s, 1H). ^{13}C NMR (125 MHz) δ 25.6, 25.8, 26.1, 28.2, 32.0, 33.2, 33.4, 33.8, 41.8, 44.6, 53.9, 66.0, 78.6, 112.5, 124.3, 126.2, 126.8, 128.1, 128.5, 129.2, 134.9, 139.6, 144.4, 149.0, 155.1, 169.5. LC-MS (ESI) (MeOH; method 1), RT 13.62 min, 98% purity, *m/z* = 715.7 ($[M+H]^+$).

*tert-Butyl (R)-1-((S)-2-Cyclohexyl-1-(1-ethyl-4-phenyl-1H-imidazol-2-yl)ethylamino)-1-oxo-3-(tritylthio)propan-2-ylcarbamate (**W**, R = CH_2CH_3; **108**).* From **106** (446 mg). CC using petroleum ether/EtOAc (4:1). Yield 1.05 g, 94%. White solid; mp 157–160 °C. ^{1}H NMR (500 MHz) δ 0.73 – 0.98 (m, 2H), 0.98 – 1.16 (m, 3H), 1.20 (t, *J* = 7.2 Hz, 3H), 1.25 – 1.31 (m, 1H), 1.33 (s, 9H), 1.50 – 1.64 (m, 4H), 1.65 – 1.80 (m, 2H), 1.87 (ddd, *J* = 14.2, 9.7 Hz, 4.7 Hz, 1H), 2.25 (dd, *J* = 11.8, 5.5 Hz, 1H), 2.32 (dd, *J* = 11.9, 8.6 Hz, 1H), 3.75 – 3.84 (m, 1H), 3.87 – 3.98 (m, 2H), 5.07 (td, *J* = 9.4, 5.1 Hz, 1H), 6.90 (d, *J* = 8.7 Hz, 1H), 7.14 – 7.29 (m, 16H), 7.32 (dd, *J* = 8.2, 7.2 Hz, 2H), 7.57 (s, 1H), 7.69 – 7.74 (m, 2H), 7.97 (d, *J* = 8.9 Hz, 1H). ^{13}C NMR (125 MHz)♦ δ 16.2, 25.6, 25.9, 26.2, 28.2, 32.0, 33.4, 33.5, 34.1, 41.0, 41.6, 53.9, 65.9, 78.5, 116.0, 124.2, 126.1, 126.8, 128.1, 128.5, 129.1, 134.8, 138.8, 144.4, 147.5, 154.9, 169.6. LC-MS (ESI) (MeOH; method 1), RT 13.27 min, 99% purity, *m/z* = 743.5 ($[M+H]^+$).

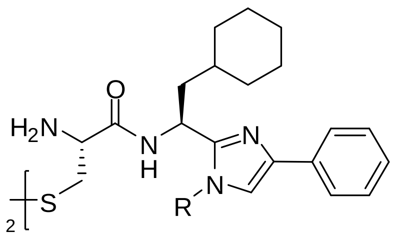

28 R = H
29 R = CH_2CH_3

*(2R,2'R)-3,3'-Disulfanediylbis(2-amino-N-((S)-2-cyclohexyl-1-(4-phenyl-1H-imidazol-2-yl)ethyl)propanamide) Tetrahydrochloride (**28**).* Compound **107** (715 mg, 1.00 mmol) was dissolved in anhyd CH_2Cl_2 (8 mL) under argon atm and a mixture of triisopropylsilane (2.08

mL, 1.58 g, 10.0 mmol) and TFA (8 mL) was added. After stirring at room temperature for 18 h, the solvent was evaporated to dryness without heating. The residue was treated with *n*-hexane (40 mL), followed by decanting the organic phase. The remaining oil was dissolved in anhyd EtOAc (35 mL) and treated with 1 M HCl in EtOAc (4 mL). The precipitate was filtered off, washed with anhyd EtOAc (2 × 30 mL) and dried *in vacuo*. The crude salt was dissolved in MeOH (30 mL) containing H_2O (0.6 mL). A solution of iodine (254 mg, 1.00 mmol) in MeOH (30 mL) was added dropwise under vigorous stirring and the reaction mixture was stirred at room temperature for 2 h. After evaporating the solvent *in vacuo*, the crude residue was purified by means of preparative CC using a gradient of $CHCl_3$ / 7 N NH_3 in MeOH (100:2.5) to (100:3.75). The resulting oil was redissolved in anhyd EtOAc (35 mL) and treated with 1 M HCl in EtOAc (4 mL). The precipitate was filtered off, washed with anhyd EtOAc (2 × 30 mL) and dried *in vacuo*. The crude salt was dissolved in MeOH (147 mg/mL) and purified through semi-preparative HPLC with a gradient from 30% MeCN + 0.05% TFA / 70% H_2O + 0.05% TFA to 60% MeCN + 0.05% TFA / 40% H_2O + 0.05% TFA within 15 min (100 µL; 14.0 mL/min; 255 nm), followed by volatile evaporation and lyophilization. The residue was redissolved in anhyd EtOAc (35 mL) and treated with 1 M HCl in EtOAc (4 mL). The precipitate was again filtered off, washed with anhyd EtOAc (2 × 30 mL) and dried *in vacuo* to obtain compound **28** as a white solid (53.3 mg, 12%); mp > 224 °C decomp. ^{1}H NMR (500 MHz) δ 0.87 – 0.99 (m, 4H), 1.07 – 1.24 (m, 6H), 1.34 – 1.44 (m, 2H), 1.55 – 1.76 (m, 10H), 1.82 – 1.91 (m, 2H), 1.91 – 2.00 (m, 2H), 3.12 (dd, *J* = 14.2, 8.8 Hz, 2H), 3.68 (dd, *J* = 14.4, 4.2 Hz, 2H), 4.32 (dd, *J* = 8.8, 4.3 Hz, 2H), 5.20 (dt, *J* = 8.9, 6.0 Hz, 2H), 7.41 (t, *J* = 7.4 Hz, 2H), 7.48 (t, *J* = 7.6 Hz, 4H), 7.88 (d, *J* = 7.7 Hz, 4H), 8.04 (s, 2H), 8.55 (s, 6H), 9.54 (d, *J* = 5.8 Hz, 2H). The signal for the imidazole-N*H* is not recognizable. ^{13}C NMR (125 MHz)♦ δ 25.5, 25.7, 26.1, 31.8, 32.9, 33.3, 38.0, 44.7, 51.1, 115.2, 125.7, 127.2, 129.3,* 132.8, 148.4, 167.6. LC-MS (ESI) (MeCN; method 1), RT 13.07 min, 100% purity, *m/z* = 743.5 ($[M+H]^+$). HRMS, calcd. for $C_{40}H_{54}N_8O_2S_2$: $[M+H]^+$ *m/z* 743.3884; found: 743.3868.

*(2R,2'R)-3,3'-Disulfanediylbis(2-amino-N-((S)-2-cyclohexyl-1-(1-ethyl-4-phenyl-1H-imidazol-2-yl)ethyl)propanamide) Tetrahydrochloride (**29**).* Compound **108** (743 mg, 1.00 mmol) was dissolved in anhyd CH_2Cl_2 (8 mL) under argon atm and a mixture of triisopropylsilane (2.08 mL, 1.58 g, 10.0 mmol) and TFA (8 mL) was added. After stirring at room temperature for 18 h, the solvent was evaporated to dryness without heating. The residue was treated with *n*-hexane (40 mL), followed by decanting the organic phase. The remaining oil was dissolved in anhyd EtOAc (35 mL) and treated with 1 M HCl in EtOAc (4

mL). The precipitate was filtered off, washed with anhyd EtOAc (2 × 30 mL) and dried *in vacuo*. The crude salt was dissolved in MeOH (30 mL) containing H_2O (0.6 mL). A solution of iodine (254 mg, 1.00 mmol) in MeOH (30 mL) was added dropwise and the reaction mixture was stirred at room temperature for 2 h. After evaporating the solvent *in vacuo*, the crude residue was purified by means of preparative CC using a gradient of $CHCl_3$ / 7 N NH_3 in MeOH (100:2.5) to (100:3.75). The resulting oil was redissolved in anhyd EtOAc (35 mL) and treated with 1 M HCl in EtOAc (4 mL). The precipitate was filtered off, washed with anhyd EtOAc (2 × 30 mL) and dried *in vacuo* to obtain compound **29** as a yellowish solid (151 mg, 32%); mp > 230 °C decomp. ^{1}H NMR (500 MHz) δ 0.84 – 1.01 (m, 4H), 1.04 – 1.29 (m, 8H), 1.45 (t, *J* = 7.3 Hz, 6H), 1.53 – 1.77 (m, 10H), 1.84 (ddd, *J* = 14.0, 8.3, 5.6 Hz, 2H), 2.13 (ddd, *J* = 14.3, 9.6, 5.2 Hz, 2H), 3.17 (dd, *J* = 14.1, 7.8 Hz, 2H), 3.55 (dd, *J* = 14.2, 4.9 Hz, 2H), 4.27 (q, *J* = 7.3 Hz, 4H), 4.29 – 4.36 (m, 2H), 5.23 (dt, *J* = 9.5, 5.8 Hz, 2H), 7.38 – 7.45 (m, 2H), 7.48 (dd, *J* = 8.5, 6.9 Hz, 4H), 7.86 – 7.97 (m, 4H), 8.22 (s, 2H), 8.67 (s, 6H), 9.76 (d, *J* = 5.7 Hz, 2H). ^{13}C NMR (125 MHz)♦δ 15.2, 25.5, 25.7, 26.0, 31.7, 33.1, 33.4, 38.4, 42.9, 43.3, 51.2, 117.9, 125.8, 127.0, 129.2, 129.4, 132.6, 146.7, 167.5. LC-MS (ESI) (MeOH; method 1), RT 13.27 min, 95% purity, *m/z* = 799.3 ($[M+H]^+$). HRMS, calcd. for $C_{44}H_{62}N_8O_2S_2$: $[M+H]^+$ *m/z* 799.4510; found: 799.4494.

2.3. Chiral HPLC Analysis of Compounds 1, 2, 3, and 4

The chiral HPLC analysis was performed at a Knauer AZURA system equipped with a DAD 6.1L detector (190-600 nm). A chiral stationary phase, CHIRALPAK IA (Daicel) with particle size 5 µm, column size 4.6 × 250 mm, an isocratic mixture of methanol + 0.01% diethylamine, a flow rate of 1.0 mL/min and a column temperature of 25 °C were used. The injection volume was 10 µL.

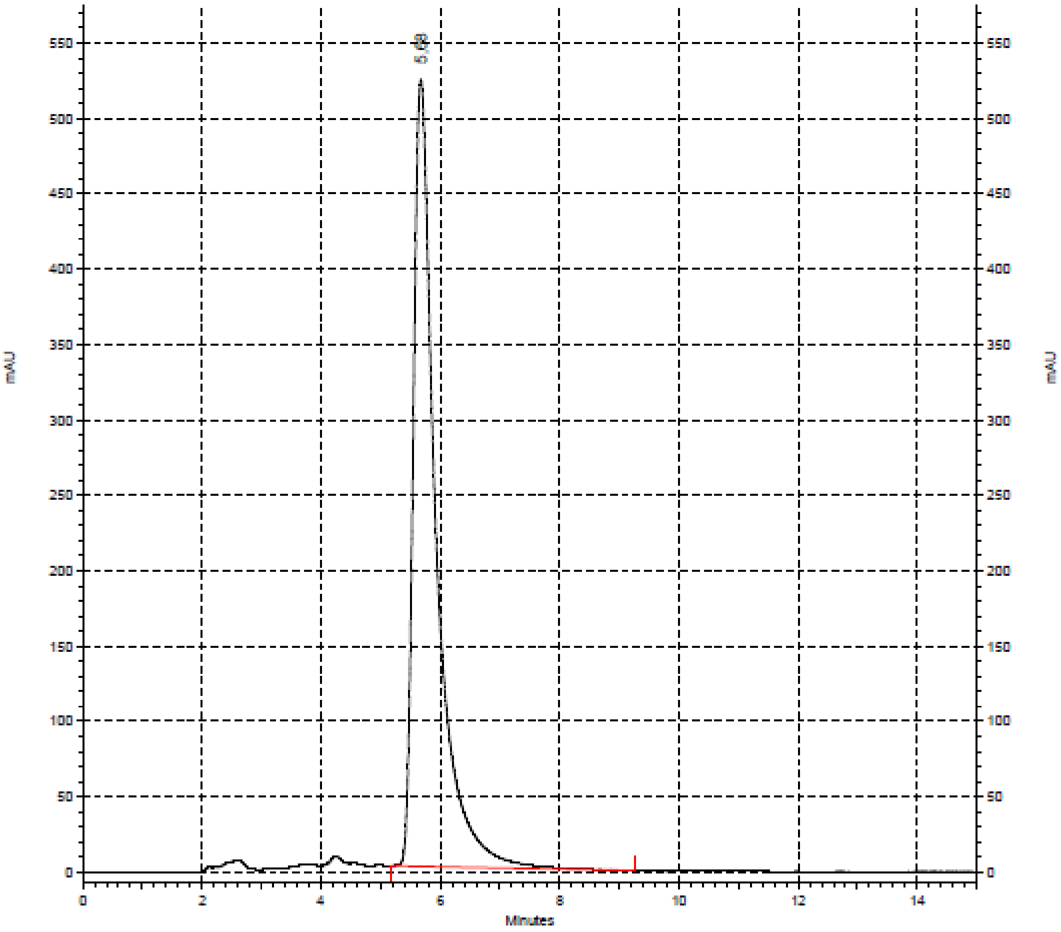

Compound 1 (2*R*, 8'*S*) (0.43 mg in 0.86 mL MeCN/H_2O 1:1)

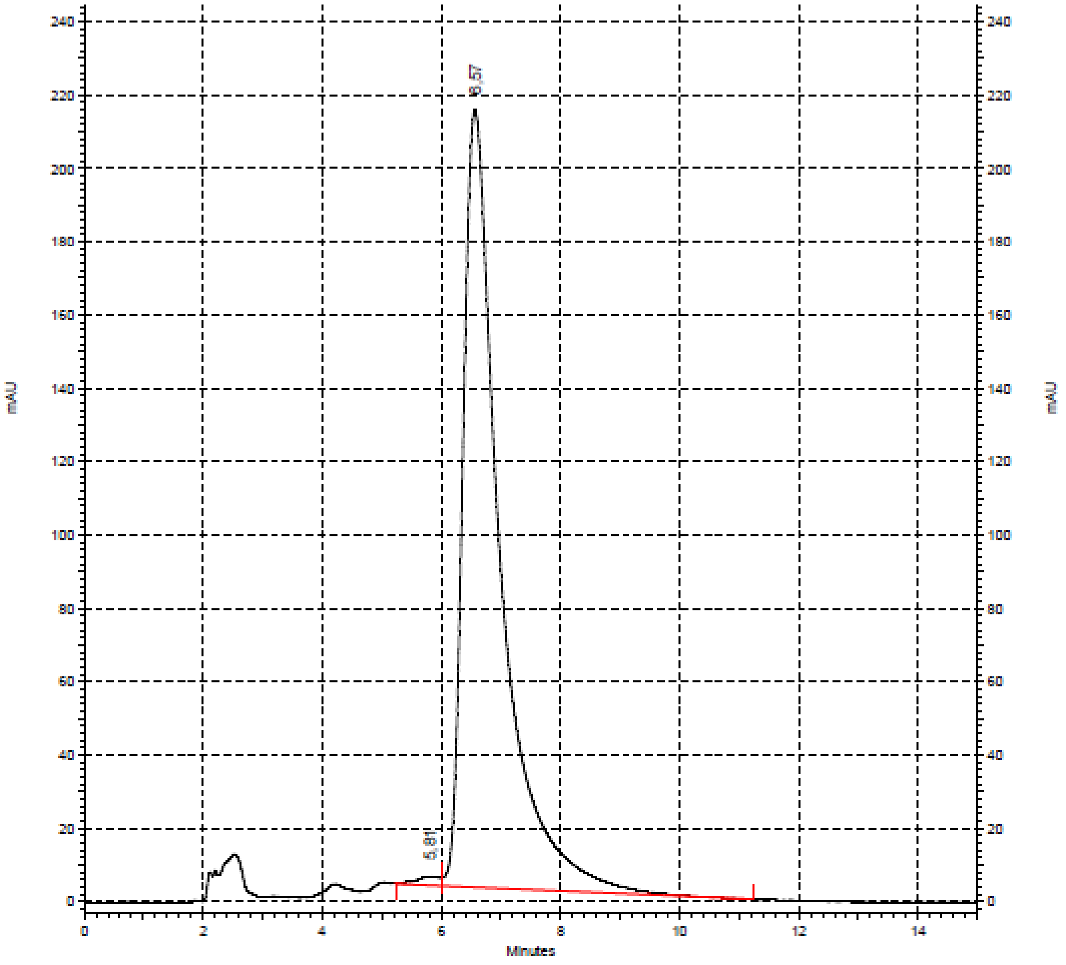

Compound 4 (2*S*, 8'*R*) (0.70 mg in 1.40 mL MeCN/H_2O 1:1)

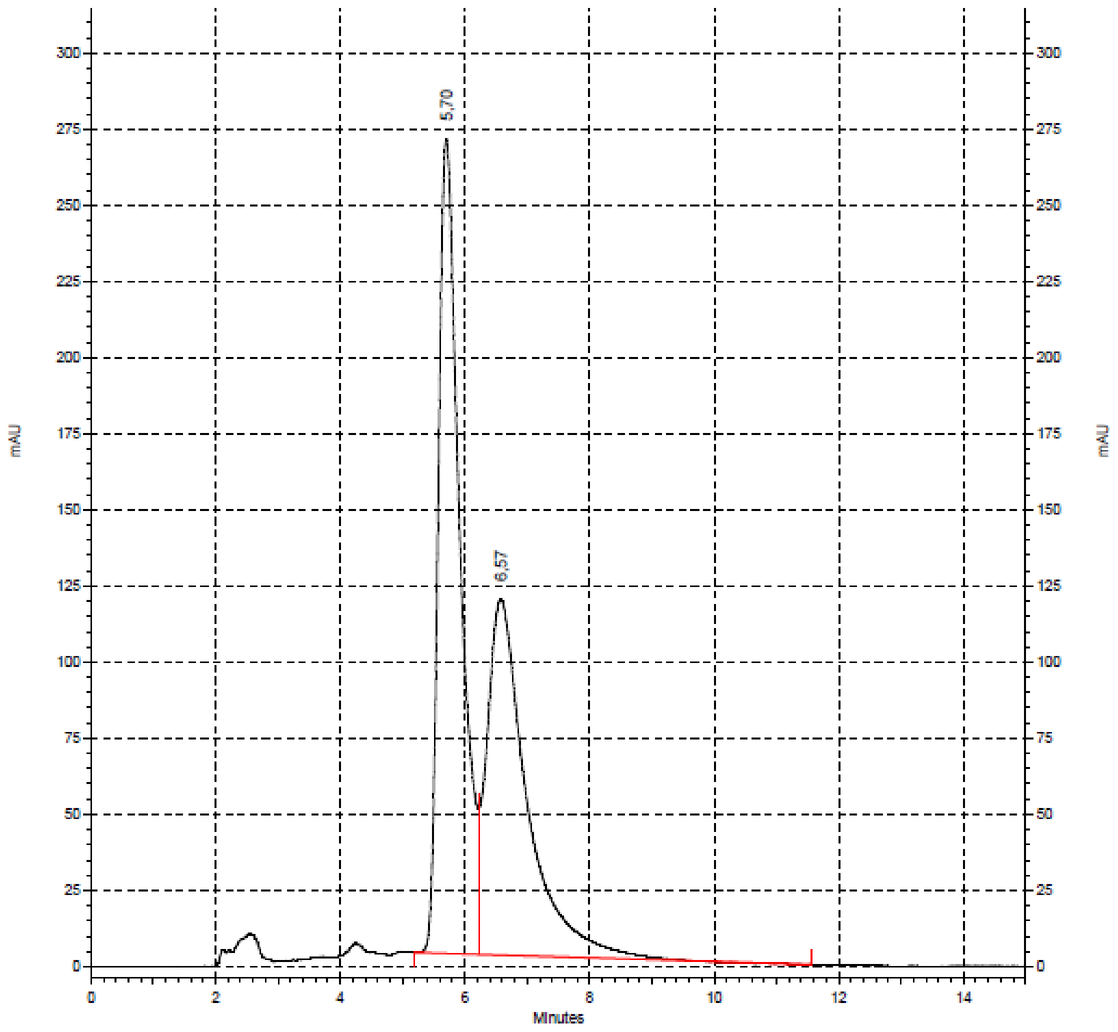

Pseudoracemic mixture of compound **1** and compound **4**

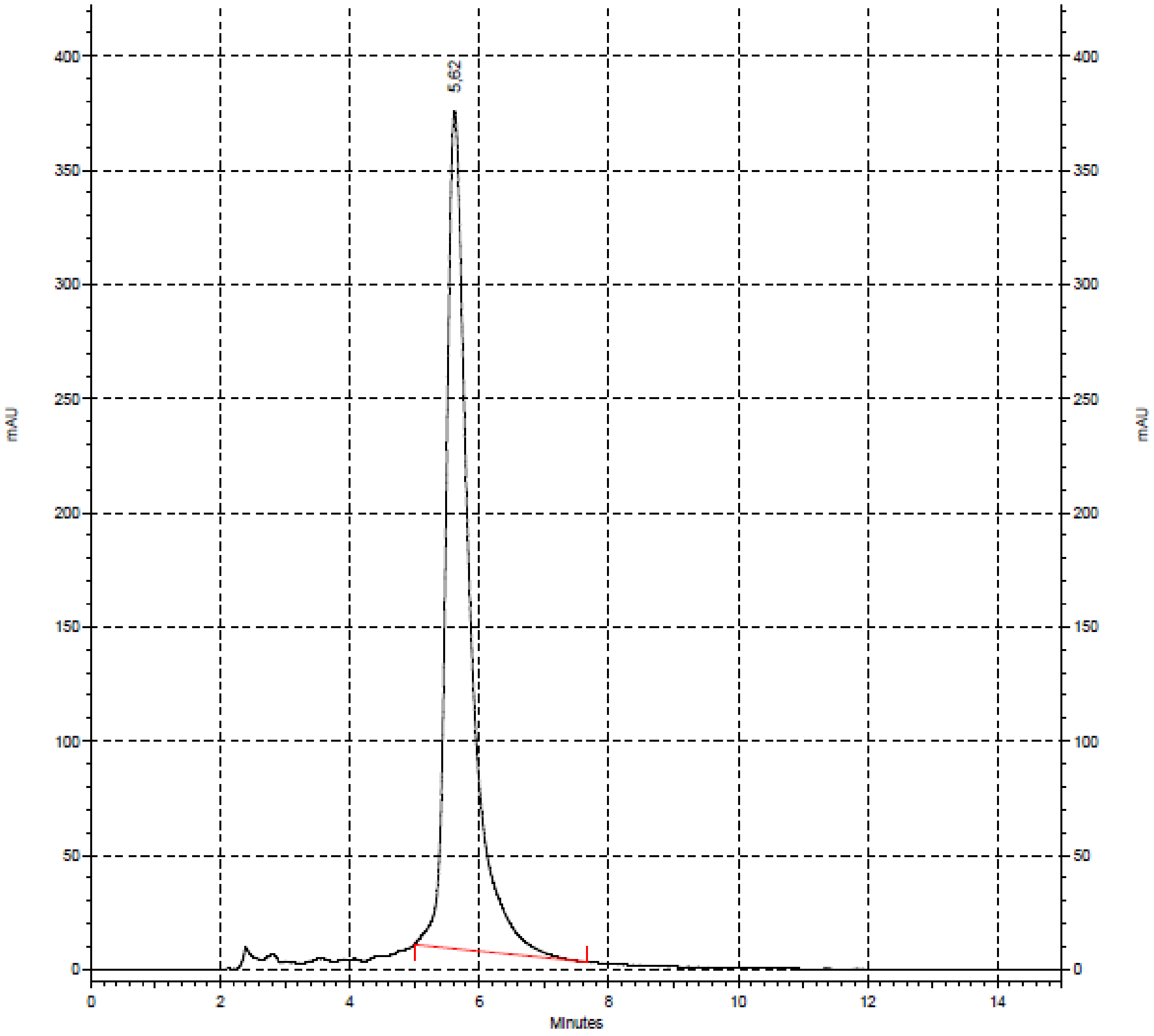

Compound 2 (2*S*, 8'*S*) (1.48 mg in 1.50 mL MeCN/H_2O 1:1)

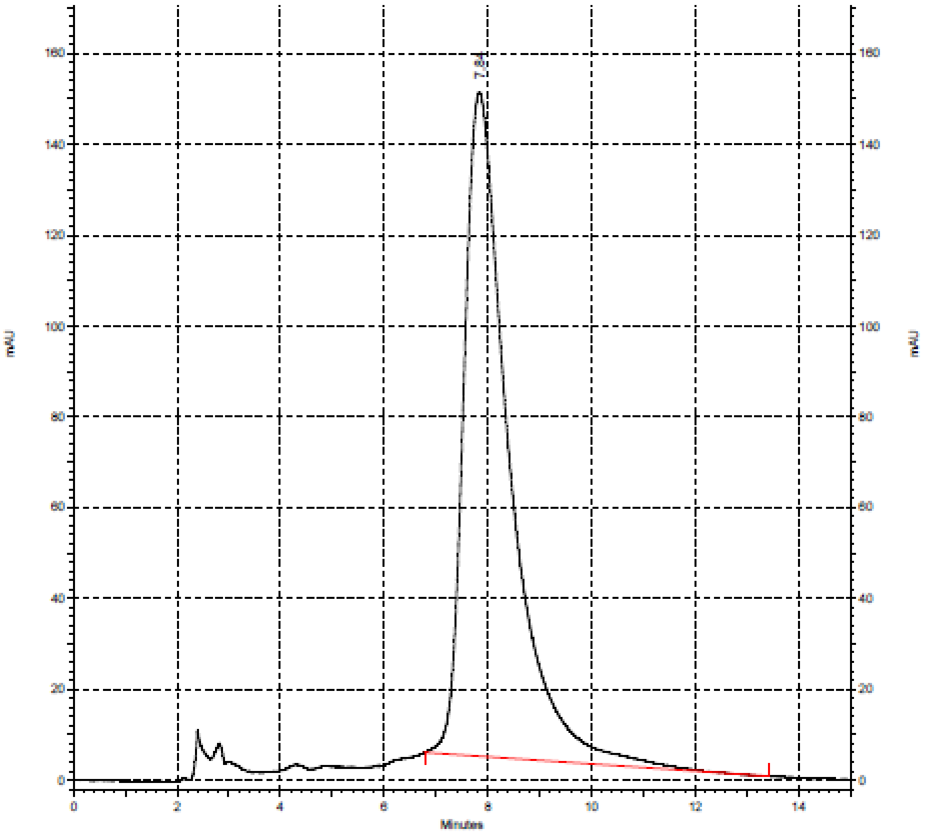

Compound 3 (2*R*, 8'*R*) (1.36 mg in 1.40 mL MeCN/H_2O 1:1)

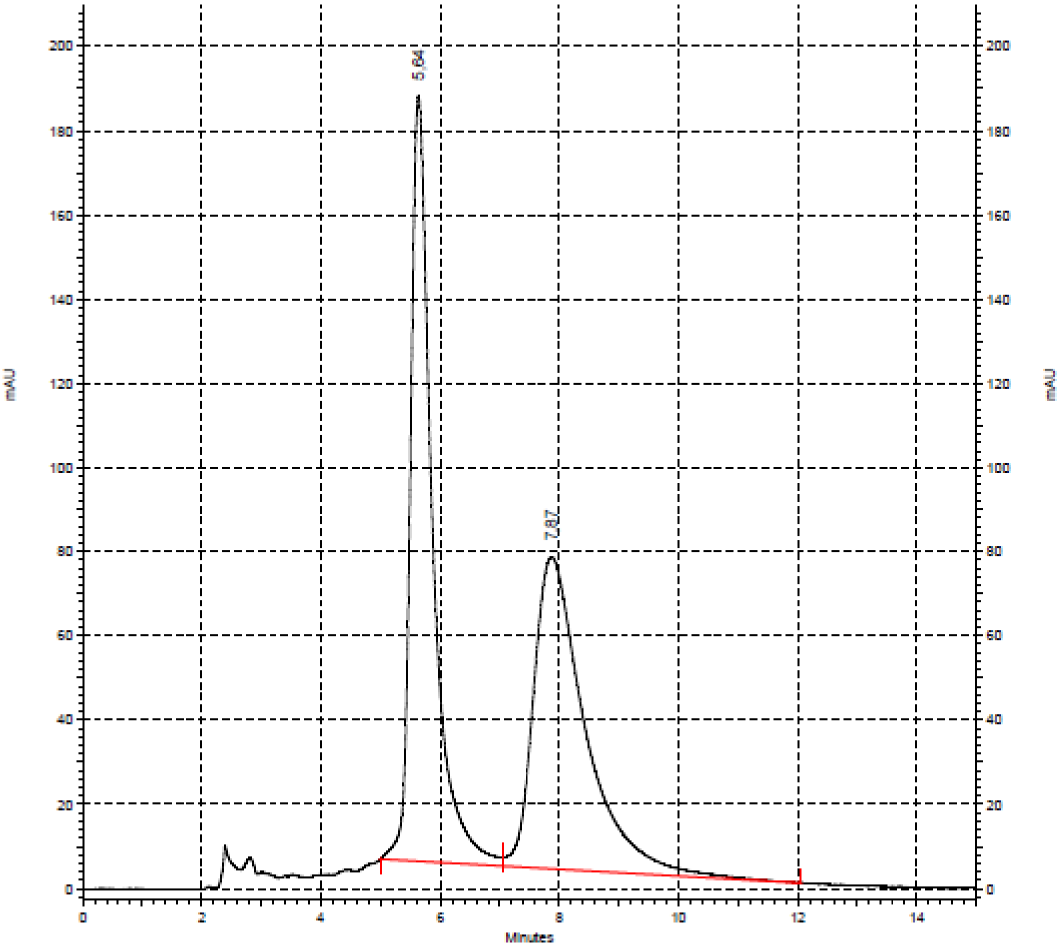

Pseudoracemic mixture of compound **2** and compound **3**

3. Abbreviations

Acm, acetamidomethyl; ATCC, American Type Culture Collection; Boc, *tert*-butyloxycarbonyl; cAMP, cyclic adenosine-3',5'-monophosphate; Cbz, benzyloxycarbonyl; CCh, carbachol; CHO, Chinese hamster ovary; COS, CV-1 in origin with SV40 genes; DAG, diacylglycerol; H-Dap-OH, 1,2-diaminopropionate; DCC, *N*,*N'*-dicyclohexylcarbodiimide; DIPEA, *N*,*N*-diisopropylethylamine; Fmoc, fluorenylmethoxycarbonyl; G protein, heterotrimeric guanine-nucleotide binding protein; GPCR, G protein-coupled receptor; HATU, *O*-(7-azabenzotriazol-1-yl)-*N*,*N*,*N'*,*N'*-tetramethyluronium-hexafluorphosphat; H-Hcy-OH, homocysteine; HEK, human embryonic kidney; HTRF, homogenous time-resolved fluorescence; IP1, *myo*-inositol 1-phosphate; IP2, *myo*-inositol 1,4-bisphosphate; IP3, *myo*-inositol 1,4,5-trisphosphate; KO, knockout; mAChR, muscarinic acetylcholine receptor; PBS, phosphate-buffered saline; H-Pen-OH, penicillamine; PIP2, phosphatidylinositol-4,5-bisphosphate; PL, phospholipase; PLCβ, phospholipase C-β; Trt, trityl; w/o, without; wt, wild-type.

4. Author Contributions

M.G. and E.K. conceived the study. J.K. and M.G. designed compounds. J.K. and L.R. synthesized compounds. T.B., S.A., and K.K. performed biological studies. All authors analyzed data. J.K., E.K. and M.G. wrote the manuscript. B.K.F., E.K. and M.G. supervised the project.

5. References

[1] J. Schmidt, N. J. Smith, E. Christiansen, I. G. Tikhonova, M. Grundmann, B. D. Hudson, R. J. Ward, C. Drewke, G. Milligan, E. Kostenis, T. Ulven, *J. Biol. Chem.* **2011**, *286*, 10628–10640.

[2] R. Reher, T. Kühl, S. Annala, T. Benkel, D. Kaufmann, B. Nubbemeyer, J. P. Odhiambo, P. Heimer, C. A. Bäuml, S. Kehraus, M. Crüsemann, E. Kostenis, D. Tietze, G. M. König, D. Imhof, *ChemMedChem* **2018**, *13*, 1634–1643.

[3] G. C. Stelakatos, A. Paganou, L. Zervas, *J. Chem. Soc., Perkin Trans. 1* **1966**, *13*, 1191–1199.

[4] J. Küppers, M. Hympánová, T. Keuler, A. J. Schneider, G. Schnakenburg, M. Gütschow, *Synthesis* **2019**, *51*, 1961–1968.

[5] A. L. Schmitz, R. Schrage, E. Gaffal, T. H. Charpentier, J. Wiest, G. Hiltensperger, J. Morschel, S. Hennen, D. Häußler, V. Horn, D. Wenzel, M. Grundmann, K. M. Büllesbach, R. Schröder, H. H. Brewitz, J. Schmidt, J. Gomeza, C. Galés, . B. K. Fleischmann, T. Tüting, D. Imhof, D. Tietze, M. Gütschow, U. Holzgrabe, J. Sondek, T. K. Harden, K. Mohr, E. Kostenis, *Chem. Biol.* **2014**, *21*, 890–902.

[6] J. Küppers, T. Benkel, S. Annala, G. Schnakenburg, E. Kostenis, M. Gütschow, *Med. Chem. Commun.* **2019**, *10*, 1838–1843.
